# Mimicking Extradiol Dioxygenase Reactivity on Iridium

**DOI:** 10.1021/jacs.5c23353

**Published:** 2026-06-10

**Authors:** Alexander G. Arnette, Anant Kumar Jain, Alexey Silakov, Karen I. Goldberg, Jonathan L. Kuo

**Affiliations:** † Department of Chemistry, 8082 The Pennsylvania State University, University Park, Pennsylvania 16802, United States; ‡ Department of Chemistry, 6572 University of Pennsylvania, Philadelphia, Pennsylvania 19104, United States

## Abstract

Extradiol dioxygenases catalyze the cleavage of benzenediol (catechol) or vicinal aminophenols via oxygen atom insertion into the 2,3-C–C bond. These reactions are most often proposed to proceed through the migratory rearrangement of a d^6^ alkylperoxide, generating the corresponding d^6^ ring-expanded product. However, regiospecific insertion remains a rare outcome among synthetic model complexes. Here, a dioxygenated Ir complex (**2**) converts to (a) the paramagnetic metallatrioxolane (**3**) and (b) oxygen atom-inserted products (**4**) and (**5**); all three complexes are third-row metal analogues of enzymatic intermediates. The conversion of **2** to **3** was triggered by an H• abstraction, generating a third-row metallatrioxolane that is one electron reduced from the canonical d^6^ alkylperoxide. Alternatively, photolysis of **2** (467 nm) results in ring-expanded product **4**, from which an H• can be abstracted to generate **5**. This latter complex is also one electron reduced relative to the canonical ring-expansion product. Because extradiol mechanisms were largely defined using Fe­(II) metallocofactors, **3** and **5** may be especially relevant to the known Co­(II)-accepting variants. We propose these states became synthetically accessible via the incorporation of a catechol-like substrate into the larger, multidentate ligand **L1**. This perturbation enhances the affinity of **L1** (and related intermediates) to the metal. The same perturbation may have also been key in characterizing the first κ^2^-bound, dianionic ortho ester ligand—the observed binding mode in the X-ray structure of **5**. Broadly, we propose that connecting a biological dioxygen complexes to nonheme oxygenase-like intermediates provides useful insights for regiospecific aerobic oxygenations.

## Graphical abstract

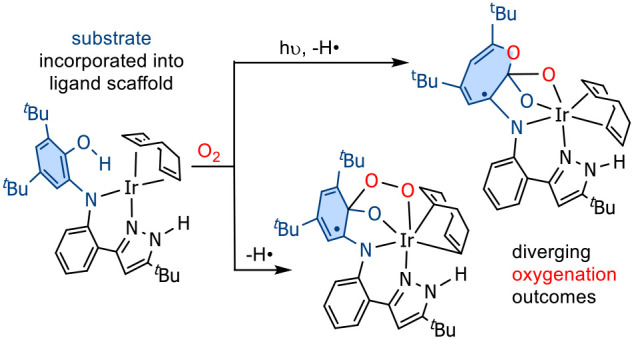

## Introduction

Aerobic oxidations—using O_2_ as the oxidant—have significant advantages relative to their nonaerobic counterparts.^1^ For example, monooxygenation, defined as the incorporation of one O atom into a substrate, should result in H_2_O as the only stoichiometric byproduct (substrate + O_2_ + 2e^–^ + 2H^+^ → substrate­[O] + H_2_O). Dioxygenations can be perfectly atom-economical (e.g., 2 substrate + O_2_ → 2 substrate­[O]). However, selectivity is a central challenge to catalyst development because virtually all aerobic oxidations are exergonic.^2–9^

The possibility of atom-efficient aerobic oxidations spurs interest in dioxygenated transition metal complexes. Tetracoordinate Ir­(I) complexes (sometimes referred to as “Vaska-like”) are curious because they reversibly add dioxygen to generate the corresponding Ir­(III)-peroxides.^10^ Reversibility suggests that these Ir­(III)-peroxides retain much of the oxidizing ability of O_2_, but for whatever reason, these (and similar) peroxides rarely promote selective oxygen atom transfer.^3,4^

The principles underlying selective oxygen atom transfer are encoded in the mechanisms of metalloenzymatic^11–13^ oxygenases.^14–16^ For example, catechol dioxygenases promote the regiospecific ring cleavage of 1,2-benzenediols by inserting an oxygen atom either between (intradiol) or outside (extradiol) the two hydroxyl groups (monooxygenation). This ring expansion results in the loss of aromaticity and, therefore, various opportunities for subsequent rearrangements. Some of these rearrangements incorporate the second oxygen atom of O_2_ (dioxygenation).^17,18^

The mechanisms for both intra- and extradiol variants begin with the substrate binding to the metal (**I**_**intra**_ and **I**_**extra**_ in Figure 1). Different coordination geometries for the metal-substrate complex affect how O_2_ approaches, resulting in distinct bridging alkylperoxides (also called metallatrioxolanes, **II**_**intra**_ and **II**_**extra**_ in Figure 1). These peroxides position either the intradiol 1,2- or the extradiol 2,3-C–C (σ_C–C_)^19–22^ to interact with the antibonding $\sigma_{O-O}^{*}$ ; the regiospecificity can then be rationalized analogously to the Baeyer–Villiger rearrangement.^21–23^ For the intradiol cases, this analogy is further supported by a crystallographically characterized lactone-bound state, which presumably succeeds the metallatrioxolane (**III**_**intra**_). A similar intermediate has been characterized for an “extradiol” aminophenol dioxygenase (**III**_**extra**_),^24,25^ which performs the corresponding ring expansion on aromatic substrates with vicinal hydroxyl and amino groups (alkylperoxides like **II**_Fied_). These accumulating observations may suggest that bridging alkylperoxides are well-suited for selective oxygen atom transfer.^26,27^

**Figure 1:**
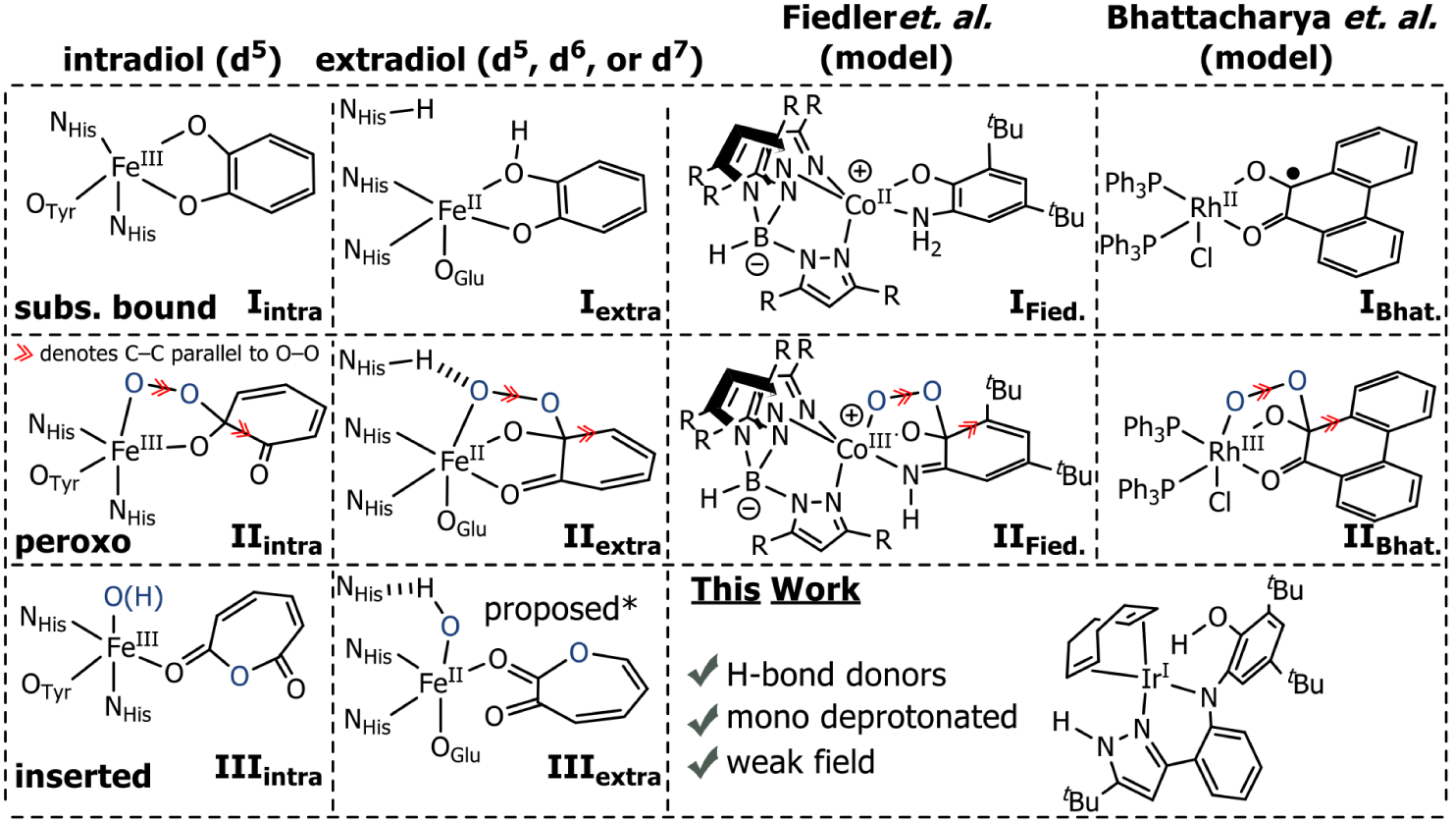
Comparing X-ray crystal structures for substrate-bound (top row), metallatrioxolane/alkylperoxo (middle row), and inserted states (bottom row). From left to right, drawings display enzymatic intradiol (left column, PDB codes 4WHS and 4WHQ),^28^ enzymatic extradiol (center-left column, PDB codes 4GHH and 2IGA);^29^ model complexes by Fiedler et al. (center-right column)^30^ and Bhattacharya et al.,^31^ which do not advance past the metallatrioxolane. Red arrows denote parallel O–O and C–C bonds, a requirement for Baeyer–Villiger-like selectivity (*σ*_C–C_ attacking $\sigma_{O-O}^{*}$ ). **III_extra_** is drawn by analogy to an “extradiol” aminophenol dioxygenase (PDB code 6VIA).^32^ The bottom right depicts our approach to modeling the extradiol case.

Despite the simplicity of a “Baeyer-Villiger-like” framework, replicating the ring-cleaving dioxygenase reaction within synthetic complexes has proven to be a multifaceted challenge. Consider the net two-electron reduction of O_2_ by (**subs. bound**) metal to generate an alkylperoxide (**peroxo**). Intradiol variants predominantly use Fe­(III) as the metallocofactor,^33–36^ whereas extradiol variants accept Mn­(II), Fe­(II), or Co­(II).^37–39^ None of these metals tend to supply two electrons in their stoichiometric reactions.^40–42^ Instead, the metallocofactors cooperate with the redox-active catechol­(-like) substrate, which can supply an additional one or two electrons via oxidation to the corresponding semiquinone or benzoquinone (catechol/metal serving as a “pool” of two electrons).^43,44^ However, the various Lewis acidities and redox potentials of the metal/substrate result in different elementary steps for the net two-electron reduction of O_2_.^45–48^ Some cases, for example, proceed through a side-on^20^ dioxygen, and others through an end-on one.^38,49,50^ Similar arguments likely apply to the conversion of the alkylperoxide (**peroxo**) to the ring-expanded product (**inserted**).^51–53^ These subtle variations may confound efforts to replicate a given oxygenation reaction or invert selectivity unexpectedly. For example, synthetic monohydrogen catecholate [(6-Me_3_-TPA)­Fe^II^(DBCH)]^+^ is selectively oxygenated to intradiol cleavage products,^54^ whereas the same fragment bonded to an amidophenolate [(6-Me_3_-TPA)­Fe^II^(4-^*t*^Bu-HAP)]^+^ is selective for extradiol ones (6-Me_3_-TPA = tris­(6-methyl-2-pyridylmethyl)­amine; DBCH = monoanionic 3,5-di-*tert-*butylcatecholate; 4-^*t*^Bu-HAP = monoanionic 2-amino-4*-tert*-butylphenolate).^55^

Efforts to assemble or isolate a metallatrioxolane and then subsequently trigger a well-defined rearrangement (**peroxo** to **inserted**) have thus far resulted in three X-ray structures of “extradiol” alkylperoxides.^56^ These three alkylperoxides—including **II**_**Fied**_ and **II**_**Bhat**_—were ultimately generated via the oxygenation of a d^6^ catecholate (**I**_**Fied**_ or **I**_**Bhat**_; the former first underwent oxidation by one electron) in sequences that resemble the consensus mechanism for extradiol dioxygenases. Although the resulting d^6^ metallatrioxolanes position the extradiol C–C bond to attack the O–O, none of them insert.^30,31,57^

We highlight two factors that may be relevant to advancing past the alkylperoxide. First, for **I**_**extra**_, catechol is bound to d^6^ Fe­(II) in a bidentate, monoanionic mode^58^ in an overall weak ligand field environment.^59,60^ Among synthetic complexes, this coordination mode requires the use of strong field ligands^61,62^ (e.g., **I**_**Fied**_).^63–69^ These perturbations may, for example, make the reduction of O_2_ too exergonic; the system may have lost too much energy to perform the subsequent rearrangement (i.e., an overpotential). Second, the metalloenzymes feature a protic histidine residue that is positioned to interact with ligands in the apical site—e.g., the peroxide ligand of **II**_**extra**_.^37^ Mutants that lack this histidine bind catechol and O_2_ at a rate comparable to the wild type but are slower catalysts overall.^49^ However, it is unclear how to productively place a corresponding protic feature in a model complex.^70^ In many ways, these challenges are general to synthetic modeling, as the inclusion of any “primary” or “secondary” coordination sphere component must be balanced against synthetic practicality.

Here, we will explore a strategy that can address challenges in replicating “primary” and “secondary” coordination sphere components. Tethering an aminophenol—a ring-cleaving dioxygenase substrate—to additional coordinating groups can stabilize weakly associating binding modes via the chelate effect. This perturbation may permit monoanionic coordination modes in weaker ligand field environments.^71–73^ If those coordinating groups are both protic and mobile (e.g., through bond rotations), then they might also mimic the function of the apical histidine residue. We therefore synthesized ligand **L1**—isolated in 56% yield over three steps—by joining our previously reported aniline-pyrazole fragment^74^ to 3,5-di-*tert-*butyl-catechol. The resulting aminophenol^75^ ligand **L1** can coordinate in either a bidentate or a tridentate mode, with protic groups capable of repositioning through C–N bond rotations. This ligand design strategy will be validated via the oxygenation of a Vaska-like Ir complex because the resulting peroxide is expected to retain most of the intrinsic oxidizing power of O_2_ (the absence of exergonicity in oxygenation implies little energy will be lost from the system).

## Results and Discussion

The metallatrioxolane of an extradiol dioxygenase (**II**_**extra**_) is composed of the substrate, a d^6^ metal, and a doubly reduced O_2_ ligand. A corresponding d^6^ assembly can be accessed via oxygenation of a neutral d^8^ square-planar Ir complex **1** to the corresponding d^6^-peroxo.^76^ Using the metal to reduce O_2_ instead of the aminophenolate ligand is expected to require a subsequent oxidation state adjustment (*vide infra*).

Metalation of **L1** with [Ir­(COD)­(MeCN)_2_]­[BF_4_] (COD = 1,5-cyclooctadiene), followed by deprotonation with NEt_3_ afforded the desired Ir­(I) complex **1** in 84% yield (Scheme 1). The X-ray structure of **1** indicates that **L1** is bound in a monoanionic κ^2^-mode through the aniline nitrogen and a pyrazole nitrogen, with the phenolic unit free to rotate (Figure 2).

**Scheme 1:**
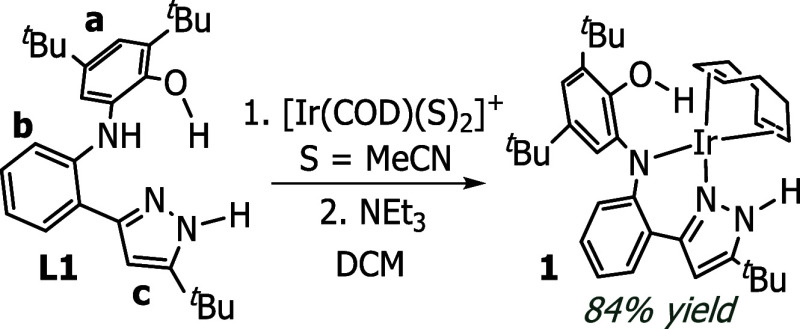
Ligation of L1 to Form 1; L1 Is Comprised of Three Rings, an Aminophenol (a), an Aniline (b), and a Pyrazole (c)

**Figure 2:**
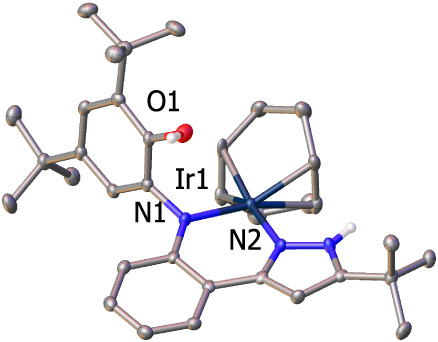
Thermal ellipsoid plot of **1** is presented at the 50% probability level. Carbon-bound hydrogen atoms are omitted for clarity.

Dichloromethane solutions of **1** cleanly convert to **2** when exposed to 1 atm of O_2_ (Scheme 2).^77^ In this work, diagnostic ^1^H NMR spectroscopy resonances include the alkene signals (from the COD ligand), the aminophenolic C–H signals, and the protic H signals from **L1** (O–H_(ph)enol_ and N–H_(py)razole_ in **1**). For **1**, the alkene signals appear at 3.66, 3.46, 3.34, and 2.83 ppm. Those signals move downfield in response to oxygenation, appearing at 6.82, 5.97, 4.99, and 4.32 ppm for **2**. Perturbations of this magnitude are consistent with a low-valent Ir­(I) converting to an oxidized Ir­(III).^78^ The aminophenol C–H signals are less responsive to oxygenation, moving from 7.08 and 6.79 ppm for **1** to 6.35 and 6.33 ppm for **2**. The acidic O–H_(ph)enol_ and N–H_(py)razole_ signals were observed at 6.34 and 9.56 ppm in **1**, but they were not detectable by ^1^H NMR after oxygenation, even when the reaction was performed at −40 °C. However, because **2** features two ν_X–H_ (X = N or O) bands by IR spectroscopy (3686 and 3601 cm^–1^), we infer that those protons are still on the complex.^79,80^

**Scheme 2:**
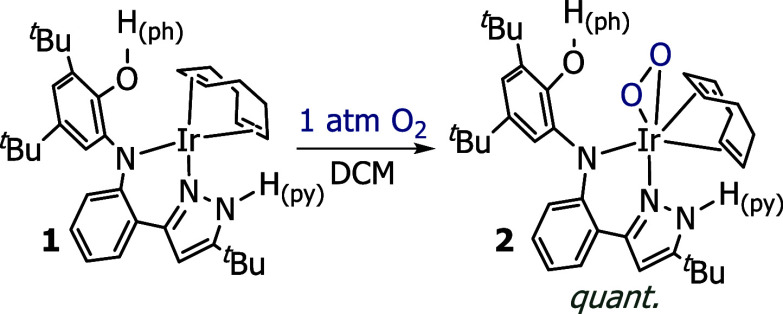
Oxygenation of **1** to Form **2.**

The oxygenation of **1** to form **2** was also studied by UV–vis spectroscopy at room temperature. Two isosbestic points were observed at 301 and 398 nm, consistent with clean one-to-one conversion. Pseudo-first-order conditions were obtained by adding excess O_2_ (in the form of a saturated CH_2_Cl_2_ solution)^81^ to a limiting quantity of **1** in CH_2_Cl_2_. Absorption values at 347 nm were monitored and plotted as a function of time; the resultant kinetic trace is consistent with a first-order dependence on **1** (Figure 3, pseudo-first-order rate constant *k*_obs_ = 1.20(2) × 10^–2^ s^–1^). Virtually identical values for *k*_obs_ were obtained at other wavelengths.^82^ A series of *k*_obs_ values were measured at different concentrations of O_2_. The resultant plot of *k*_obs_ vs [O_2_] is linear with an intercept of zero—consistent with a first-order dependence on O_2_. Our experiments yield the experimental rate law −d­[**1**]/dt = *k*_2_[**1**]­[O_2_], with *k*_2_ as 3.3(3) × 10^–6^ M^–1^ s^–1^. For comparison, Vaska’s complex IrCl­(CO)­(PPh_3_)_2_ oxygenates with an analogous second-order rate constant *k*_2_ = 3.4 × 10^–2^ M^–1^ s^–1^ in C_6_D_6_.^83^ This perturbation corresponds to approximately 5.4 kcal mol^–1^ higher activation free energy for **1**, which can be rationalized based on a weaker overall ligand field.^84^

**Figure 3:**
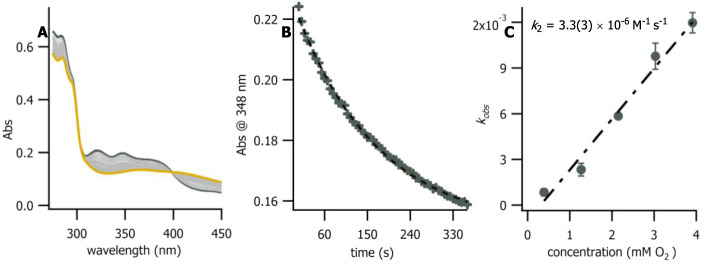
(Left) UV–vis spectrum of **1** (green) converting to **2** (gold) after the addition of excess O_2_. (Center) Representative time course for the pseudo-first-order consumption of **1** (0.04 mM) with excess O_2_ (3.03 mM). (Right) Pseudo-first-order rate constant *k*_obs_ vs [O_2_]; all measurements were made via UV–vis at 298 K in CH_2_Cl_2_.

The reactivity of **2** is also consistent with it being dioxygenated. For example, **2** reacts with (2,2,6,6-tetramethylpiperidin-1-yl)­oxyl (TEMPO•) in CD_3_CN, resulting in the precipitation of dark red crystals (**3** in Scheme 3, 63% yield). The ^1^H NMR spectrum of the mother liquor indicated that TEMPO–H was generated as a byproduct, so **3** should have one fewer hydrogen atom than **2**. This formulation was corroborated by the X-ray structure of **3**, which also reveals that the complex rearranged to a metallatrioxolane (Figure 4). As has been true for other crystallographically characterized metallatrioxolanes (e.g., **II**_**Fied**._ or **II**_**Bhat**._), the extradiol C–C of **3** looks positioned to attack the O–O σ*. A more thorough structural comparison is in the discussion section.

**Scheme 3:**
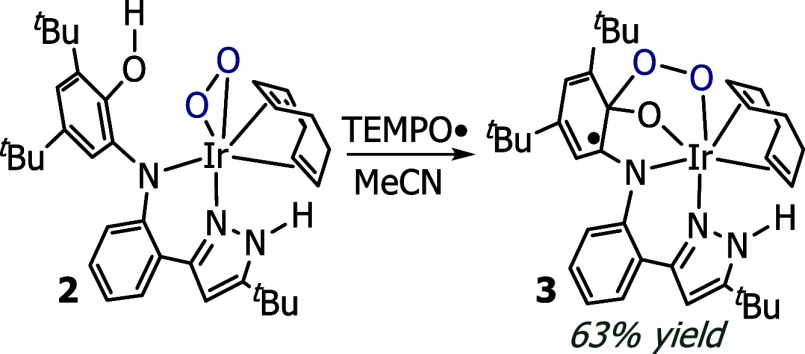
Reaction of **2** with TEMPO• to Form **3**

**Figure 4:**
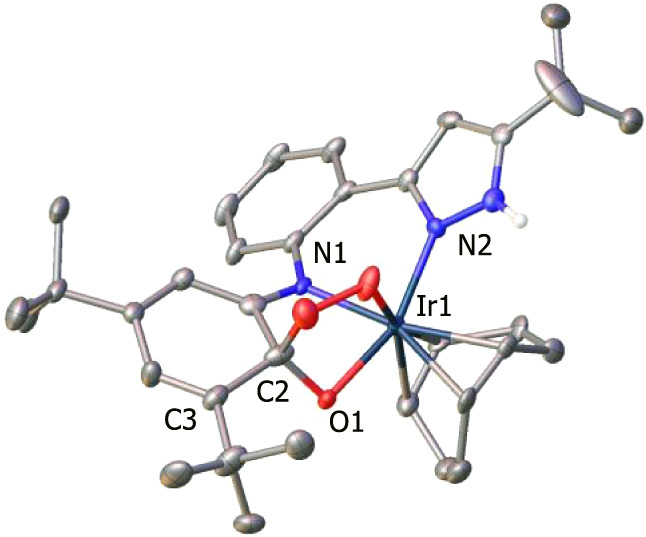
Thermal ellipsoid plot of **3** presented at the 50% probability level. Carbon-bound hydrogen atoms are omitted for clarity.

Because **3** is generated from a one-electron adjustment of **2** (net loss of H• from a d^6^/amidophenol), it presumably has one more electron than the existing synthetic metallatrioxolanes. Most previous cases were synthesized via the oxygenation of a d^6^/catecholate, resulting in a d^6^/quinone-like metallatrioxolane. The metallatrioxolane **3**, on the other hand, is an open-shelled species that falls somewhere between the extremes of d^7^/quinone-like and d^6^/semiquinone-like.^85^ The latter description is more consistent with the EPR spectrum of **3**. A near-axial signal was observed at 70 K from a frozen 1 mM solution of **3** in 50:50 MeCN:THF (Figure 5), with principal g-values of g_1_ = 2.020, g_2_ = 2.006, and g_3_ = 1.944. The small g-anisotropy, with g-values close to *g*_e_ (2.0023), is similar to reported spectra for the oxidation products of Ir­(III)-catecholates (similarly assigned as Ir­(III)-semiquinones).^86,87^ We disfavor the Ir­(II)/quinone-like assignment because the strong spin–orbit coupling expected for third-row metals is expected to result in much larger g-anisotropy; Ir­(II) complexes with less obviously “redox-active ligands” exhibit principal g-values as high as 3.0.^88–91^ In addition, we performed DFT calculations on **3** using crystallographic data as an initial guess. The resultant prediction placed the majority of the unpaired spin on the ligand, as would be expected for an Ir­(III)-semiquinone-like assignment (TPSSh functional, def2-TZVP basis set^92^ with def2/J auxiliary basis set for RIJ-COSX RI approximation;^93^ see the SI and Discussion section).

**Figure 5:**
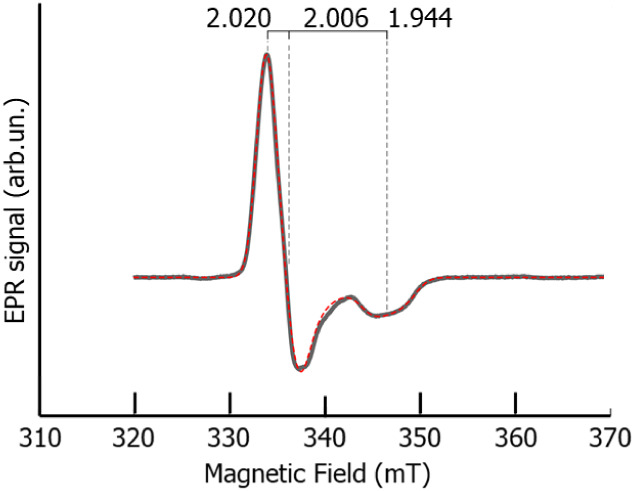
EPR spectra of **3** measured at 70 K. Gray traces are experimental data, and dashed red lines are simulations. Principal g-values from matching simulation/experimental data are depicted. Simulation parameters for **3**: g_1,2,3_ = [2.020, 2.006, 1.944], g_1,2,3_-strain = [0, 0, 0.0114], A_1,2,3_(^14^N) = [6.4, 23, 51] MHz, line width = 2.2 mT.

The high-field component of the EPR signal of **3** exhibits nonuniform broadening, likely due to^14^N (*I =* 1) hyperfine coupling(s). The simulation reflects this possibility, with the three principal ^14^N HF coupling constants estimated to be A_1_ = 6 MHz, A_2_ = 23 MHz, and A_3_ = 51 MHz.^89^

In addition to metallatrioxolane **3** (which models **II**_**extra**_), peroxo **2** can be converted to models of **III**_**extra**_. Red-amber solutions of **2** in CH_2_Cl_2_ were observed to darken and decompose in ambient light.^94^ When samples were intentionally photolyzed at 595 or 400 nm, the samples similarly darkened, resulting in uninterpretable ^1^H NMR spectra. However, samples irradiated with a blue LED (emission centered at 467 nm) became yellow (Scheme 4).

**Scheme 4:**
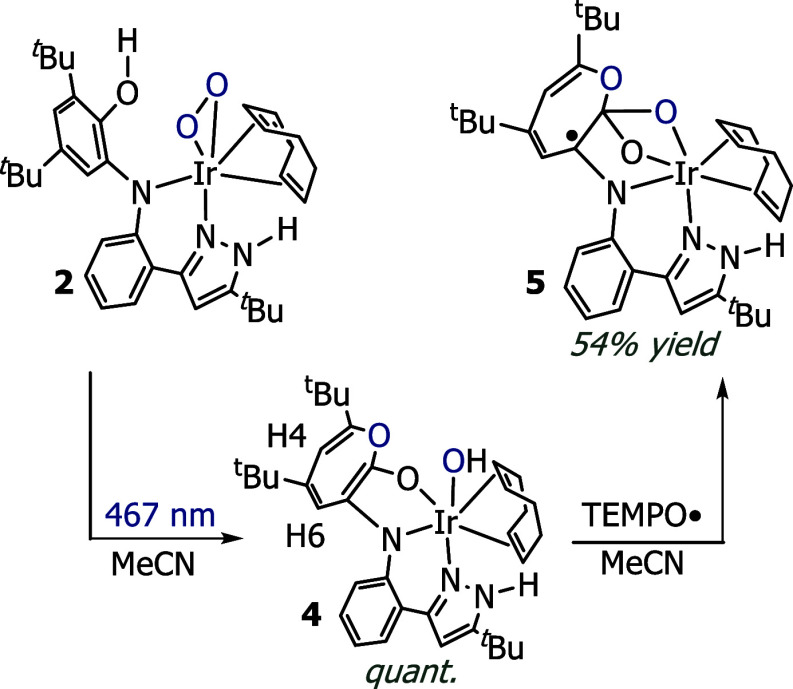
Photolysis of **2** and Subsequent Reaction with TEMPO•

The extent of the reaction was monitored by UV–vis, where two kinetic regimes were observed (Figure 6). An initial rapid change in the absorption spectra occurred within 5 s of photolysis, which proved too fast to monitor. Continued photolysis resulted in a second, slower set of changes to the absorption spectra, with isosbestic points at 328 and 447 nm. The overall profile of the UV–vis is consistent with an A → B → C reaction mechanism, where the first step is fast and the second step is slow (with **2** being “A”). The isosbestic points presumably manifest when the absorption from **2** becomes negligible.

**Figure 6:**
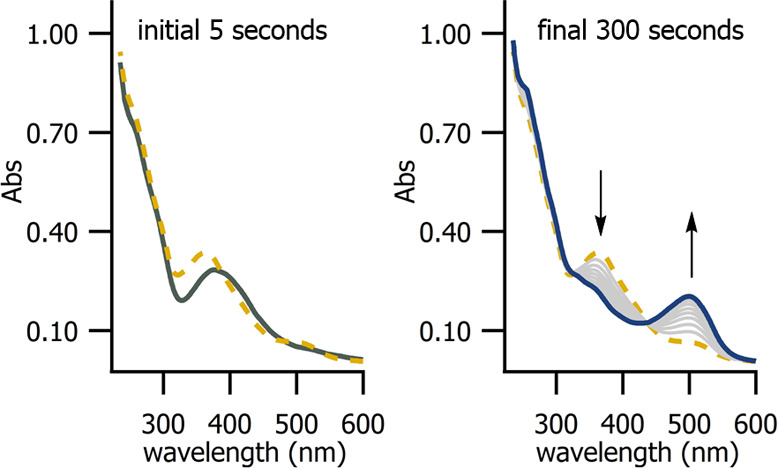
Monitoring photolytic (irradiation at 467 nm) conversion of **2** in CH_2_Cl_2_ by UV–vis: (Left) Initial 5 s, conversion of **2** (green) to an intermediate (gold); (Right) Continued photolysis over 300 s, further conversion to **4** (blue).

When the photoreaction of **2** was monitored by ^1^H NMR spectroscopy (CD_2_Cl_2_, room temperature), the signals corresponding to **2** declined, and a single set of signals corresponding to **4** grew in. The yellow color of the final solution is more consistent with assigning **4** as “C” from the UV–vis experiment (the optical spectra suggest “A” and “B” are similarly colored); i.e., the intermediate cannot be detected at these concentrations. The two aminophenolic C–H resonances (for **4**) appear at 6.52 and 5.78 ppm. Both the absolute chemical shifts and the 0.74 ppm difference are significant. We could not find comparable resonances among any previously reported aminophenolate, (imino)­semiquinone, or (imino)­quinone (including **1** and **2**, Table 1). However, these chemical shifts are what would be expected from an extradiol insertion, with H(6) comparable to an α,β-unsaturated imine and H(4) comparable to an enol ether.^95^ This interpretation is further reinforced by a diagnostic ^13^C NMR signal at 180.29 ppm, which is consistent with a lactone carbon.^96,97^ These spectral data were used in conjunction with the structure of a downstream product (**5**, *vide infra)* to assign **4** as the α-iminolactone-bound Ir–OH (Scheme 3).

**Table 1:** Selected ^1^H NMR Resonances of **1**, **2**, and **4** in CD_2_Cl_2_

	**O—H (ppm)**	**N—H (ppm)**	**phenol C—H (ppm)**	**COD C—H (ppm)**	
1	6.34	9.56	6.79	2.83	3.34
			7.08	3.46	3.66
2	–	–	6.33	4.32	4.99
			6.35	5.97	6.82
4	–	–	5.78	3.92	5.02
			6.52	6.01	6.53

All attempts to crystallize **4** resulted in a derivative complex, **5**, which is deficient in a hydrogen atom (analogous to the relationship of **2** to **3**). The hydrogen atom abstraction could be triggered with TEMPO•, resulting in the immediate precipitation of a yellow solid that was isolated and characterized. The structure of **5** verifies that the aminophenol has undergone extradiol insertion, where one of the oxygen atoms of O_2_ has inserted into the extradiol position of the ring (Figure 7).

**Figure 7:**
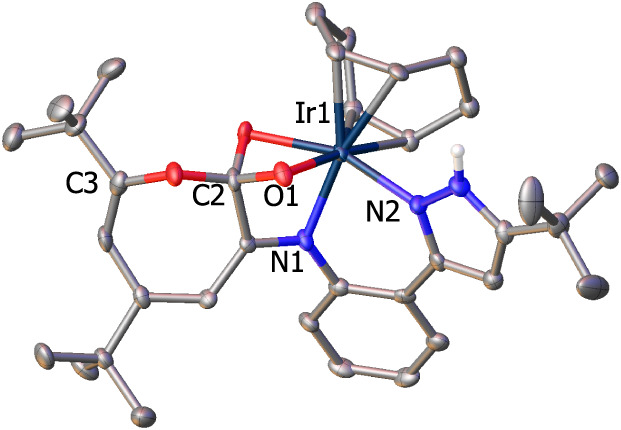
Thermal ellipsoid plots of **5** presented at the 50% probability level. Carbon-bound hydrogen atoms are omitted for clarity.

A 1 mM sample of **5** in 50/50 MeCN/THF resulted in an EPR spectrum (measured at 70 K) with a complex signal that, according to our simulations, is best described as being composed of two species (Figure 8). The major signal (70% of the signal) is very similar to that of **3**,^98^ with principal g-values of 2.017, 2.003, and 1.938. A second, minor species (30% of the signal) results in a more anisotropic signal, with g-values of 2.037, 2.024, and 1.888, indicating a small but comparatively increased spin population on Ir. This minor spectral component also exhibits greater line broadening. Because both signals exhibit small overall g-anisotropy that would be atypical of Ir­(II) we have assigned **5** as Ir­(III)/semiquinone-like, analogously to **3**. DFT calculations using the crystallographic data for **5** as an initial guess also suggest that most of the spin density is on the ligand. The two EPR-active species may reflect the reactive nature of **5**, which (for example) reacts with solvents like MeCN; see also the Discussion section.^99^

**Figure 8:**
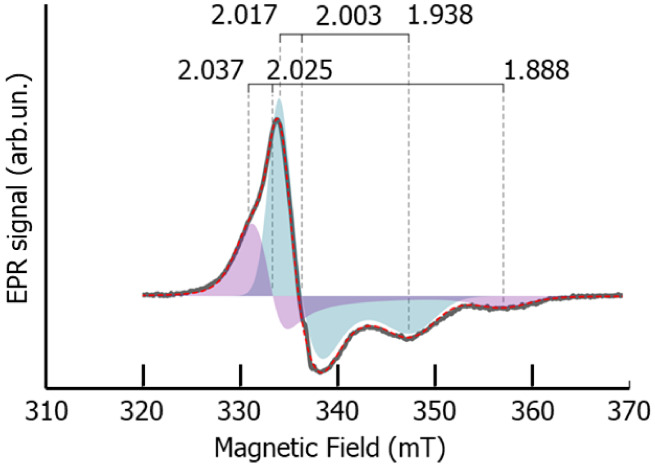
EPR spectra of **5** measured at 70 K. Gray traces are experimental data, and dashed red lines are simulations. For **5**, the simulation includes two species (teal and magenta, respectively). Principal g-values from matching simulation/experimental data are depicted. Simulation parameters for **5**: Major species (∼70%) – g_1,2,3_ = [2.017, 2.003, 1.938], g_1,2,3_-strain = [0.004, 0.021, 0.029], line width = 2.3 mT. Minor species (∼30%) – g_1,2,3_ = [2.037, 2.025, 1.888], g_1,2,3_-strain = [0.019, 0.012, 0.028], line width = 1.9 mT.

## Discussion and Conclusions

Both the metallatrioxolane **3** and the ring expansion product **5** have unusual electronic configurations.

Regardless of whether **3** is assigned as “d^6^/semiquinone-like” (our favored assignment from EPR data) or “d^7^/quinone-like,” the metal/ligand complex has one more electron than other crystallographically characterized metallatrioxolanes (all of which, to our knowledge, were assigned as “d^6^/quinone-like”). Despite this one-electron difference, the structure of **3** is overall similar to other crystallographically characterized “extradiol” metallatrioxolanes.^30,31,100^ For example, because O_2_ now bridges Ir­(I) and C(2), those atoms are now bridgehead positions of a bicyclo[2.2.1] framework. The three “fins” of the bicycle—defined as the mean plane of the four-atom, four-atom, and three-atom bridges—are separated by 124.8(1)°, 130.3(1)°, and 105.2(1)° (Figure 9). These angular metrics fall within the range observed in **II**_**Fied.**_ and **II**_**Bhat**_.

Similarly, there are minor metrical differences in the alkylperoxide O–O and extradiol C–C of **3**. The extradiol C(2)–C(3) bond is 1.56(1) Å, ∼0.03 Å longer than the next longest case, **II**_**Fied.**_ The C(2)–O(3) bond is 1.43(1) Å, 0.03 Å shorter than the next shortest case, also **II**_**Fied.**_ The vectors comprised of O(3)–O(2) and extradiol C(2)–C(3) bonds are 8.49° perturbed from linearity, the next most perturbed case being **II**_**Fied.**_ at 7.52°. These metrical comparisons suggest that, among crystallographically characterized metallatrioxolanes, **3** is marginally closer to a Baeyer–Villiger-like transition state.^101,102^

The small structural perturbations may suggest that the extra electron of **3** is highly delocalized around the ligation sphere. A potentially diagnostic measurement is the puckering of the central aryl ring, where the six carbons are, on average, 0.03(1) Å displaced from the mean plane (ring **b** pucker, Figure 9). For comparison, the corresponding average displacement in the diamagnetic **1** is 0.00(1). DFT-computed Mulliken spin populations also suggest that some spin density is present on the “B” ring (see SI section DFT of 3 and 5).

**Figure 9:**
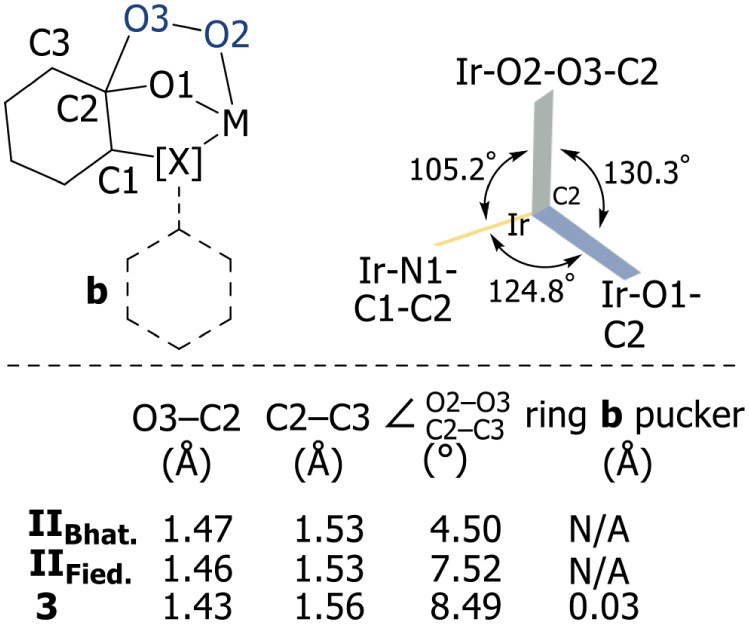
(Top left) Schematic of a generalized trioxolane. (Top right) “Fins” of the bicyclo[2.2.1] framework with Ir­(I) and C(2) as bridgehead positions. (Bottom) Table comparing multiple metallatrioxolanes.

We considered the hypothesis that the small observed structural perturbations reflect a “d^7^/quinone-like” assignment, especially using a metrical oxidation state assignment reported by Brown.^103^ While this analysis results in a “quinone-like” assignment, we note that the method was developed to analyze metal-amidophenolates and metal-catecholates. The ligand of **3** may not conform to the established boundary conditions, as this case also features a bridging O_2_ ligand, as well as additional rings **b** and **c**.

Finally, the “d^6^/semiquinone-like” assignment is more consistent with expected “up-down” trends when comparing light vs heavy metal congeners. A comparable example of valence isomerism is high-spin “d^4^/oxidized porphyrin (porphyrin cation radical)” vs “d^3^/porphyrin” (Figure 10). The former configuration is favored for (X)­(Por^+^)­Fe­(IV)=O, while the latter is favored for the heavier congener (X)­(Por)­Ru­(V)=O (X = thiolate; Por = porphyrin).^104,105^ The differing electronic structures were attributed to larger ligand field splitting for Ru over Fe, which is rationalized by (a) general ligand field splitting trends for 5d > 4d > 3d metals and (b) the more radially diffuse d-orbitals strengthening metal–ligand bonding. These factors make it increasingly difficult to obtain the high-spin “d^4^/oxidized porphyrin” configuration when moving down from the first-row metal. Applying the same logic to **3** similarly rationalizes the “d^6^/semiquinone-like” assignment.^106^

**Figure 10:**
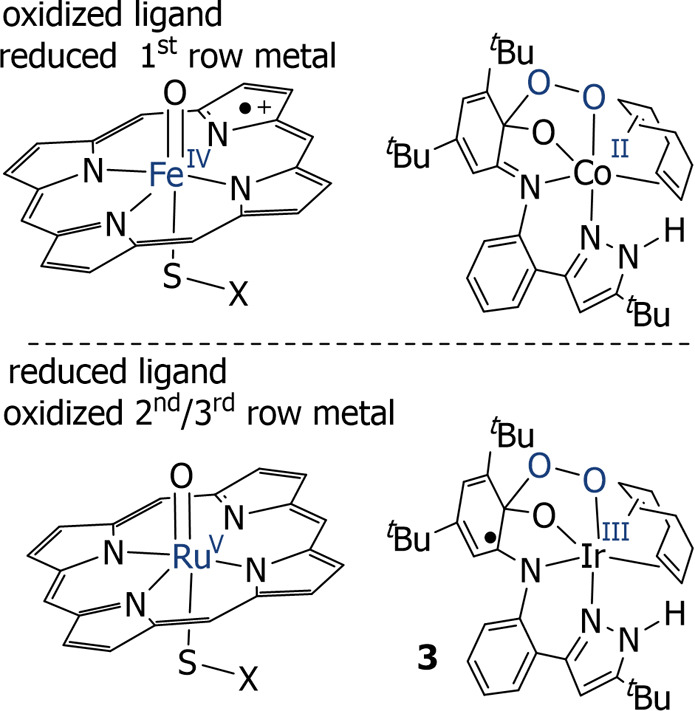
Comparing 1st row and 2nd/3rd row complexes: (Left) Fe and Ru porphyrin complexes^104^ (Right) hypothetical Co-containing trioxolane and **3.**

Similarly, the paramagnetic **5** has one more electron than typically drawn intermediates for the ring-cleaving dioxygenases. As was the case for **3**, “d^6^/semiquinone-like” and “d^7^/quinone-like” represent two extreme assignments of electronic structure. For **5**, there are fewer opportunities for good electronic or X-ray structural comparisons because 7-membered ring states tend to be transient, e.g., undergoing ring-opening^20,95^ and/or subsequent rearrangements.^107^ Perhaps the singular exception is the recently observed Fe case by Popescu and Fiedler,^108^ though that oxepine was not amenable to X-ray crystallography. In addition, the structure of **5** features an orthoester bound in a dianionic κ^2^-coordination mode, a feature that has no crystallographic analogy in the Cambridge Structural Database (to our knowledge). This observed structure can be rationalized as an oxyl (e.g., arising from abstraction of H• from Ir–OH **4**) attacking the lactone carbon, converting Ir­(I) and C(2) into bridgeheads of a bicyclo[2.1.1] scaffold. These types of redox-neutral rearrangements may be underconsidered in prior discussions of mechanism.

The absence of good structural comparisons requires assigning **5** predominantly based on its EPR spectra, the prediction from DFT, and by extending our outlined rationale for **3**. So, the “d^6^/semiquinone-like” assignment seems most prudent. We also note that the Mulliken spin population analysis for **5** suggests that the spin is even more delocalized than was the case for **3** (SI: DFT of 3 and 5), another potential advantage of using the unusually large aminophenol ligand **L1**.

With respect to the extradiol ring-cleaving dioxygenases, our observations may be most relevant to understanding metallocofactor promiscuity. The canonical mechanism of extradiol insertion was defined for Fe­(II)-containing metalloenzymes, though Co­(II)^106,107^ and Mn­(II) variants are now known to be equally active. Because Co­(II) features one more electron than Fe­(II), it may be that the established intermediates in the consensus mechanism also have one-electron-reduced congeners, analogous to **3** and **5**. The “up-down” arguments we have outlined suggest that the corresponding Co adducts would be valence isomers, i.e., being “d^7^-quinone-like” instead of “d^6^-semiquinone-like.” We speculate that these subtly different electronic states will prove important for rationalizing mechanistic divergence in both synthetic and metalloenzymatic systems.^74,109,110^

The strategy we used to generate these dioxygenated intermediates—reacting d^8^/amidophenol **1** with O_2_—starts with an unusually large number of electrons in the metal/substrate “pool.” For example, **I**_**Fied**_ is a d^7^/amidophenolate, while **I**_**Bhat**_ is representative of many d^7^/semiquinones or d^6^/catecholate valence isomers. Complexes like **I**_**Bhat**_ lack the requisite number of electrons to generate analogues of **3** or **5**. The d^7^/amidophenolate **I**_**Fied**_ has the necessary number of electrons, but reactions with O_2_ apply downward pressure to the electron count. For example, the oxygenation of **I**_**Fied**_ first results in oxidation to the d^7^/(imino)­semiquinone—which no longer has the requisite number of electrons; a second equivalent of O_2_ then assembles the metallatrioxolane. The unusual synthetic strategy we used, starting from an over-reduced state, may have made it possible to access more “reduced” dioxygenated intermediates.

Because virtually all oxidations with O_2_ are exergonic, dioxygenated transition metal complexes can be “thermodynamically charged” to undergo many subsequent reactions. For example, in this work, both paramagnetic complexes **3** and **5** were generated by reaction with the moderate hydrogen atom acceptor TEMPO• (BDFE_TEMPO–H_: 66 kcal mol^–1^).^111^ These reactions point to a weakening of the O–H bond in precursor complexes **2** and **4**, thermodynamic perturbations that—at least in part—reflect compensatory contributions from rearranging the O_2_-derived ligands.^112^ That having been said, dioxygenated complexes can lose a portion of the potential energy stored in O_2_ if the dioxygenation is too exergonic. Vaska-like Ir complexes are an unusual opportunity because their dioxygenations are often reversible. Productive rearrangements to dioxygenated intermediates that have historically been associated with specific metalloenzymes,^113^ especially bridging alkyl peroxides, may be a generalizable strategy for obtaining selective oxygenation outcomes.^26,27,114–116^ However, subtle stereoelectronic factors may govern the assembly and rearrangement for every metal/substrate combination, analogous to the historical challenges modeling the extradiol insertion reaction.

To summarize, incorporating the ring-cleaving dioxygenase substrate into a larger, multidentate ligand facilitated access to a substrate-bound state within a weaker ligand field environment (compared to earlier synthetic models) (Figure 1).^29,30^ While the initially observed κ^2^-binding mode of **1** does not have an immediate enzymatic analogy,^74,117^ the oxygenated **2** rearranges to **3, 4,** and **5**—complexes that model ring-cleaving dioxygenase intermediates. The higher complexity of **L1**, e.g., the extra point of chelation, results in additional thermodynamic influence that reinforces the association of a substrate/product to the metal. This chelation also introduces new conformational restrictions. These two factors together may elongate the lifetimes of what might otherwise be transient intermediates.

## Supplementary Material



## Notes

Contents include synthetic procedures procedures for kinetics characterization data­(PDF)
