# Supplementary material for: Mimicking Extradiol Dioxygenase Reactivity on Iridium
Source: J Am Chem Soc. 2026 Jun 10;148(27):28122–32. doi: 10.1021/jacs.5c23353 (PMC13383722; doi:10.1021/jacs.5c23353)
Supplement: Supplementary file 1 [file ja5c23353_si_001.pdf]

Mimicking Extradiol Dioxygenase Reactivity on Ir

Alexander G. Arnette, Anant Kumar Jain, Alexey Silakov, Karen I. Goldberg, Jonathan L. Kuo

Contents

General Procedures ... 3

Synthetic Procedures ... 3

Synthesis of 1-(2-aminophenyl)-4,4-dimethylpent-2-yn-1-one ... 3

Synthesis of 1-(2-((3,5-di-tert-butyl-2-hydroxyphenyl)amino)phenyl)-4,4-dimethylpent-2-yn-1-one ... 6

Synthesis of 2,4-di-tert-butyl-6-((2-(5-(tert-butyl)-1H-pyrazol-3-yl)phenyl)amino)phenol 9

Synthesis of **1** ... 12

O_2 Gas Addition Procedure ... 15

Synthesis of 2 ... 17

Synthesis of 3 ... 21

Relationship between [**2** and **3**]. ... 22

Synthesis of 4 ... 24

5 ... 28

Relationship between [**4** and **5**]. ... 29

O_2 Gas Addition Procedure ... 31

Kinetics ... 32

Photolysis Procedure ... 44

Solution State IR Behavior of 1 and 2 ... 46

^{18}O-**2** and ^{16}O-**2** IR ... 47

EPR of **3** and **5** 49
DFT Calculations for **3** and **5** 49
Crystallographic Data 64
1 64
3 67
5 71
Works Cited 74

General Procedures

All manipulations were performed under N_2 using standard Schlenk line or inert atmosphere glovebox techniques. All glassware was flame-dried or oven-dried before use. NMR spectra were collected with a Bruker 400 or 500 MHz spectrometer. Deuterated solvents were purchased from Cambridge Isotope Laboratories, degassed by three successive freeze pump thaw cycles and stored over molecular sieves in Schlenk tubes inside of an inert atmosphere glovebox. CH_3CN, Et_2O, THF, DCM, and pentane were dried by passage through activated alumina and molecular sieve columns under N_2 using a Grubbs type solvent purification system by Glass Contour. IR spectra were collected using a Bruker ALPHA II spectrometer via transmission in a Perkin-Elmer IR liquid cell (CaF_2 semi-demountable cell, 1 mm fixed pathlength) or attenuated total reflectance (ATR) with a diamond ATR crystal. UV-vis spectra were collected in quartz cuvettes (Spectrocell Inc.) using an Agilent Cary 60 UV-vis spectrometer. X-ray data were measured on a Bruker Kappa APEXII Duo system equipped with a Incoatec Microfocus IμS (Cu Kα, λ = 1.54178 Å) and a multi-layer mirror monochromator at the X-ray facility of The Pennsylvania State University. Elemental analysis was performed under air-free conditions at the University of Rochester. $[Ir(COD)(CH_3CN)_2](BF_4)$ were synthesized according to published procedures and stored at -25 ^{0}C in an inert atmosphere glovebox. All other reagents were commercially available and used without further purification.

CAUTION! Handling closed systems under pressure can be dangerous, so careful consideration of PPE is required. Pressurized vessels should be contained in secondary containment to contain glass shrapnel in a shattering event. Pressurized J. Young tubes were always wrapped in tin foil and then transported in thick plastic containers. Oxygen is oxidizing and flammable. Oxygen should not be mixed with reducing gases. Oxygen which is pulled off using a Schlenk line which has a condenser set up may condense into liquid oxygen.

Synthetic Procedures

Synthesis of 1-(2-aminophenyl)-4,4-dimethylpent-2-yn-1-one

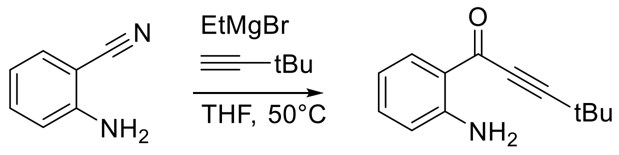

Scheme S1. Synthesis of 1-(2-aminophenyl)-4,4-dimethylpent-2-yn-1-one

3,3-dimethyl-1-butyne (3.12 mL, 25.4 mmol) was dissolved in 40 mL of dry THF under and N_2 atmosphere. Ethylmagnesium bromide (8.88 mL, 25.6 mmol) was added dropwise with vigorous stirring over a period of 3 minutes. The solution was heated to 50 °C for 2 hours and became a clear gray in color. 2-aminobenzonitrile (1.50 g, 12.6 mmol) was dissolved in minimal THF and added to the reaction mixture dropwise over a period of 5 minutes. The solution immediately turned a bright yellow. The reaction mixture was heated to 50 °C until TLC (silica, 20% EtOAc/hexanes) indicated full consumption of the 2-aminobenzonitrile, approximately 16 hours. The reaction mixture was now a deep red. The reaction mixture was cooled to 0 °C and to it was added of 1 M H_2SO_4 (1 equivalent, 12.4 mL). The solution was stirred for 10 minutes before 0.5 equivalents more of 1 M H_2SO_4 was added. The now biphasic reaction mixture was stirred vigorously at room temperature for 24 hours. The reaction mixture was neutralized with saturated $NaHCO_3$ until pH paper indicated a pH of 7. The mixture was transferred to a separatory funnel and the reaction vessel was washed with EtOAc (3x), which was added to the same separatory funnel. The layers were separated, and the aqueous layer was extracted with H_2O (3x). The organic layers were combined and washed with brine (3x). The combined organic layers were dried over $MgSO_4$ and evaporated to yield a yellow crude residue. The crude was purified via silica chromatography (using a 10% - 30% EtOAc/hexanes gradient) to yield a yellow solid. The yield for this product is 41%.[1]

^{1}H NMR (400 MHz, $CDCl_3$) δ 8.04 (dd, *J* = 8.2, 1.6 Hz, 1H), 7.33 – 7.26 (m, 1H), 6.68 (ddd, *J* = 8.2, 7.1, 1.3 Hz, 1H), 6.62 (dd, *J* = 8.4, 1.3 Hz, 1H), 6.31 (s, 2H), 1.37 (s, 10H).

^{13}C{^{1}H} NMR (101 MHz, $CDCl_3$) δ 180.23, 151.04, 135.10, 134.72, 119.12, 116.81, 116.11, 103.05, 78.46, 30.39, 28.12.

HRMS (ESI-TOF) m/z: $[M + H]^+$ Calcd for $C_{13}H_{17}NO$: 202.1226. Found: 202.1227

FTIR (neat) cm^{-1}: 3449 (br), 3330 (br), 2216 (s), 1617 (s), 1582 (s), 1258 (s), 1230 (s), 745 (s)

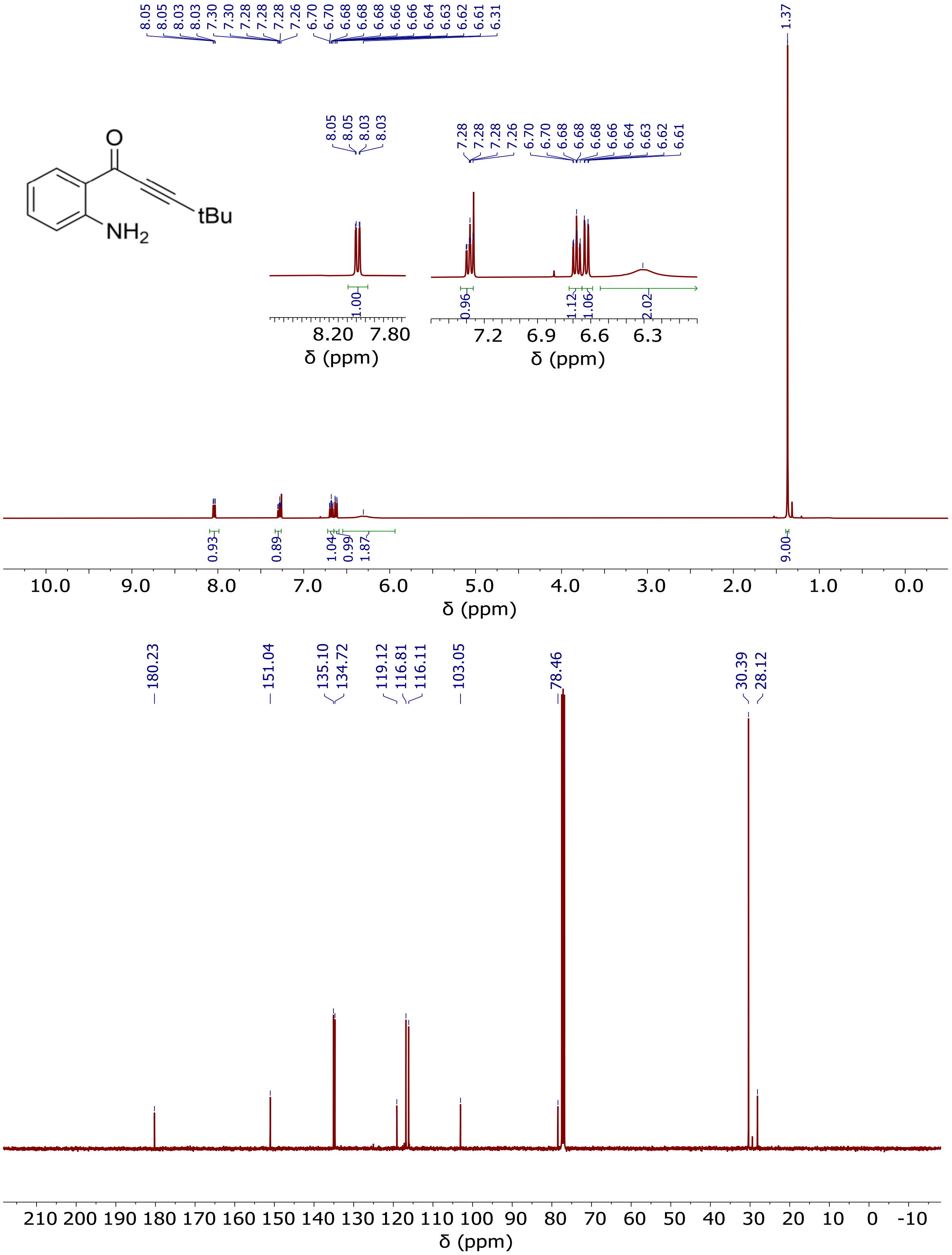

Figure S1. ^{1}H (top) with enlarged aryl region and ^{13}C {^{1}H} (bottom) NMR of **1-(2-aminophenyl)-4,4-dimethylpent-2-yn-1-one** in $CDCl_3$.

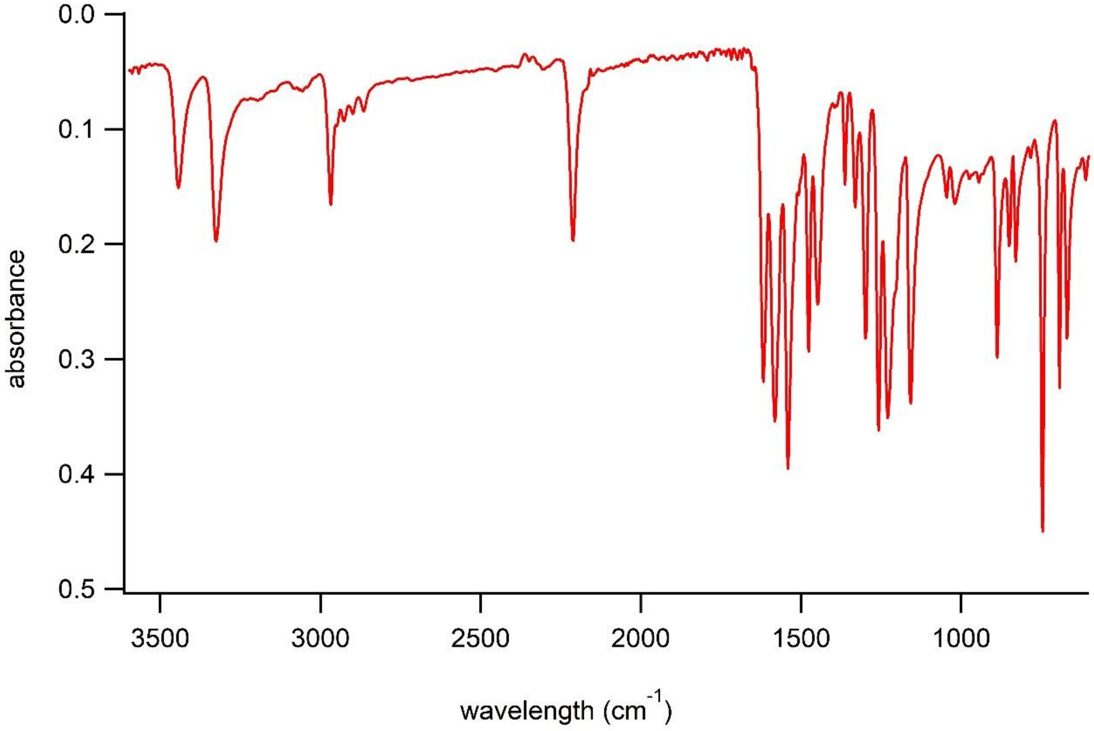

Figure S2. FTIR of **1-(2-aminophenyl)-4,4-dimethylpent-2-yn-1-one** (neat).

Synthesis of 1-(2-((3,5-di-tert-butyl-2-hydroxyphenyl)amino)phenyl)-4,4-dimethylpent-2-yn-1-one

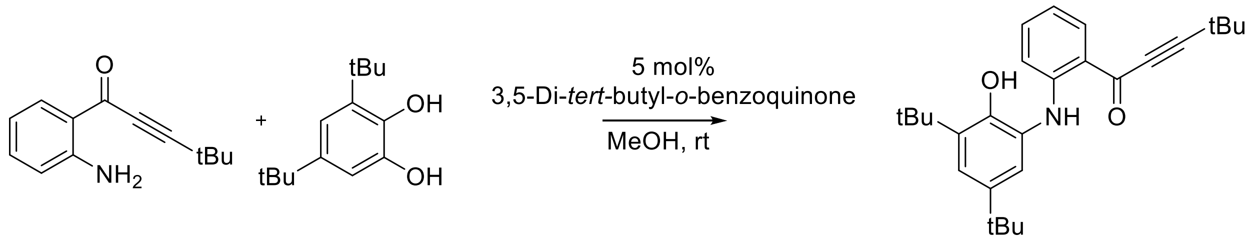

Scheme S2. Synthesis of 1-(2-((3,5-di-tert-butyl-2-hydroxyphenyl)amino)phenyl)-4,4-dimethylpent-2-yn-1-one

Two preparations are reported for converting the amino-ynone to the aminophenolato-ynone.

Preparation 1: 1-(2-aminophenyl)-4,4-dimethylpent-2-yn-1-one (570 mg, 2.83 mmol) and 3,5-Di-tertbutylcatechol (629 mg, 2.83 mmol) were dissolved in 8 mL of MeOH and stirred rapidly open to air. To the reaction mixture was added 3,5-Di-tertbutyl-*o*-benzoquinone (approximately 5 mol %, 30 mg) dissolved in 6 mL of MeOH over the course of 24 hours using

a InfusionONE Single Channel Syringe Pump.* The solution was stirred for an additional 24 hours, and the reaction mixture darkened from a deep yellow to a dark brown. To the bottom of the reaction vessel was added a stream of air from a needle, and the reaction was stirred for an additional 24 hours. As the reaction mixture evaporated to dryness, minimal MeOH (approximately 1 mL) was added and stirring was continued. This process was repeated until TLC (silica, 5% EtOAc/hexanes) indicated all 3,5-Di-tertbutylcatechol was consumed, approximately 14 days.

Preparation 2: Alternatively, the reaction could be set up using the same conditions as above but without adding the 3,5-Di-tertbutyl-*o*-benzoquinone by syringe pump. The reaction was stirred for 24 hours with an added stream of air.* Approximately every 24 hours, additional methanol was added to prevent the mixture from drying out. This process was repeated for approximately 30 days, or until TLC (silica, 5% EtOAc/hexanes) indicated all 3,5-Di-tertbutylcatechol was consumed. The crude mixture was evaporated and purified via silica chromatography (5% - 10% EtOAc/hexanes gradient) to yield orange-brown crystals. The yield for this product is 73%.

*This reaction demonstrates characteristics of being a radical chain, where the chain carrying species is an oxidized version of the catechol substrate. These procedures seemingly improve the reproducibility generating the chain carrying species.

^{1}H NMR (400 MHz, Acetone-d_6) δ 9.89 (s, 1H), 8.18 (dd, *J* = 8.1, 1.7 Hz, 1H), 7.78 (s, 1H), 7.39 – 7.23 (m, 2H), 7.10 (d, *J* = 2.5 Hz, 1H), 6.76 (ddd, *J* = 8.0, 7.1, 1.1 Hz, 1H), 6.56 (d, *J* = 8.5 Hz, 1H), 1.45 (s, 9H), 1.40 (s, 9H), 1.29 (s, 9H).

^{13}C {^{1}H} NMR (101 MHz, Acetone-d_6) δ 180.66, 152.14, 150.54, 142.98, 137.51, 136.43, 135.67, 126.95, 123.55, 123.09, 120.75, 117.34, 114.66, 103.70, 79.29, 35.47, 30.64, 30.25, 30.06, 29.94, 26.03.

HRMS (ESI-TOF) m/z: $[M + H]^+$ Calcd for $C_{27}H_{35}NO_2$: 406.2741 Found: 406.2740

FTIR (neat) cm^{-1}: 3279 (br), 2963 (br), 2213 (s), 1615 (s), 1451 (s), 1265 (s), 1159 (s), 883 (s), 748 (s).

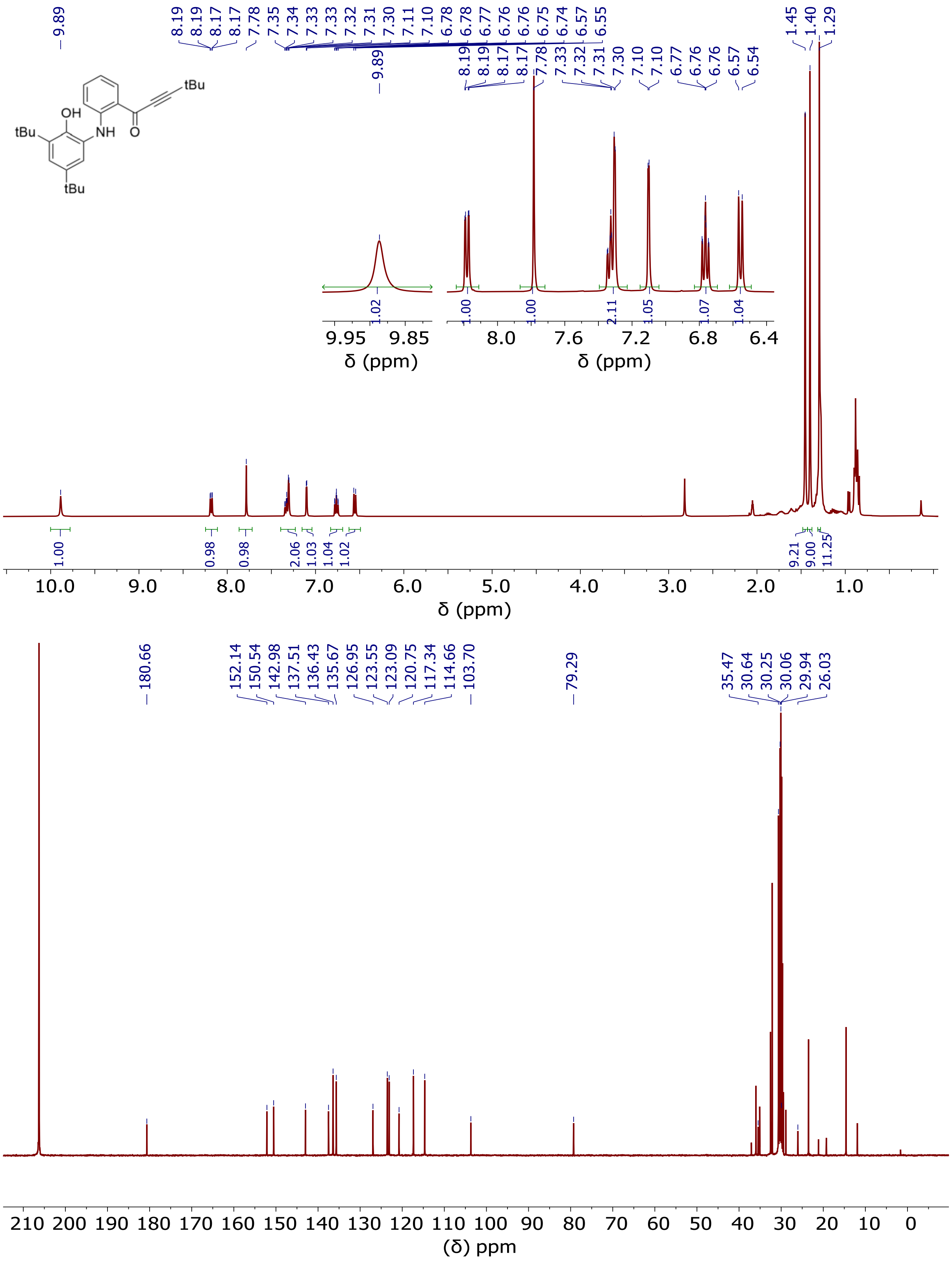

Figure S3. ^{1}H (top) with enlarged aryl region and $^{13}C\{^{1}H\}$ (bottom) NMR of **1-(2-((3,5-di-tert-butyl-2-hydroxyphenyl)amino)phenyl)-4,4-dimethylpent-2-yn-1-one** in acetone-d6.

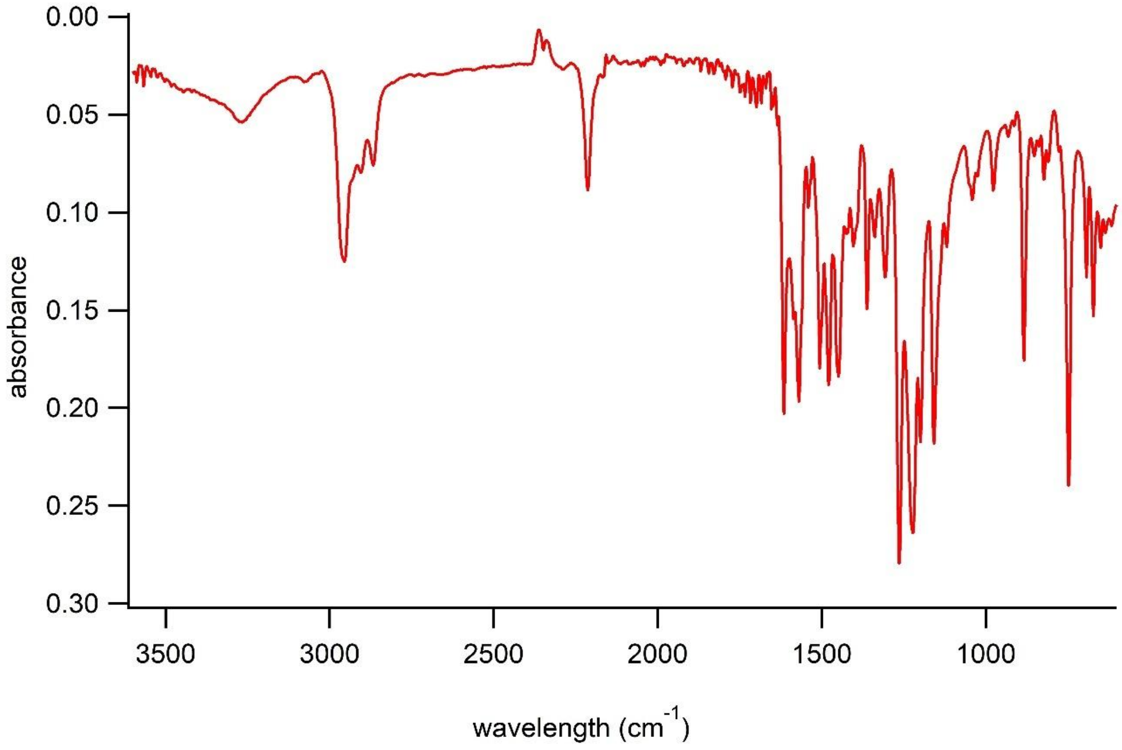

Figure S4. FTIR of **1-(2-((3,5-di-tert-butyl-2-hydroxyphenyl)amino)phenyl)-4,4-dimethylpent-2-yn-1-one** (neat).

Synthesis of 2,4-di-tert-butyl-6-((2-(5-(tert-butyl)-1H-pyrazol-3-yl)phenyl)amino)phenol

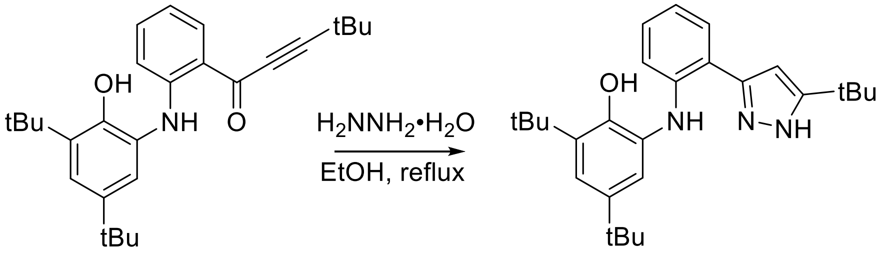

Scheme S3. Synthesis of 2,4-di-tert-butyl-6-((2-(5-(tert-butyl)-1H-pyrazol-3-yl)phenyl)amino)phenol

1-(2-((3,5-di-tert-butyl-2-hydroxyphenyl)amino)phenyl)-4,4-dimethylpent-2-yn-1-one (257 mg, 0.64 mmol) was dissolved in 7.5 mL of absolute EtOH. To the solution was added two equivalents of hydrazine hydrate (0.062 mL, 63.6 mg, 1.27 mmol), and the solution was brought to reflux. The reaction mixture was refluxed until TLC (silica, 5% EtOAc/hexanes) indicated consumption of the 1-(2-((3,5-di-tert-butyl-2-hydroxyphenyl)amino)phenyl)-4,4-

dimethylpent-2-yn-1-one, approximately 3 hours. The reaction mixture was concentrated to a tan oil and purified via silica chromatography (1% - 10% EtOAc/hexanes gradient) yielding 230 mg (87%) of tan to colorless crystals.

^{1}H NMR (400 MHz, Acetone-d_6) δ 12.01 (s, 1H), 9.23 (s, 1H), 7.69 (dd, *J* = 7.8, 1.7 Hz, 1H), 7.43 (s, 1H), 7.22 (d, *J* = 2.4 Hz, 1H), 7.11 (d, *J* = 2.5 Hz, 1H), 7.03 (ddd, *J* = 8.5, 7.1, 1.6 Hz, 1H), 6.76 (td, *J* = 7.4, 1.3 Hz, 1H), 6.68 – 6.59 (m, 2H), 1.48 (s, 9H), 1.44 (s, 9H), 1.28 (s, 9H).

^{13}C {^{1}H} NMR (101 MHz, Acetone-d_6) δ 180.66, 152.14, 150.54, 142.98, 137.51, 136.43, 135.67, 126.95, 123.55, 123.09, 120.75, 117.34, 114.66, 103.70, 79.29, 35.47, 30.64, 30.25, 30.06, 29.94, 26.03.

HRMS (ESI-TOF) m/z: $[M - H]^-$ Calcd for $C_{27}H_{37}N_3O$: 418.2864 Found: 418.2870T

FTIR (neat) cm^{-1}: 3402 (br), 3383 (br), 3316 (br), 2961 (s), 2904 (s), 2866 (s), 1582 (s), 1464 (s), 1223 (s), 957 (s), 932 (s), 881 (s), 816 (s) 797 (s).

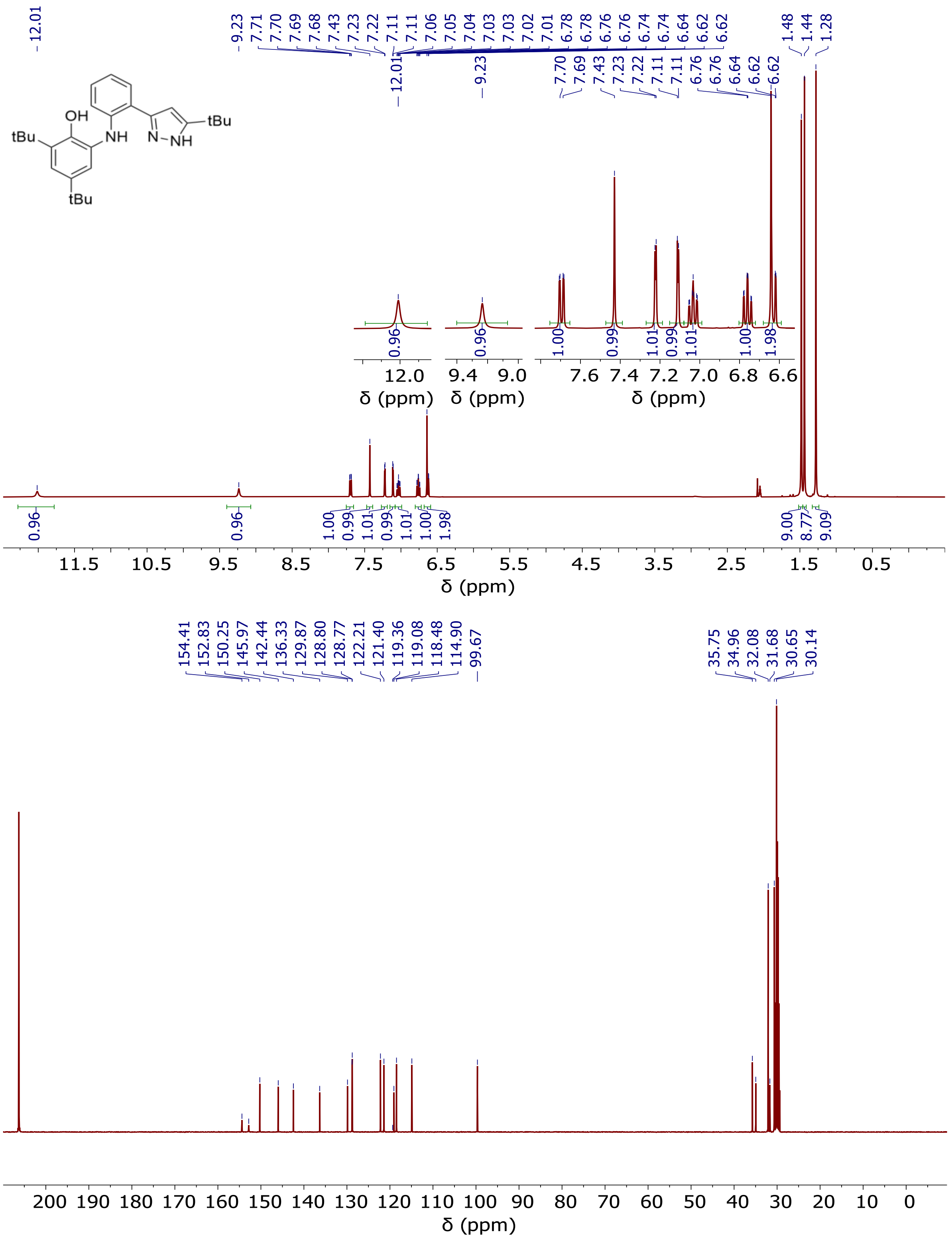

Figure S5. ^{1}H (top) with enlarged aryl region and ^{13}C {^{1}H} (bottom) NMR of **2,4-di-tert-butyl-6-((2-(5-(tert-butyl)-1H-pyrazol-3-yl)phenyl)amino)phenol** in acetone-d_6.

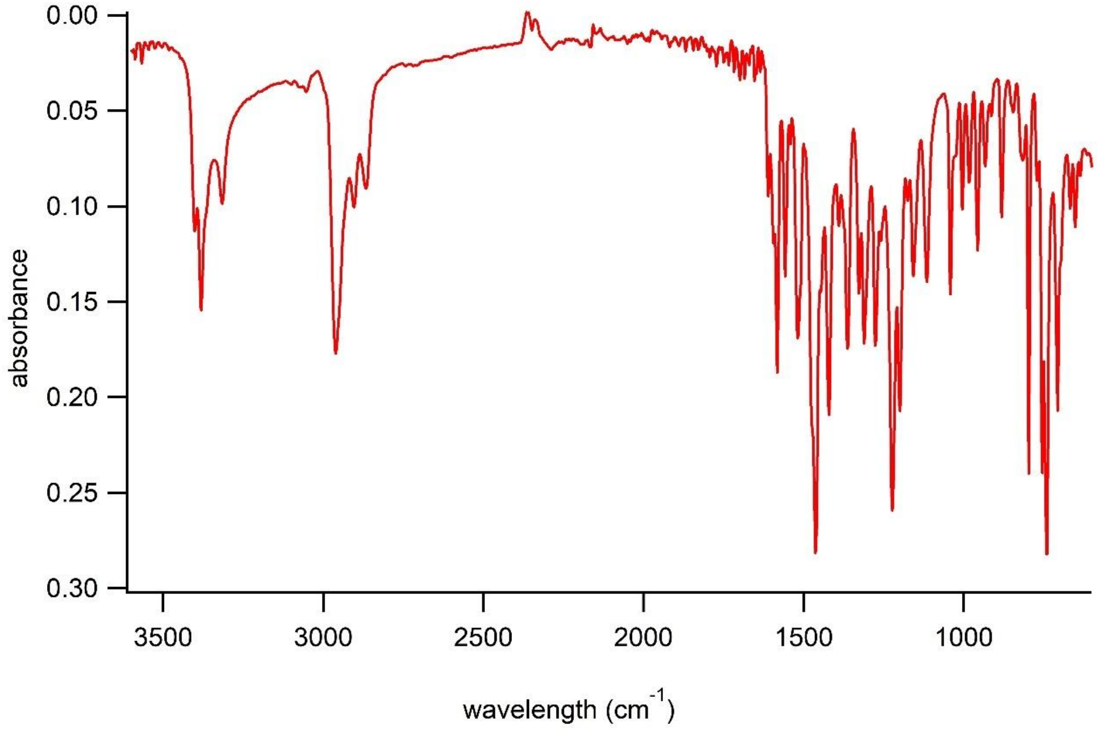

Figure S6. FTIR of **2,4-di-tert-butyl-6-((2-(5-(tert-butyl)-1H-pyrazol-3-yl)phenyl)amino)phenol** (neat).

Synthesis of **1**

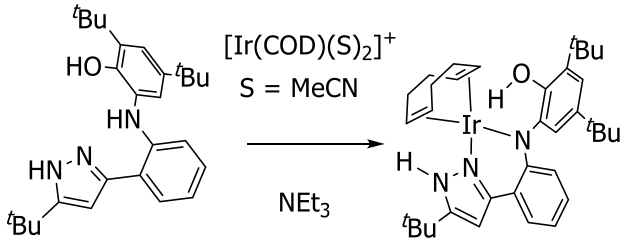

Scheme S4. Synthesis of **1**

2,4-di-tert-butyl-6-((2-(5-(tert-butyl)-1H-pyrazol-3-yl)phenyl)amino)phenol (9.00 mg, 0.02 mmol) and [Ir(COD)(MeCN)$_2$][BF4] (10.0 mg, 0.02 mmol) were weighed in two different vials inside an N_2 atmosphere glovebox. Approximately 0.5 mL of acetone was added to each vial, and the two resulting solutions were mixed by slowly adding the ligand solution to the iridium solution. The solution quickly converted from a deep yellow to a pale tan color. To the vial was added (~5.0 mg, 0.05 mmol triethylamine . The reaction mixture nearly instantly converts from a pale tan to a deep gold. To the solution was added pentane until the solution reached turbidity from the triethylammonium precipitating from the solution.

The reaction mixture was then filtered over celite to yield a clear gold liquid. The solution was concentrated by vacuum until dryness, and the resulting yellow crust was dissolved in minimal pentane, approximately 0.5 mL. The vial containing the pentane solution was placed in a -20° C freezer for 12 hours, yielding small cubic yellow crystals. The crystals were washed with cold pentane, dried on vacuum, then washed with cold acetonitrile and dried in vacuo. The yield for this hproduct is 84%.

^{1}H NMR (500 MHz, CD_2Cl_2) δ 9.52 (d, *J* = 2.4 Hz, 1H), 7.83 (d, *J* = 7.9 Hz, 1H), 7.06 (s, 1H), 7.02 (t, *J* = 7.9 Hz, 1H), 6.83 (s, 1H), 6.77 (s, 1H), 6.69 (t, *J* = 7.4 Hz, 1H), 6.40 (d, *J* = 8.9 Hz, 1H), 6.32 (s, 1H), 3.70 (t, *J* = 7.5 Hz, 1H), 3.55 – 3.42 (m, 1H), 3.34 (t, *J* = 7.6 Hz, 1H), 2.93 – 2.79 (m, 1H), 2.41 – 2.14 (m, 3H), 2.14 – 1.96 (m, 1H), 1.89 – 1.61 (m, 3H), 1.54 (s, 2H), 1.42 (d, *J* = 5.0 Hz, 18H), 1.31 (s, 9H).

^{13}C {^{1}H} NMR (126 MHz, CD_2Cl_2) δ 155.04, 148.41, 147.70, 146.64, 140.88, 137.75, 134.28, 129.55, 128.44, 123.85, 119.08, 116.39, 115.16, 113.43, 98.44, 70.27, 67.07, 59.20, 58.00, 34.81, 34.20, 34.13, 31.96, 31.93, 31.36, 31.33, 30.65, 30.19, 29.52, 29.10, 22.34, 13.83.

IR (solution in DCM) (cm^{-1}): 3609 (s), 3465 (br), 3054 (br), 2972 (s), 2929 (s), 2885 (br), 1467 (br), 1381 (s), 1158 (s), 1126 (s), 1075 (s), 948 (s)

Anal. Calcd. For **1** C, 58.47; H, 6.73; N, 5.84. Found: C, 58.47; H, 6.76; N, 5.80.

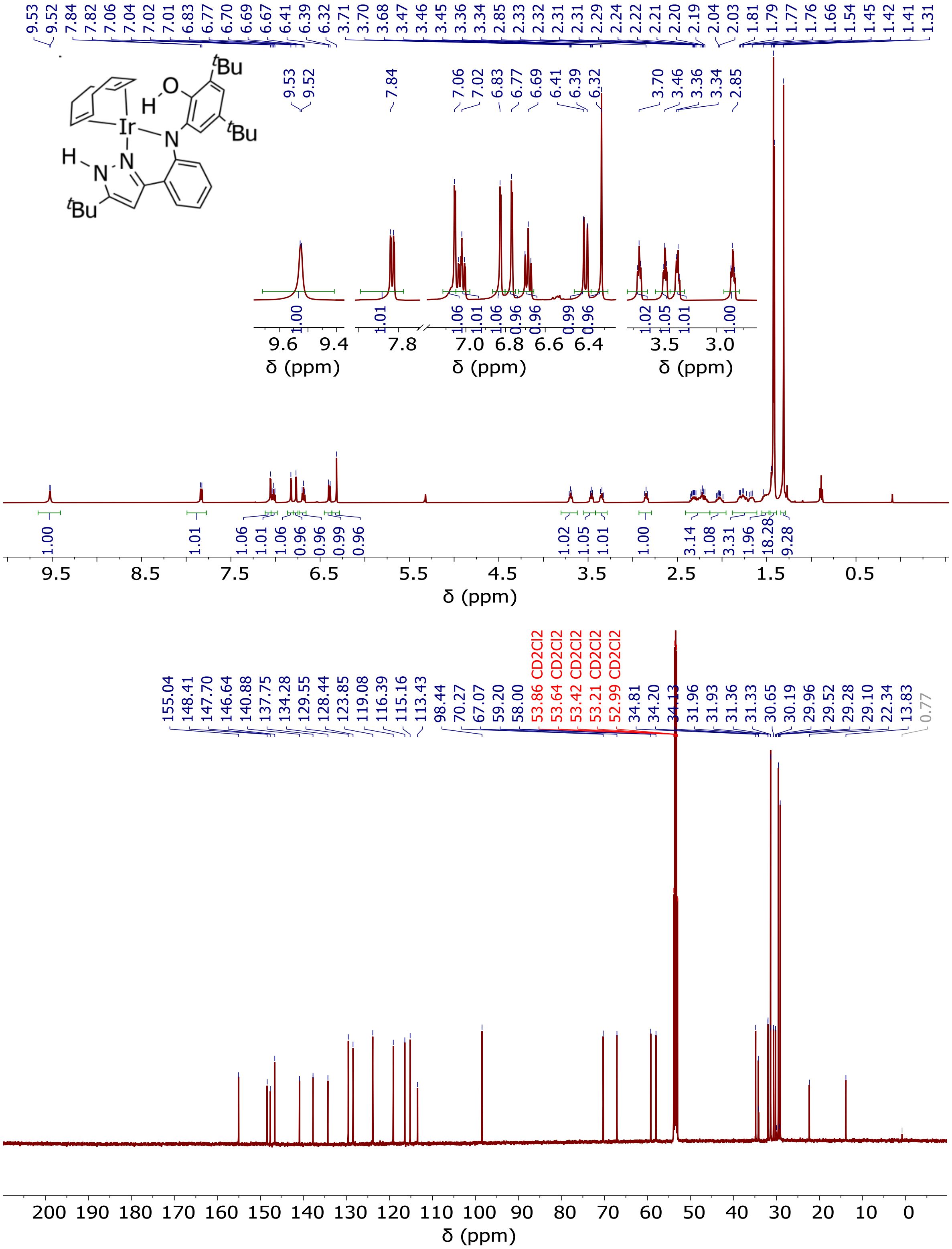

Figure S7. ^{1}H (top) with enlarged aryl region and $^{13}C\{^{1}H\}$ (bottom) NMR of **1** in DCM-d_2.

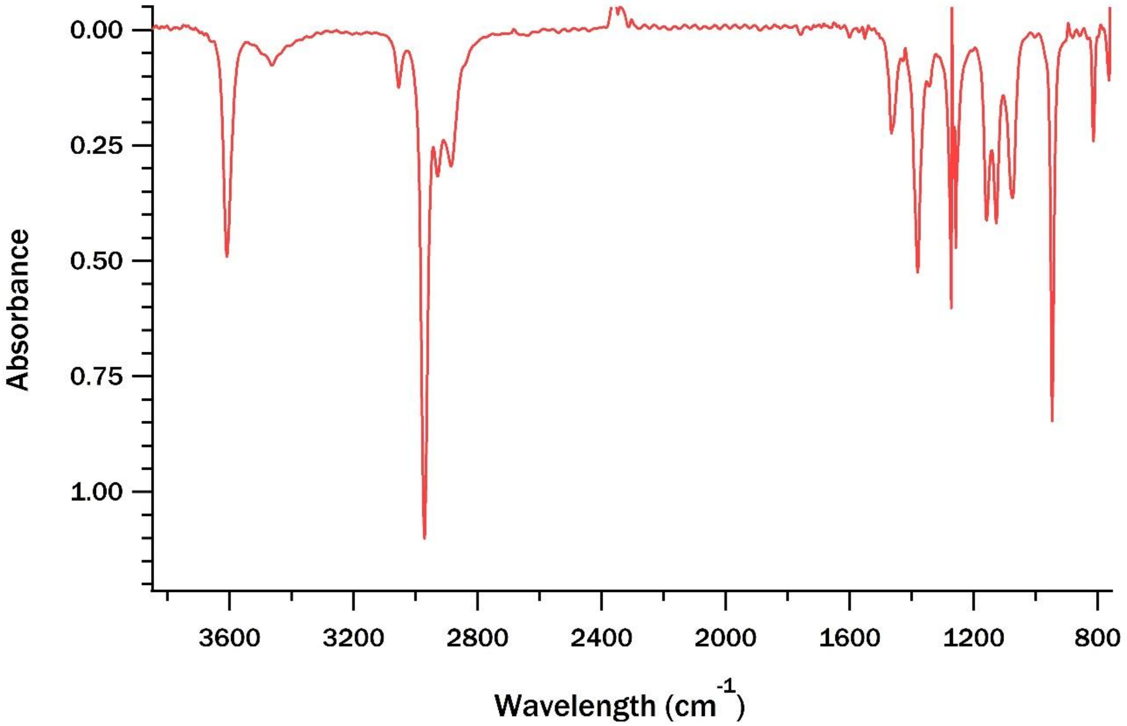

Figure S8. IR (solution in DCM) of **1.**

When dissolved, complex **1** equilibrates with a minor tautomer, which we presume is some form of intramolecular proton migration, as has repeatedly been observed for low-valent Ir(I) bearing protic functional groups.[2-4]

O_2 Gas Addition Procedure

CAUTION! Handling closed systems under pressure can be dangerous, so careful consideration of PPE is required. Pressurized vessels should be contained in secondary containment to contain glass shrapnel in a shattering event. Pressurized J. Young tubes were always wrapped in tin foil and then transported in thick plastic containers. Oxygen is oxidizing and flammable. Oxygen should not be mixed with reducing gases. Oxygen which is pulled off using a Schlenk line which has a condenser set up may condense into liquid oxygen.

The setup for pressurizing J. Young tubes was custom built for our laboratory. The same setup was used in our previous report.[5] The J. Young valves were purchased from Chemglass (CG-513-01, “Valves, NMR Tube, European style”) and affixed to a 7” Medium Walled NMR Tube

from Norell (S-5-500-MW-7, O.D. 4.97 ± 0.013, I.D. 3.43 ± 0.025 mm) by a glassblower. We also fabricated a brass adapter that fits to the NMR valve, with a cap that supports the J. Young valve from the bottom. The threads were organized such that the J. Young valve eventually will be opened by using the cap.

The brass adapter was connected to an O_2 gas cylinder/regulator using PTFE tubing and brass Swagelok. When pressurizing an NMR tube, the apparatus is allowed to outgas (purging) into a fume hood for 10-15 minutes to displace all the air in the tubing/brass adapter. The J. Young tube containing the Ir and solvent (DCM or MeCN) solution is degassed by three successive freeze-pump-thaw cycles on a separate vacuum line to remove any dissolved nitrogen and brought to the pressurization apparatus under static vacuum. Inserting the J. Young tube into the brass apparatus seals the system and allows it to reach the pressure of the regulator. There is some air above the seal of the J. Young valved NMR tube. That air is diluted by removing the inserted tube and allowing the pressurized gas to vent into the fume hood. The NMR tube is then reinserted into the brass adapter. This process of dilution/venting is repeated 10 times. Then the tube is inserted one final time into the brass adapter, and the J. Young valve is opened to allow gas to enter the degassed NMR tube.

Following oxygenation, the J .Young tubes are shaken aggressively for one minute to ensure the oxygen is distributed into the tube. Occasionally, additional reagents must be added into a pressurized J. Young tube. The tube should be degassed following the above procedure before being brought into an anerobic glovebox.

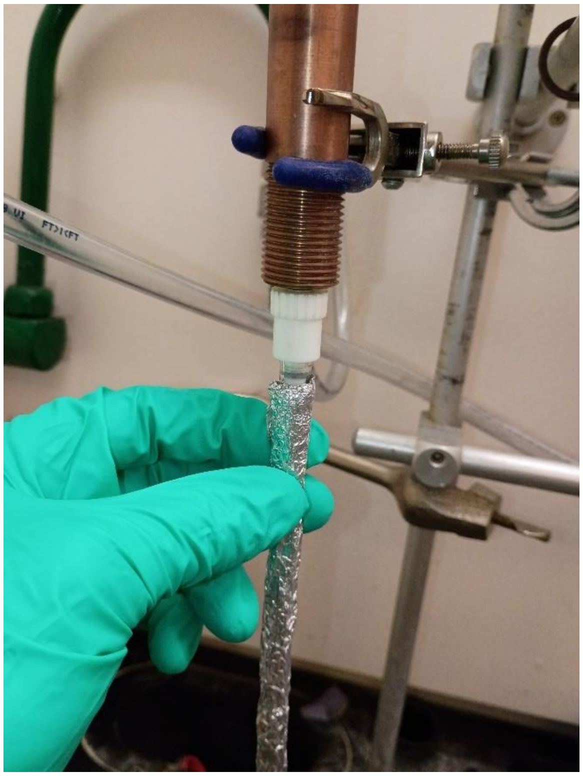

Figure S9. Image showing the custom-made brass adaptor attached to a J. Young valved NMR tube.

Synthesis of 2

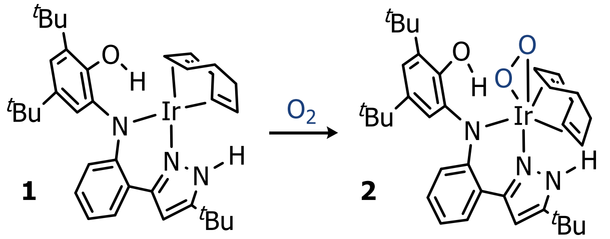

Scheme S5. Synthesis of **2**

1 (5.0 mg, 6.4 μmol) was weighed out in a vial and dissolved in DCM inside an N_2 atmosphere glovebox. The solution was transferred into a J. Young valved NMR tube (J. Young tube). The J. Young tube was then brought out of the glovebox and attached to a Schlenk line. The DCM was removed on the Schlenk line, yielding a yellow crust on the walls of the J. Young tube. This procedure yields a solid with additional surface area and a minimal amount of DCM in it, to help with solubilization. The J. Young tube was brought back into the glovebox, and to it was added MeCN, yielding a yellow-orange solution. The J. Young tube was brought out of

the glovebox and degassed by three cycles of freeze-pump-thaw. The J. Young tube was wrapped in aluminum foil to prevent contact with ambient light. The J. Young tube was connected to a O_2 tank via the apparatus outlined in "O_2 gas addition procedure," and brought to 10 psig of O_2. The J. Young tube was then shaken rapidly for 2 minutes to facilitate the oxygenation reaction. After the oxygen is added, all operations are done with the J. Young tube wrapped in aluminum foil to prevent photoreaction. The observed ^{1}H-NMR changes, and isosbestic UV-Vis spectrum indicate a clean conversion.

^{1}H NMR (500 MHz, CD_2Cl_2) δ 7.80 (d, *J* = 7.8 Hz, 1H), 7.35 (t, *J* = 7.6 Hz, 1H), 7.19 (t, *J* = 7.6 Hz, 1H), 7.11 (d, *J* = 7.8 Hz, 1H), 6.88 – 6.76 (m, 1H), 6.56 (s, 1H), 6.32 (d, *J* = 10.8 Hz, 2H), 5.95 (t, *J* = 7.9 Hz, 1H), 4.98 (q, *J* = 8.4 Hz, 1H), 4.28 (t, *J* = 7.3 Hz, 1H), 2.95 (s, 1H), 2.51 (d, *J* = 40.3 Hz, 5H), 1.97 (s, 1H), 1.80 (s, 1H), 1.50 (s, 9H), 1.35 (s, 10H), 1.15 (s, 9H).

*Note: The spectra of **2** always contains a small quantity of minor isomer. The ratio of **2** to this minor isomer is constant across a series of measurements, using multiple methods to synthesize **2**. Accordingly, we assign this as a thermodynamically competitive isomer that establishes an equilibrium with **2**. See below sections for details.

^{13}C {^{1}H} NMR (126 MHz, CD_2Cl_2) δ 176.78, 165.80, 162.41, 152.50, 140.14, 135.11, 130.57, 128.73, 128.25, 125.69, 124.00, 122.04, 115.45, 108.37, 104.57, 100.39, 93.52, 88.76, 37.24, 36.30, 34.16, 32.16, 31.83, 31.39, 30.83, 30.65, 30.44, 29.45, 28.42, 28.25, 28.07, 22.37, 13.87.

IR (Solution phase, in DCM (cm^{-1})) 3686 (br), 3601 (br), 2963 (s), 2922 (br), 2908 (br), 2871 (s), 1711 (s), 1632 (s), 1604 (s), 1544 (s), 1488 (s), 1466 (s), 1363 (s), 1317 (s), 1243 (s), 1173 (s), 1124 (s), 997 (s), 916 (s)

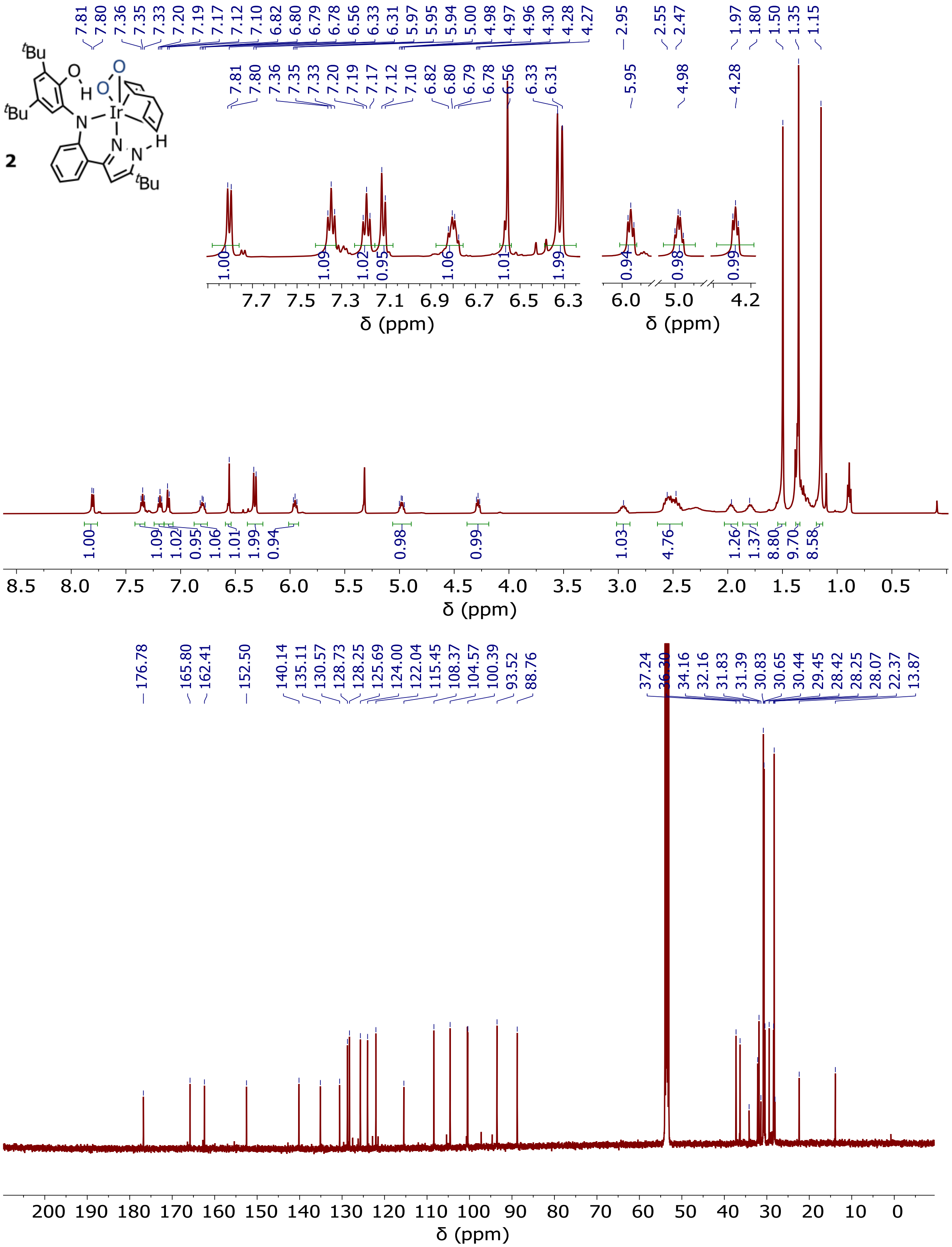

2

Figure S10. ^{1}H (top) with enlarged aryl region and ^{13}C {^{1}H} (bottom) NMR of **2** in DCM-d2.

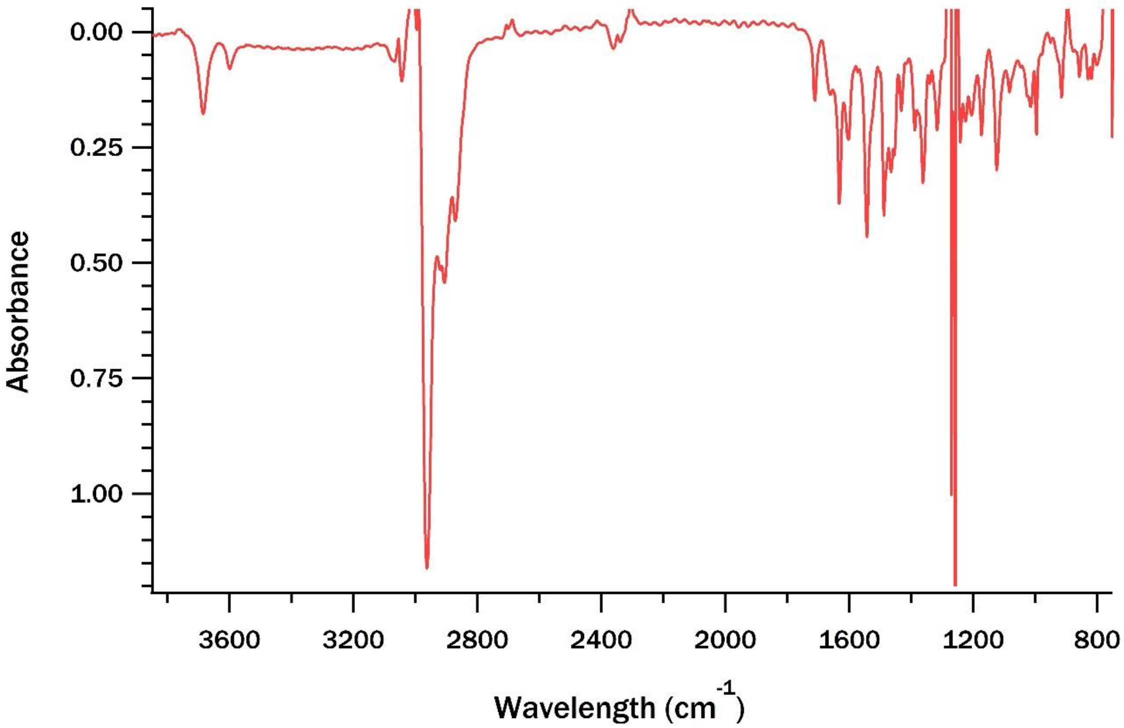

Figure S11. IR (solution in DCM) of **2.**

When dissolved, complex **2** equilibrates with a minor tautomer, which we presume is some form of intramolecular proton migration, as has repeatedly been observed for low-valent Ir(I) bearing protic functional groups.[2-4]

Synthesis of 3

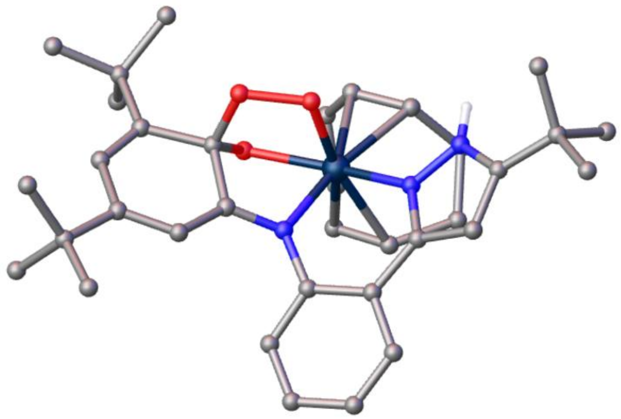

Figure S12. X-ray structure of 3

1 (5.0 mg, 0.0064 mmol) was weighed out in a vial and dissolved in DCM inside an N_2 atmosphere glovebox. The solution was transferred into a J. Young valved tube and degassed by three cycles of freeze-pump-thaw. The J. Young tube was wrapped in aluminum foil to create a dark environment. The J. Young tube was connected to a O_2 tank via a bespoke Swagelok adapter which permits the direct addition of a pressurized gas to a J. Young tube. Using the Swagelok apparatus, the J. Young tube was pressurized to 10 psig of O_2. The J. Young tube was then shaken rapidly for 2 minutes to facilitate the oxygenation reaction. The DCM was then removed from the J. Young tube by freezing in liquid nitrogen and then opening to vacuum on a Schlenk line. The J. Young tube was removed from the Schlenk line and brough into an inert atmosphere glovebox, where the resulting red crust was then dissolved in MeCN (First dissolving in DCM and oxygenating is necessary because **1** is practically insoluble in MeCN). TEMPO (10.0 mg, 0.06 mmol) was added to the J. Young valved tube causing the quick precipitation of small red crystals of **3**. The liquid was removed from the J. Young tube and the crystals were washed three times with 0° C MeCN and dried in vacuo. The yield for this product is 3.4 mg (68%).

The complex **3** cannot be studied in pure MeCN solution, see **Relationship between [2 and 3].**

FTIR (ATR) cm^{-1}: 2964 (s), 2917 (s), 2874 (br), 1631 (s), 1536 (s), 1468 (s), 1242 (s), 1118 (s), 904 (s), 775 (s).

Anal. Calcd. For **3** $C_{35}H_{47}IrN_3O_3$: C, 56.05; H, 6.32; N, 5.60. Found: C, 56.073; H, 6.128; N, 5.571.

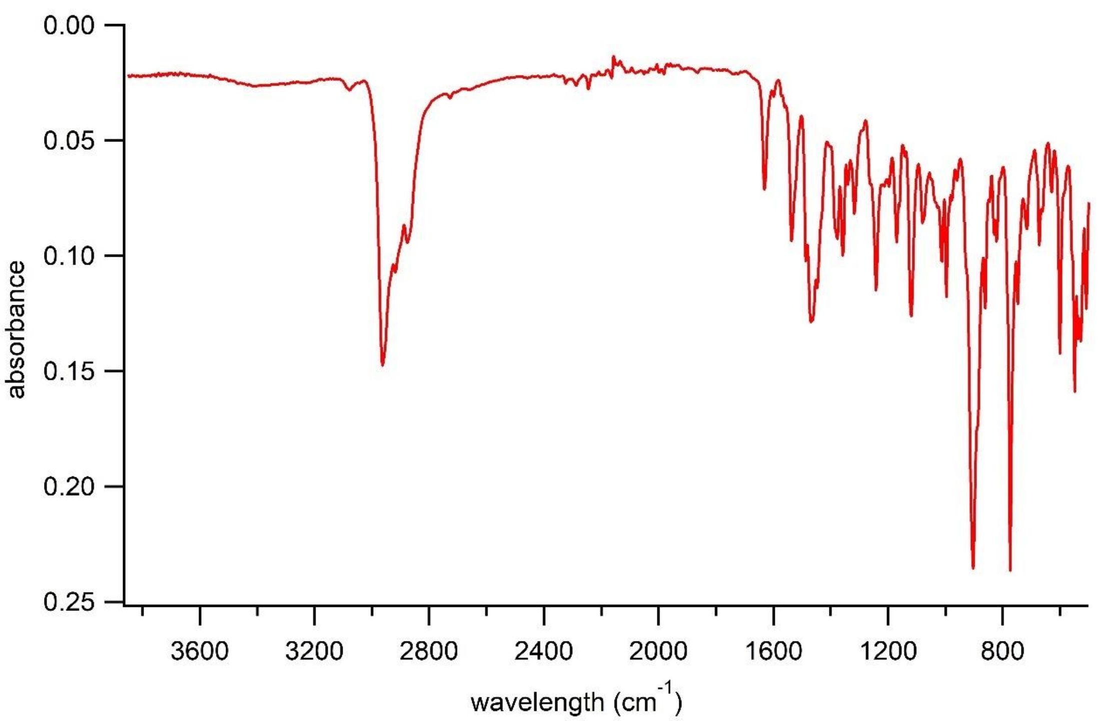

Figure S13. ATR (neat) of **3**.

Relationship between [**2** and **3**].

The relationship between **2** and **3** is that the latter complex is deficient by one H atom. Synthetically, we trigger this reaction by adding one equivalent of TEMPO•.

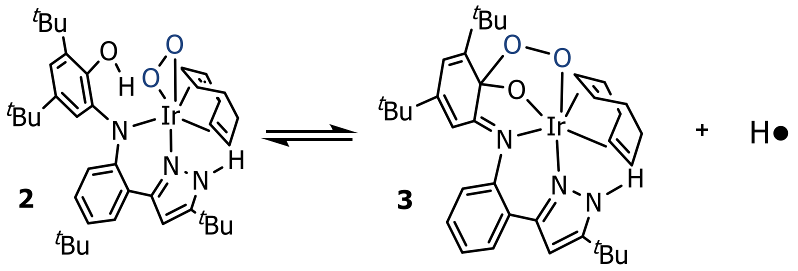

Scheme S6. H atom relationship between **2** and **3**

Although **3** can be isolated as a solid, resuspending those crystals in $C(H/D)_3CN$ seems to regenerate the precursor peroxo **2** in 70% yield. So, dissolution of **3** corresponds to a second synthetic procedure for generating **2**, which we presume occurs by the abstraction of an H•/D• from the $C(H/D)_3CN$.

With two preparations of **2**, we elected to compare UV-Vis spectra of **2** from: (a) reaction of **1** + O_2 and (b) redissolution of **3** in CH_3CN. Similar absorption spectra were observed in both cases (Figure S14).

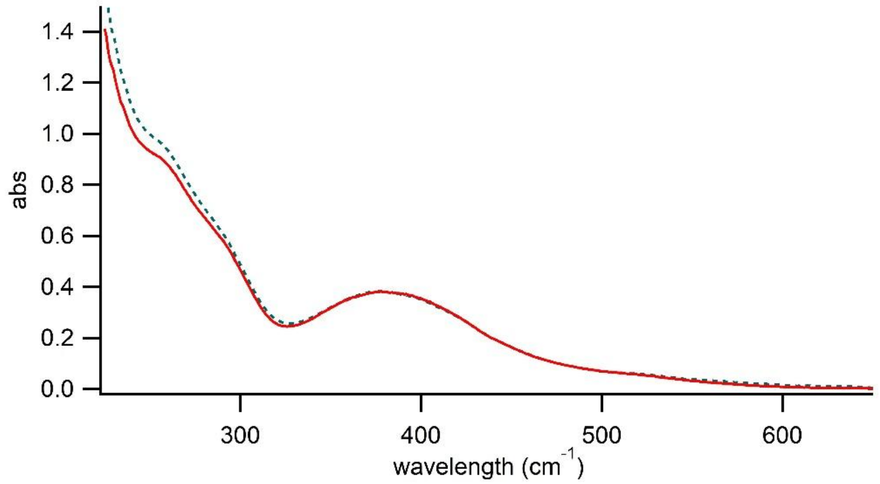

Figure S14. UV-Vis absorption spectrum of **2** from the oxygenation of **1** (blue dashed) and from the dissolving of **3** in MeCN (red).

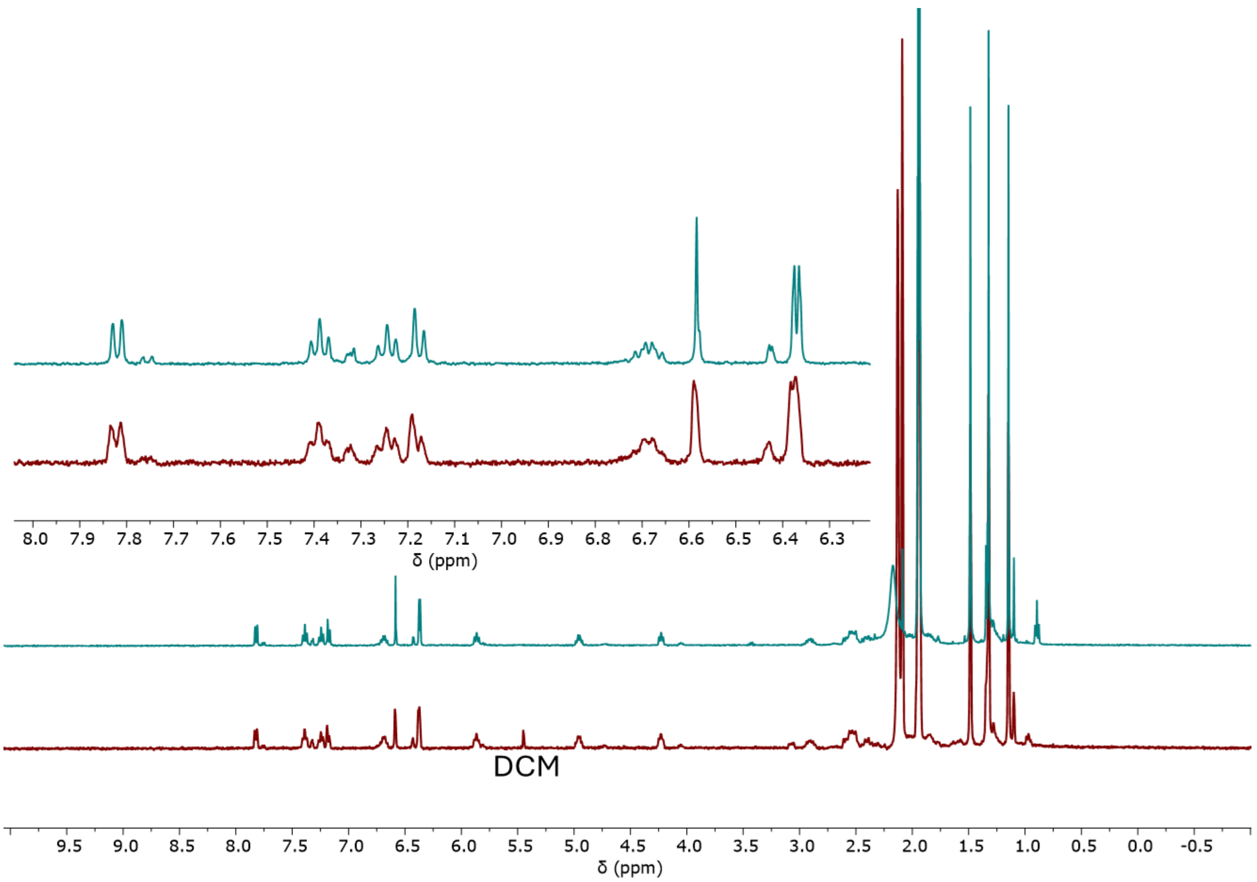

Figure S15. The ^{1}H-NMR of **2** when generated from the oxygenation of **1** (top in blue) and from the dissolving of **3** in CD_3CN (bottom in red). Trace DCM added for solubility.

Synthesis of 4

2

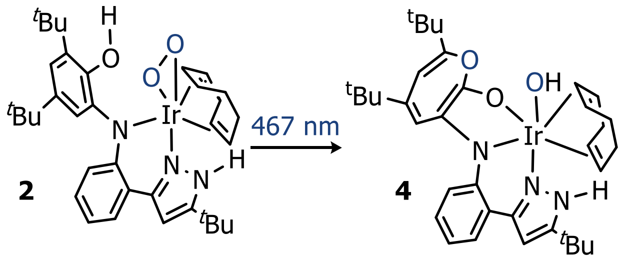

4

Scheme S7. Synthesis of 4

1 (5.0 mg, 6.4 μmol) was weighed out in a vial and dissolved in DCM inside an N_2 atmosphere glovebox. The solution was transferred into a J. Young valved NMR tube (J. Young tube). The J. Young tube was then brought out of the glovebox and attached to a Schlenk line and degassed by three cycles of freeze-pump-thaw. The J. Young tube was wrapped in aluminum foil to prevent contact with ambient light.. The J. Young valved tube

was connected to a O_2 tank via a bespoke Swagelok adapter which permits the direct addition of a pressurized gas to a J. Young tube. Using the Swagelok apparatus, the J. Young tube was pressurized to 10 psig of O_2. The J. Young tube was then shaken rapidly for 2 minutes to facilitate the oxygenation reaction. The J. Young tube was then placed in front of a LuzChem LED Illuminator and irradiated with a 467 nm LED bulb for 10 minutes at 0.03 bulb intensity with the tube positioned 35 mm away from the LED. 1H-NMR is used to track the progression of the reaction and the irradiation is stopped when the reaction is completed.

^{1}H NMR (500 MHz, CD_3CN) δ 7.77 (d, *J* = 7.9 Hz, 1H), 7.41 – 7.33 (m, 1H), 7.27 (t, *J* = 7.0 Hz, 1H), 7.22 (d, *J* = 7.3 Hz, 1H), 7.06 – 6.93 (m, 1H), 6.57 (s, 1H), 6.56 (d, *J* = 1.7 Hz, 1H), 5.91 (s, 1H), 5.81 (d, *J* = 1.7 Hz, 1H), 4.93 (s, 1H), 3.87 (t, *J* = 7.6 Hz, 1H), 2.60 (s, 2H), 2.52 (s, 2H), 2.37 – 2.27 (m, 1H), 1.34 (s, 10H), 1.28 (s, 10H), 1.14 (s, 9H).

^{13}C {^{1}H} NMR (126 MHz, CD_2Cl_2) δ 180.29, 165.27, 164.06, 163.19, 141.78, 133.96, 129.61, 128.59, 128.05, 126.43, 126.19, 122.06, 110.23, 105.09, 104.13, 101.21, 100.55, 89.38, 88.22, 38.26, 37.86, 32.47, 31.11, 31.08, 31.02, 30.98, 30.61, 30.35, 30.32, 30.01, 29.32, 28.91, 28.80, 28.63, 28.31, 27.35, 25.08.

IR (NaCl solution FTIR cell) in DCM (cm^{-1}) 3686 (br), 3597 (br), 2967 (s), 2906 (s), 1685 (s), 1636 (s), 1562 (s), 1488 (s), 1465 (s), 1363 (s), 1099 (s), 1070 (s).

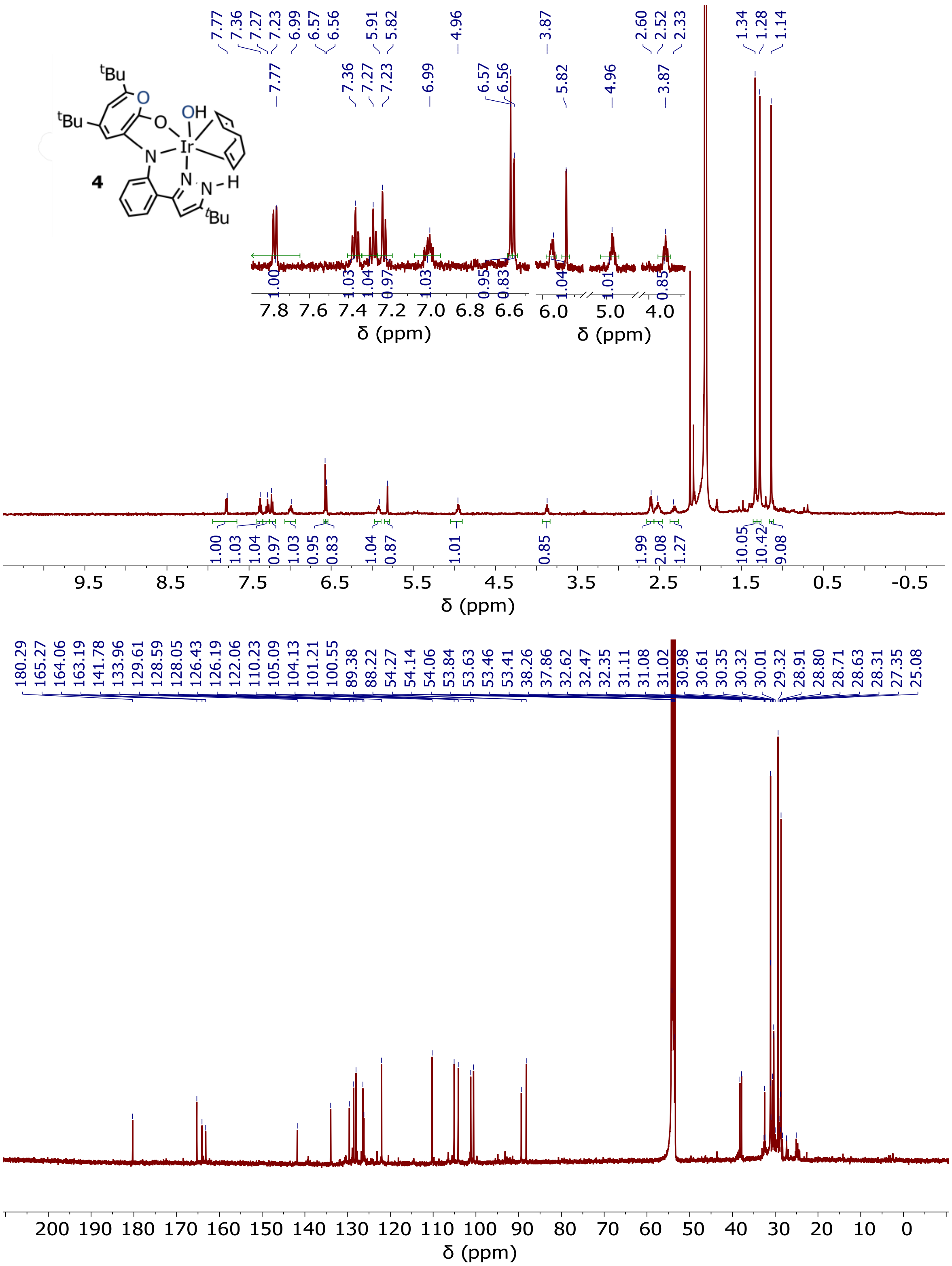

Figure S16. ^{1}H with enlarged aryl region of **4** in MeCN-d3 and $^{13}C\{^{1}H\}$ NMR in DCM-d2.

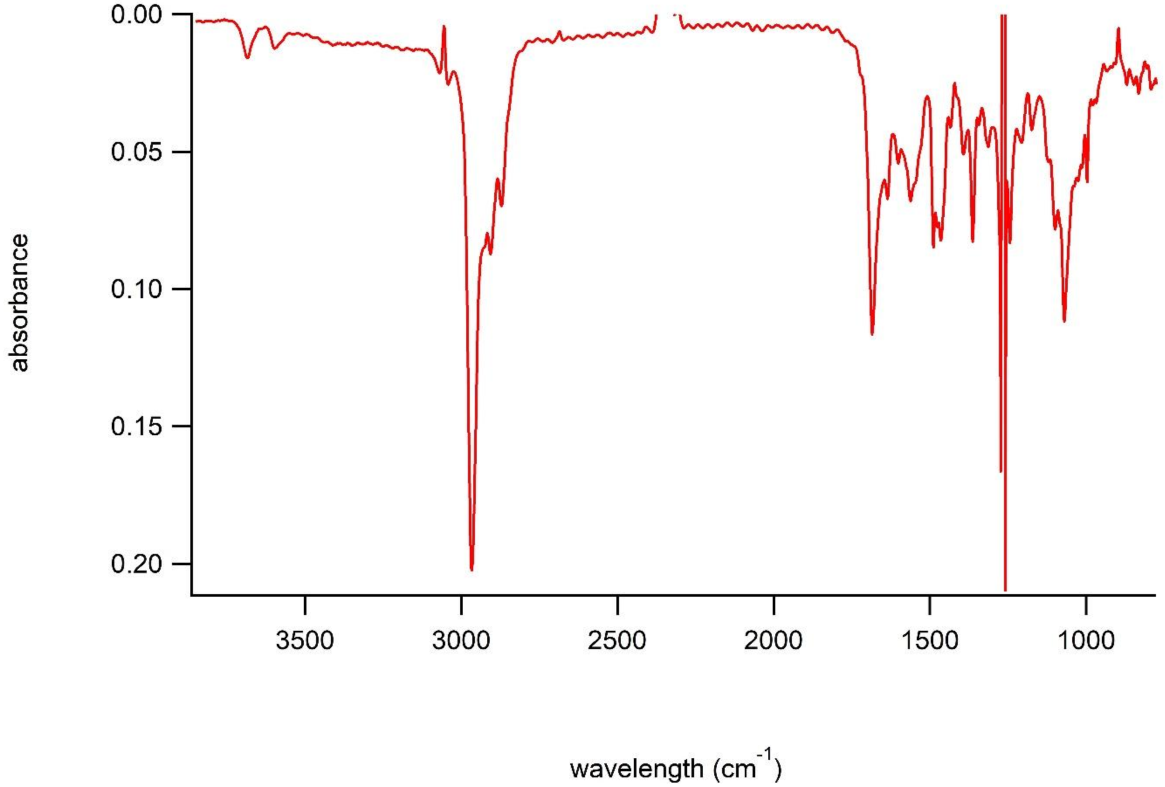

Figure S17. IR (solution in DCM) of **4**.

The photoreaction of 2 can be monitored by UV-Vis. 0.39 mM DCM solutions of **2** were prepared in a quartz cuvette. A UV-Vis spectrum was taken, and then the cuvette was placed 35 mm away from a LuzChem photolyzer with a blue LED. The cuvette was photolyzed for 5 seconds at 0.1 intensity, shaken, and a UV-Vis spectrum was collected. The cuvette was photolyzed for 10 additional seconds, shaken, and a UV-Vis spectrum was taken. This process was repeated until the reaction reached completion.

When 2 is irradiated by a blue LED with emissions centered at 467 nm, two distinct kinetic regions are observed. First, there is an initial rapid change to the absorption spectrum after 5 seconds. The reaction is too fast to monitor by ^{1}H-NMR. Continued photolysis results in a slower second regime. Isosbestic points were observed at 328 and 447 nm. The overall profile is consistent with an A → B → C reaction mechanism, where A → B is fast and B → C is slow. The isosbestic points emerge when the absorbance from **2** (A in this example) corresponds to a negligible amount of the total absorbance from A, B, and C.

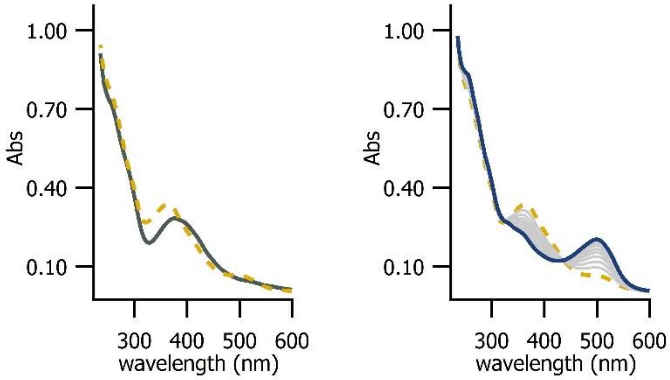

Figure S18. Left: UV-Vis absorbance plot of the initial 5 seconds of photolysis. **2** (solid blue line) converts to an intermediate (dashed yellow line). Right: Continued photolysis of the intermediate (dashed yellow line) for 300 seconds results in the complete conversion to **4** (solid blue line).

5

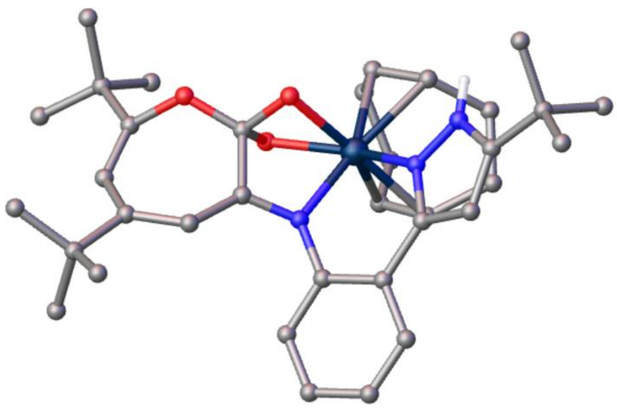

Figure S19. X-Ray structure of 5

1 (5.0 mg, 0.0064 mmol) was weighed out in a vial and dissolved in DCM inside an N_2 atmosphere glovebox. The solution was transferred into a J. Young valved NMR tube (J. Young tube). The J. Young tube was then brought out of the glovebox and attached to a Schlenk line and degassed by three cycles of freeze-pump-thaw. The J. Young tube was wrapped in aluminum foil to create a dark environment. The J. Young tube was connected to a O_2 tank via a bespoke Swagelok adapter which permits the direct addition of a pressurized gas to a J. Young tube. Using the Swagelok apparatus, the J. Young tube was pressurized to 10 psig of

O_2. The J. Young tube was then shaken rapidly for 2 minutes to facilitate the oxygenation reaction. The J. Young tube was then place in front of a LuzChem LED Illuminator and irradiated with a 467 nm LED bulb for 10 minutes at 0.03 bulb intensity with the tube positioned 35 mm away from the LED. Then, the J. Young tube is attached to a Schlenk line, and the DCM was then removed from the J. Young tube by freezing in liquid nitrogen and then opening to vacuum on a Schlenk line. The J. Young tube was brought into an inert atmosphere glovebox, where the resulting yellow crust was then dissolved in MeCN. (First dissolving in DCM and oxygenating is necessary because **1** is practically insoluble in MeCN.) TEMPO (10.00 mg, 0.0639 mmol) was added to the J. Young tube, and after 24 hours was yielded small yellow crystals of **5**. The liquid was removed from the J. Young tube and the crystals were washed three times with 0° C MeCN and dried in vacuo. The yield for this product is 2.3mg 45%.The yield for this product is 2.3mg 45%.

Anal. Calcd. For **5** $C_{35}H_{47}IrN_3O_3$: C, 56.05; H, 6.32; N, 5.60. Found: C, 56.252; H, 6.300; N, 5.558.

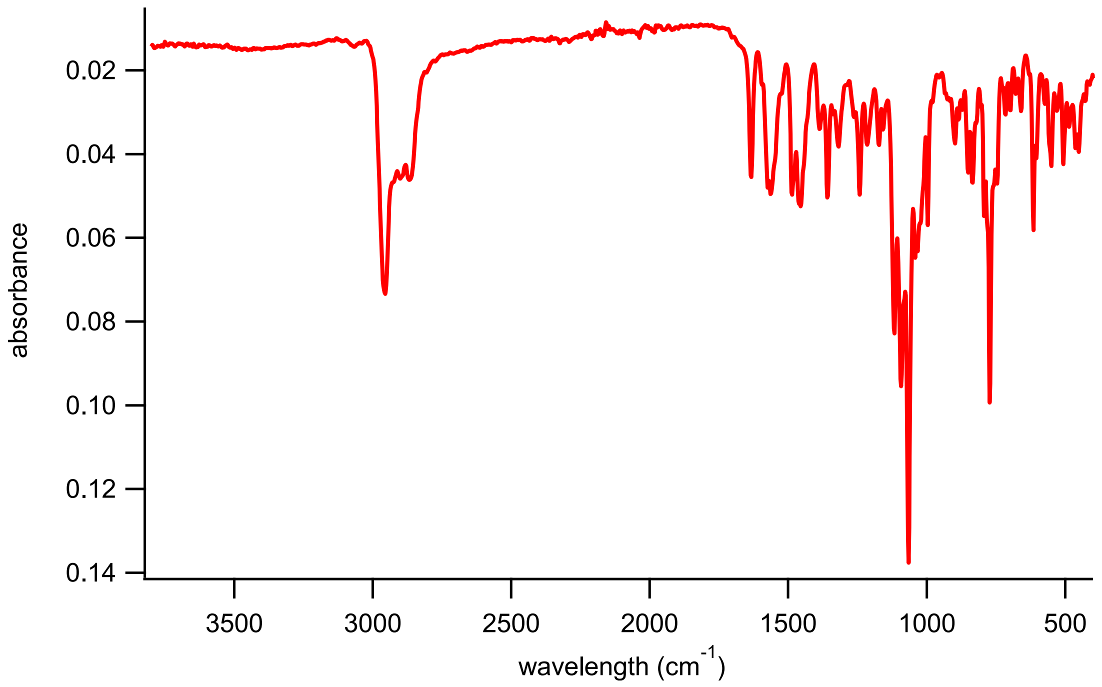

Figure S20. ATR of **5** (neat)

Relationship between [**4** and **5**].

The relationship between **4** and **5** is that the latter complex is deficient by one H atom. Synthetically, we trigger this reaction by adding one equivalent of TEMPO•.

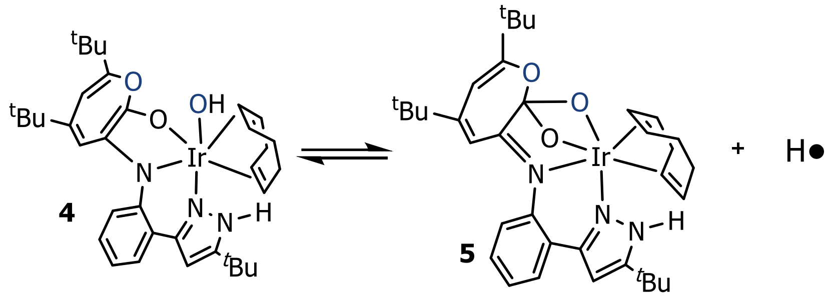

Scheme S8. H atom relationship between **4** and **5**

Although **5** can be isolated as a solid, resuspending those crystals in C(H/D)$_3$CN seems to regenerate the precursor peroxo **4** in 63% yield. So, dissolution of **5** corresponds to a second synthetic procedure for generating **4**, which we presume occurs by the abstraction of an H•/D• from the C(H/D)$_3$CN.

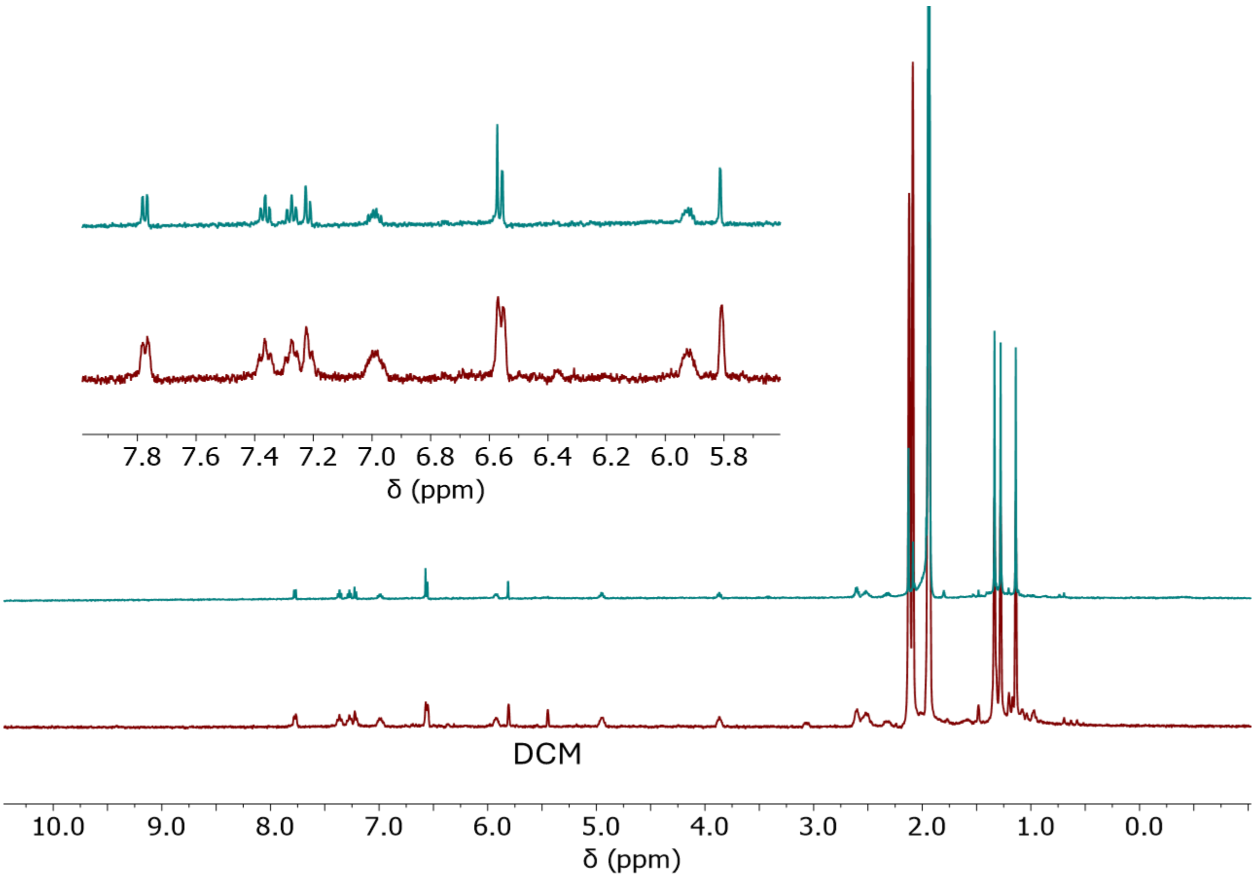

Figure S21. The ^1H NMR of **4** when generated from the photolysis of **2** (top in blue) and from the dissolving of **5** in CD_3CN (bottom in red). Trace DCM added for solubility.

O_2 Gas Addition Procedure

CAUTION! Handling closed systems under pressure can be dangerous, so careful consideration of PPE is required. Pressurized vessels should be contained in secondary containment to contain glass shrapnel in a shattering event. Pressurized J. Young tubes were always wrapped in tin foil and then transported in thick plastic containers. Oxygen is oxidizing and flammable. Oxygen should not be mixed with reducing gases. Oxygen which is pulled off using a Schlenk line which has a condenser set up may condense into liquid oxygen.

The setup for pressurizing J. Young tubes was custom built for our laboratory. The same setup was used in our previous report.[5] The J. Young valves were purchased from Chemglass (CG-513-01, "Valves, NMR Tube, European style") and affixed to a 7" Medium Walled NMR Tube from Norell (S-5-500-MW-7, O.D. 4.97 ± 0.013, I.D. 3.43 ± 0.025 mm) by a glassblower. We also fabricated a brass adapter that fits to the NMR valve, with a cap that supports the J. Young valve from the bottom. The threads were organized such that the J. Young valve eventually will be opened by using the cap.

The brass adapter was connected to an O_2 gas cylinder/regulator using PTFE tubing and brass Swagelok. When pressurizing an NMR tube, the apparatus is allowed to outgas (purging) into a fume hood for 10-15 minutes to displace all the air in the tubing/brass adapter. The J. Young tube containing the Ir and solvent (DCM or MeCN) solution is degassed by three successive freeze-pump-thaw cycles on a separate vacuum line to remove any dissolved nitrogen and brought to the pressurization apparatus under static vacuum. Inserting the J. Young tube into the brass apparatus seals the system and allows it to reach the pressure of the regulator. There is some air above the seal of the J. Young valved NMR tube. That air is diluted by removing the inserted tube and allowing the pressurized gas to vent into the fume hood. The NMR tube is then reinserted into the brass adapter. This process of dilution/venting is repeated 10 times. Then the tube is inserted one final time into the brass adapter, and the J. Young valve is opened to allow gas to enter the degassed NMR tube.

Following oxygenation, the J .Young tubes are shaken aggressively for one minute to ensure the oxygen is distributed into the tube. Occasionally, additional reagents must be added

into a pressurized J. Young tube. The tube should be degassed following the above procedure before being brought into an anerobic glovebox.

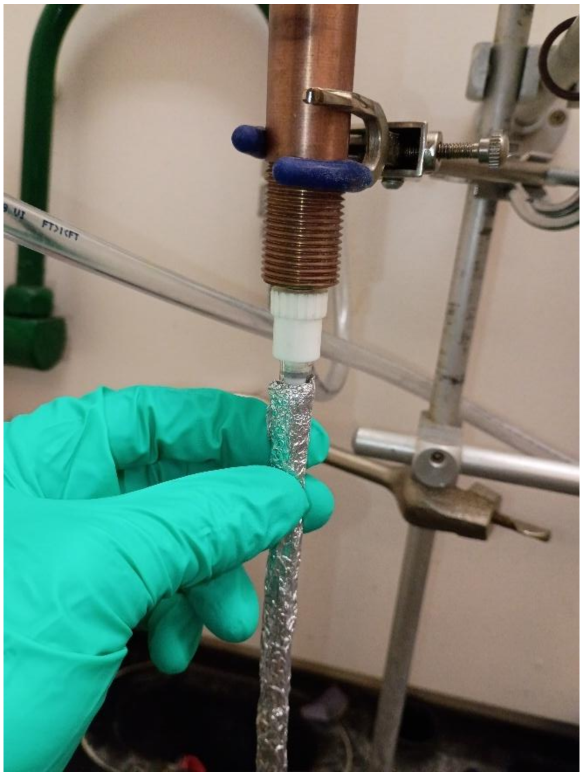

Figure S22. Image showing the custom-made brass adaptor attached to a J. Young valved NMR tube.

Kinetics

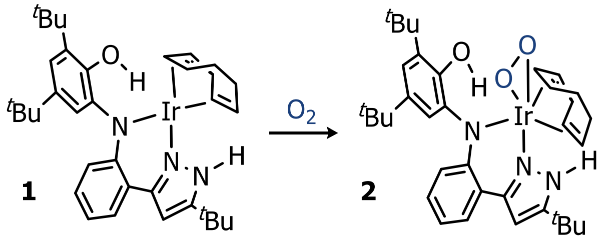

Scheme S9. Oxygenation of **1** to **2**

All kinetics experiments were performed in air-tight quartz UV-Vis cuvettes with screw caps featuring a membrane-style compressible septa (from Spectrocell). The DCM used to make

the kinetic solutions was refluxed over P_2O_5 overnight before being distilled. Each cuvette was filled with 0.2 mL of **1** stock solution. The total targeted volume for the run was 2.2 mL. So, of the remaining 2.0 mL of solution, a quantity **x** was added to the solution of **1** as additional dry DCM. (2.0 – **x**) mL was added as an O_2-saturated DCM solution. The quantity **x** can be varied to generate solutions at different O_2 concentrations.

In a representative procedure, solutions of **1** were made by dissolving **1** into dry DCM in an inert atmosphere glovebox. Solutions of O_2 were made by vigorously sparging O_2 into a Schlenk flask in a room temperature waterbath for 30 minutes to create a saturated solution of O_2(the solubility of O_2 in DCM changes with temperature, so it is important to maintain a consistent temperature). The concentration of a saturated solution of O_2 in DCM has been reported to be 3.91 mM.[3]

Kinetic runs were performed at 5 different concentration of O_2 spanning an order of magnitude. For the most dilute runs, 0.2 mL of 0.39 mM **1** solution in DCM was added to 1.8 mL of dry DCM in a quartz cuvette in a glovebox. The cuvette was capped with a pierceable cap and brought out of the glovebox. 0.2 mL of 3.9 mM O_2 solution was then removed from the flask it was sparging in using a Leur lock syringe. The syringe containing the oxygen solution was inserted into the cuvette at the same time as a narrow vent needle (to ensure consistent pressure). The oxygen solution was then injected, the needles remove, and the cuvette was shaken before the cuvette was placed into the UV-Vis to be monitored.

The same procedure was completed for the other four concentrations of O_2 using the following ratios of stock solutions.

Table S1. Volumes of stock solutions used for kinetic runs.

	0.39 mM 1 soln. (mL)	3.91 mM O_2 soln. (mL)	DCM (mL)
Run 1	0.20	2.00	0.00
Run 2	0.20	1.55	0.45
Run 3	0.20	1.10	0.90
Run 4	0.20	0.65	1.35
Run 5	0.20	0.20	1.80

Physical properties of compounds can be used to monitor the kinetics of the reaction so long as the physical observable is proportional to the concentration of the compound. For the reaction of **1** -> **2**, the absorbance at a fixed wavelength can be monitored as the reaction progresses, and the change in absorbance can be used to determine the k_{obs} of the reaction. In theory, any wavelength can be used so long as there is a difference in absorbance between **1** and **2** at that wavelength. A good practice is to monitor the reaction at multiple wavelengths to ensure the value for k_{obs} is the same at each wavelength.

The oxygenation of **1** is assumed to proceed with the following second order rate constant.

-d[**1**]/dt = k_2[**1**][O_2]

By flooding the system with gross excess (>10 equivalents) O_2, the pseudo-first order rate constant can be determined.

-d[**1**]/dt = k_{obs}[**1**]

To determine k_{obs}, the data was fit to a pseudo first-order exponential equation derived in Chemical Kinetics and Reaction Mechanisms (page 22-23).[6]

$abs_t = abs_\infty + (abs_0 - abs_\infty)^{-kt}$

where abs_t is the absorbance at any given time, abs_∞ is the absorbance value upon reaction completion, abs_0 is the initial absorbance value, k is the pseudo-first order rate constant k_{obs} and t is the time.

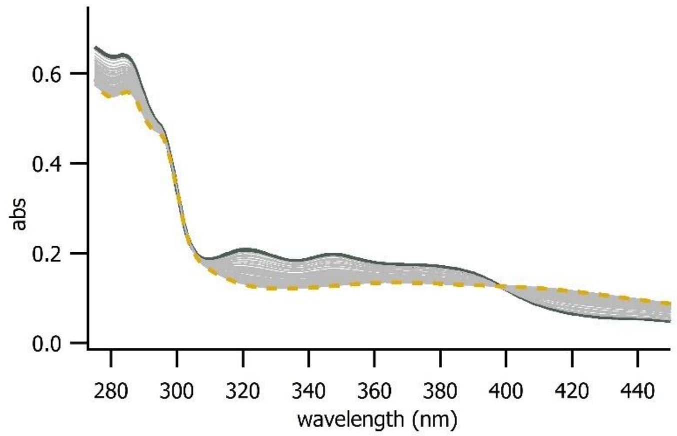

Figure S23. UV-Vis absorption spectrum of the conversion of **1** (solid green) to **2** (dotted yellow).

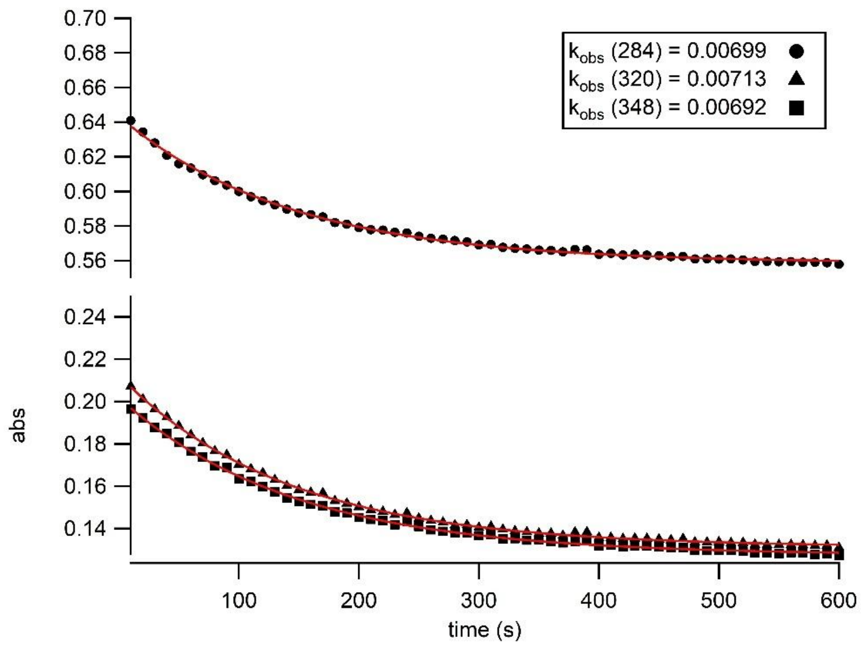

Figure S24. Single kinetic trace of the reaction of **1** to **2** monitored at 3 different wavelengths in DCM (circle at 284 nm, triangle at 320 nm, square at 348 nm).

The fitting procedure was performed for the most dilute case at three different wavelengths, and 3 virtually identical rate constants were obtained. The strong correlation of the fit is consistent with first-order kinetics with respect to **1**.

A series of k_{obs} values were measured at different concentrations of O_2 (preparation discussed above) spanning an order of magnitude.

Table S2. Concentration 1

time (s)	run 1 abs	run 2 abs	run 3 abs
4	0.185168	0.121527	0.140705
8	0.175322	0.120811	0.137639
12	0.172733	0.118383	0.134844
16	0.170207	0.118044	0.131821
20	0.168461	0.118809	0.129256
24	0.165846	0.117868	0.126828
28	0.163686	0.117043	0.12509
32	0.161727	0.116827	0.122253
36	0.159628	0.116683	0.120346
40	0.157768	0.116385	0.118448
44	0.155975	0.116497	0.116657
48	0.154362	0.116499	0.114963
52	0.152805	0.11633	0.113198
56	0.151319	0.116574	0.111728

60	0.149901	0.115748	0.110268
64	0.148478	0.115836	0.108799
68	0.147168	0.115618	0.108756
72	0.145979	0.115195	0.106123
76	0.144773	0.116655	0.105259
80	0.143468	0.116012	0.103909
84	0.141946	0.115145	0.102926
88	0.140639	0.115161	0.101858
92	0.139207	0.114565	0.100859
96	0.138097	0.115377	0.099863
100	0.136946	0.115369	0.09901
104	0.135968	0.114983	0.098234
108	0.135158	0.115015	0.097431
112	0.134179	0.114572	0.096755
116	0.133409	0.114957	0.096011
120	0.132808	0.11484	0.095308
124	0.131952	0.114867	0.094849
128	0.131268	0.114631	0.094039
132	0.130409	0.114343	0.093655
136	0.129717	0.113983	0.092996
140	0.129235	0.115023	0.092434
144	0.128569	0.114789	0.091911
148	0.128472	0.114346	0.091467
152	0.128642	0.114873	0.090935
156	0.12819	0.114034	0.090506
160	0.127955	0.114683	0.090058
164	0.127175	0.114698	0.089817
168	0.125855	0.114338	0.089269
172	0.12539	0.114163	0.088933
176	0.124645	0.114373	0.088614
180	0.12411	0.114053	0.088292
184	0.124192	0.114782	0.087865
188	0.12358	0.11424	0.087659
192	0.123377	0.114719	0.08728
196	0.122727	0.114138	0.086939
200	0.122073	0.113312	0.086601
204	0.122035	0.113147	0.086434
208	0.121647	0.112901	0.086142
212	0.121013	0.11282	0.085947
216	0.121142	0.112423	0.086002
220	0.12058	0.112195	0.085545
224	0.11978	0.112431	0.085316
228	0.12003	0.112411	0.085096
232	0.119733	0.112431	0.084839
236	0.119351	0.112506	0.084679

240	0.119193	0.112528	0.084548

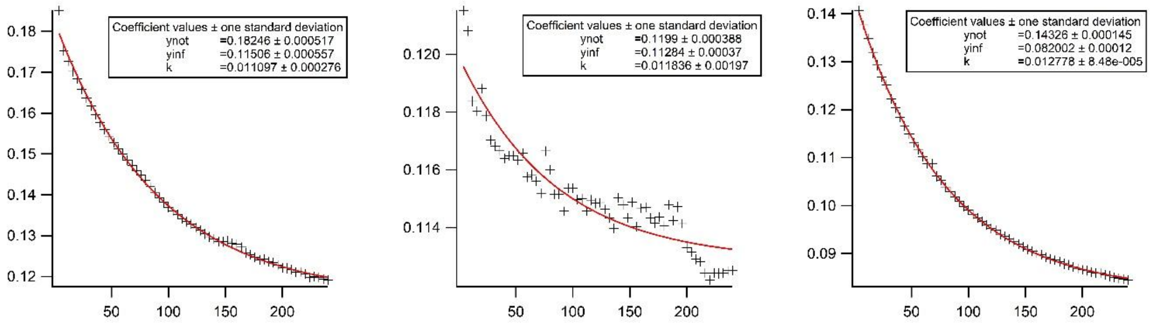

Figure S25. Kinetic traces of the conversion of 1 to 2 with 0.2 mL of 0.39 mM 1 solution in DCM with 2.0 mL of 3.91 mM O2 solution in DCM

Table S3. Concentration 2

time (s)	run 1 abs	run 2 abs	run 3 abs
5	0.11906	0.117421	0.109777
10	0.118411	0.115822	0.108293
15	0.118259	0.114502	0.107323
20	0.117998	0.11277	0.106275
25	0.117146	0.111612	0.104256
30	0.116752	0.111053	0.103004
35	0.116794	0.109476	0.102209
40	0.116175	0.108277	0.100646
45	0.117526	0.1071	0.100247
50	0.117495	0.106637	0.099231
55	0.116675	0.105222	0.098488
60	0.115302	0.104682	0.098114
65	0.115599	0.103989	0.096131
70	0.115258	0.102899	0.096341
75	0.114434	0.102814	0.094968
80	0.114461	0.101585	0.094824
85	0.113935	0.101008	0.093953
90	0.114548	0.100358	0.093311
95	0.114423	0.099694	0.092587
100	0.113883	0.099211	0.091528
105	0.114075	0.098883	0.091113
110	0.113776	0.098418	0.091289
115	0.11391	0.097833	0.091401
120	0.113645	0.097279	0.090598
125	0.113662	0.096514	0.090204
130	0.113514	0.096623	0.089488
135	0.113243	0.095819	0.089576
140	0.113126	0.095095	0.089437

145	0.112856	0.095171	0.088717
150	0.113592	0.095294	0.088397
155	0.112834	0.094836	0.088047
160	0.112843	0.094354	0.087845
165	0.113092	0.094726	0.087621
170	0.112799	0.093062	0.087064
175	0.112567	0.092708	0.086248
180	0.112404	0.092321	0.08691
185	0.112168	0.092527	0.086637
190	0.112232	0.09209	0.086028
195	0.11183	0.09172	0.086033
200	0.112096	0.091699	0.085775
205	0.113029	0.091766	0.085692
210	0.112338	0.091407	0.085149
215	0.112375	0.091078	0.085302
220	0.112234	0.091002	0.085089
225	0.111737	0.090693	0.085056
230	0.111588	0.090416	0.084904
235	0.112216	0.090329	0.084887
240	0.111883	0.090696	0.086143
245	0.11192	0.090661	0.085068
250	0.112019	0.089963	0.084561
255	0.112127	0.089708	0.085022
260	0.112022	0.08941	0.084273
265	0.110984	0.089584	0.084008
270	0.111249	0.089764	0.084373
275	0.111731	0.089222	0.083677
280	0.111044	0.088936	0.083826
285	0.111252	0.089052	0.08388
290	0.111228	0.088812	0.083808
295	0.111071	0.088939	0.083276
300	0.110886	0.088804	0.083401

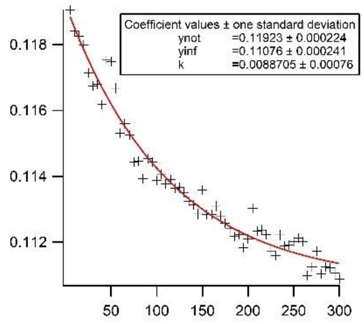

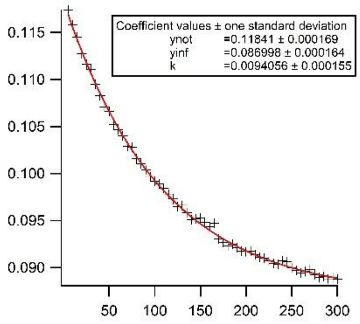

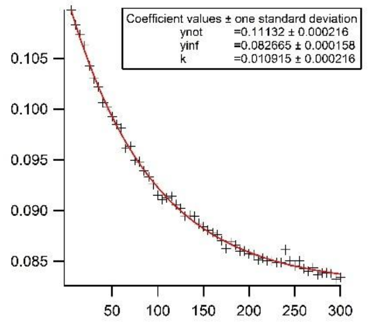

Figure S26. Kinetic traces of the conversion of 1 to 2 with 0.2 mL of 0.39 mM **1** solution in DCM with 1.10 mL of 3.91 mM O_2 solution in DCM with 0.90 mL of DCM

Table S4. Concentration 3

time (s)	run 1 abs	run 2 abs	run 3 abs
6	0.13463	0.13052	0.143453
12	0.130145	0.129124	0.140285
18	0.128433	0.127742	0.137771
24	0.127902	0.127206	0.136299
30	0.126598	0.124929	0.135954
36	0.125187	0.123677	0.133491
42	0.123754	0.123681	0.132432
48	0.122569	0.12207	0.131539
54	0.121532	0.121759	0.129523
60	0.120889	0.120943	0.1283
66	0.120147	0.120314	0.127798
72	0.119485	0.119117	0.126067
78	0.118399	0.118302	0.125016
84	0.117875	0.117161	0.12423
90	0.117198	0.116575	0.123092
96	0.116822	0.116453	0.122907
102	0.116188	0.115773	0.122593
108	0.114568	0.114399	0.120796
114	0.114427	0.114605	0.120003
120	0.113624	0.113765	0.119124
126	0.1131	0.113901	0.118493
132	0.113355	0.11296	0.11827
138	0.113059	0.113171	0.117234
144	0.111948	0.113325	0.116691
150	0.112155	0.111844	0.115846
156	0.112377	0.111656	0.115812
162	0.110666	0.110826	0.115263
168	0.110543	0.111216	0.114298
174	0.109467	0.110505	0.113957
180	0.108894	0.109864	0.113451
186	0.108876	0.11036	0.112716
192	0.108982	0.110186	0.111937
198	0.108152	0.110005	0.111575
204	0.107822	0.108812	0.111215
210	0.107668	0.10855	0.111047
216	0.107852	0.108296	0.110024
222	0.107472	0.107364	0.109952

228	0.107225	0.107688	0.109328
234	0.107022	0.107284	0.108861
240	0.106977	0.10709	0.108584
246	0.10644	0.107166	0.108213
252	0.10602	0.10681	0.107283
258	0.106208	0.107104	0.107161
264	0.105241	0.105875	0.106518
270	0.105051	0.106188	0.106097
276	0.105154	0.105371	0.106013
282	0.104334	0.105659	0.105654
288	0.104167	0.105343	0.104962
294	0.105008	0.104844	0.105234
300	0.104568	0.104797	0.104595
306	0.103572	0.105164	0.104346
312	0.103275	0.103665	0.103825
318	0.103086	0.104168	0.103452
324	0.102206	0.103633	0.103339
330	0.101173	0.10353	0.102894
336	0.101376	0.102882	0.102688
342	0.100778	0.102513	0.102308
348	0.099392	0.101887	0.102113
354	0.099104	0.100977	0.102205
360	0.099379	0.101292	0.101618

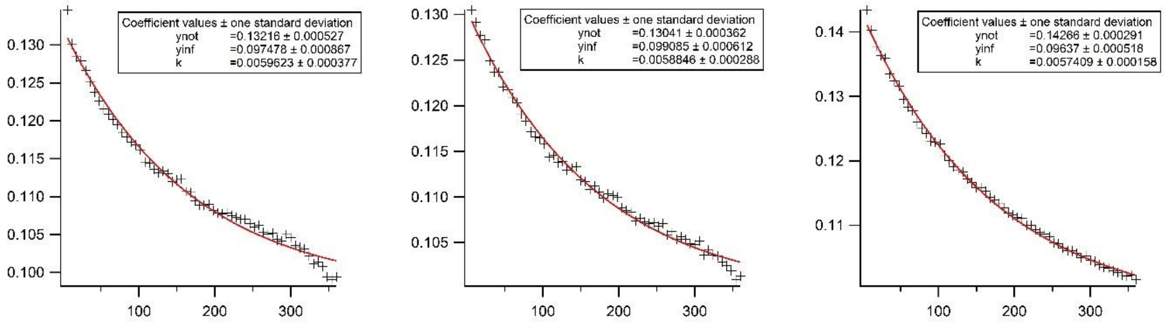

Figure S27. Kinetic traces of the conversion of 1 to 2 with 0.2 mL of 0.39 mM **1** solution in DCM with 1.55 mL of 3.91 mM O_2 solution in DCM with 0.45 mL of DCM

Table S5. Concentration 4

time (s)	run 1 abs	run 2 abs	run 3 abs
8	0.146954	0.12094	0.123465
16	0.149711	0.120604	0.121502
24	0.145196	0.119424	0.120328
32	0.146184	0.119001	0.119249
40	0.143608	0.117304	0.118208
48	0.142542	0.116289	0.11697
56	0.140744	0.116234	0.116733
64	0.139979	0.115145	0.114966
72	0.138701	0.1145	0.114823
80	0.138645	0.114028	0.114078
88	0.136816	0.11259	0.112568
96	0.137044	0.112229	0.112128
104	0.136002	0.111267	0.111053
112	0.135472	0.110032	0.110854
120	0.134736	0.11044	0.110194
128	0.133674	0.10911	0.109087
136	0.13203	0.109142	0.10865
144	0.131117	0.108287	0.107365
152	0.131819	0.107189	0.106906
160	0.129658	0.10661	0.106369
168	0.128857	0.106264	0.105577
176	0.127778	0.106015	0.10518
184	0.127583	0.10533	0.104759
192	0.126403	0.104774	0.103916
200	0.12538	0.104244	0.10368
208	0.124082	0.103804	0.103077
216	0.123432	0.103576	0.103508
224	0.122608	0.102383	0.102481
232	0.121378	0.101998	0.101791
240	0.120505	0.101617	0.100924
248	0.11985	0.10199	0.099981
256	0.119234	0.101671	0.099852
264	0.118786	0.101341	0.100027
272	0.118575	0.100473	0.099645
280	0.117131	0.100574	0.098735
288	0.116875	0.09983	0.098567
296	0.116111	0.099278	0.098448
304	0.115454	0.099124	0.097733
312	0.115646	0.099549	0.097529
320	0.114624	0.098178	0.096735
328	0.113656	0.09866	0.095974
336	0.11302	0.098061	0.09538
344	0.112416	0.097152	0.095128
352	0.111505	0.097269	0.094166

360	0.110995	0.096519	0.094342
368	0.110599	0.096139	0.093426
376	0.109818	0.096497	0.092678
384	0.10966	0.095624	0.091878
392	0.109225	0.095267	0.091691
400	0.108481	0.09552	0.091004
408	0.10755	0.094599	0.09058
416	0.107204	0.094467	0.090111
424	0.106524	0.094137	0.090102
432	0.106404	0.092982	0.089752
440	0.106186	0.093385	0.089553
448	0.105682	0.092521	0.089238
456	0.104931	0.092955	0.088902
464	0.104515	0.092338	0.08851
472	0.103776	0.091592	0.087958
480	0.103973	0.091616	0.087917

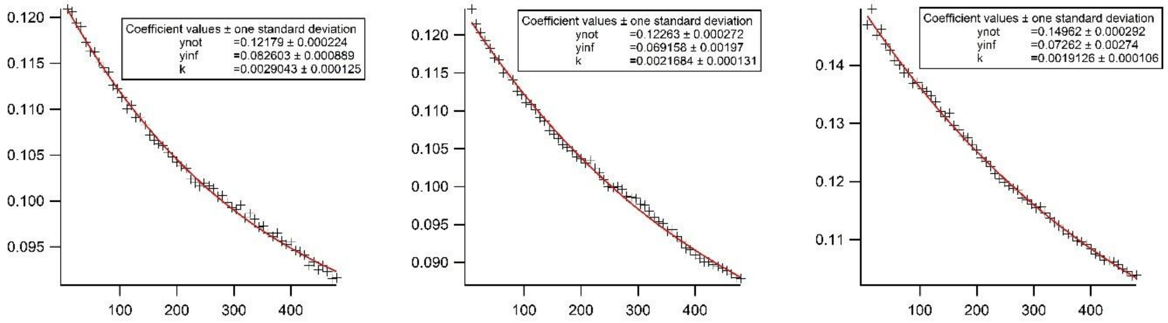

Figure S28. Kinetic traces of the conversion of 1 to 2 with 0.2 mL of 0.39 mM **1** solution in DCM with 0.65 mL of 3.91 mM O_2 solution in DCM with 1.35 mL of DCM

Table S6. Concentration 5

time (s)	run 1 abs	run 2 abs	run 3 abs
10	0.140529	0.12547	0.13765
20	0.139904	0.12535	0.13823
30	0.139015	0.12426	0.13732
40	0.138638	0.12457	0.13676
50	0.137546	0.12364	0.13625
60	0.137772	0.1236	0.13948
70	0.13549	0.12333	0.13788
80	0.135355	0.12237	0.13718
90	0.134483	0.1217	0.13584
100	0.133429	0.12112	0.1348
110	0.13258	0.12072	0.13555

120	0.132372	0.12014	0.13496
130	0.131288	0.12019	0.13503
140	0.130352	0.11962	0.13395
150	0.129611	0.11883	0.13304
160	0.129139	0.11799	0.13236
170	0.128249	0.11753	0.13272
180	0.127923	0.11699	0.13221
190	0.127043	0.11657	0.13129
200	0.126161	0.11657	0.13153
210	0.125886	0.11687	0.13059
220	0.125079	0.11521	0.13008
230	0.124758	0.11432	0.1307
240	0.123964	0.11337	0.12928
250	0.123218	0.11249	0.12911
260	0.123296	0.11204	0.12852
270	0.121657	0.1121	0.12808
280	0.121092	0.11126	0.12705
290	0.120805	0.11084	0.12672
300	0.120056	0.11029	0.12687
310	0.119331	0.11038	0.1251
320	0.118693	0.10909	0.12401
330	0.11863	0.10908	0.12311
340	0.117606	0.10809	0.12237
350	0.11677	0.10798	0.12199
360	0.115449	0.10734	0.12082
370	0.114886	0.10667	0.11991
380	0.114452	0.10648	0.11887
390	0.113965	0.10592	0.11977
400	0.113326	0.10543	0.11808
410	0.112856	0.10486	0.11751
420	0.114127	0.10392	0.11647
430	0.111833	0.10367	0.1167
440	0.11238	0.10332	0.11565
450	0.110867	0.10297	0.11551
460	0.110165	0.10213	0.11467
470	0.109874	0.10139	0.115
480	0.109848	0.10108	0.11405
490	0.109666	0.10095	0.11349
500	0.108866	0.10051	0.11261
510	0.108181	0.09962	0.11284
520	0.107568	0.09918	0.11193
530	0.107521	0.09902	0.11171
540	0.107313	0.09853	0.11103
550	0.107052	0.09814	0.11098
560	0.106342	0.09823	0.11065

570	0.10595	0.09784	0.10996
580	0.105455	0.09742	0.10956
590	0.104804	0.09703	0.1094
600	0.10427	0.09639	0.10885

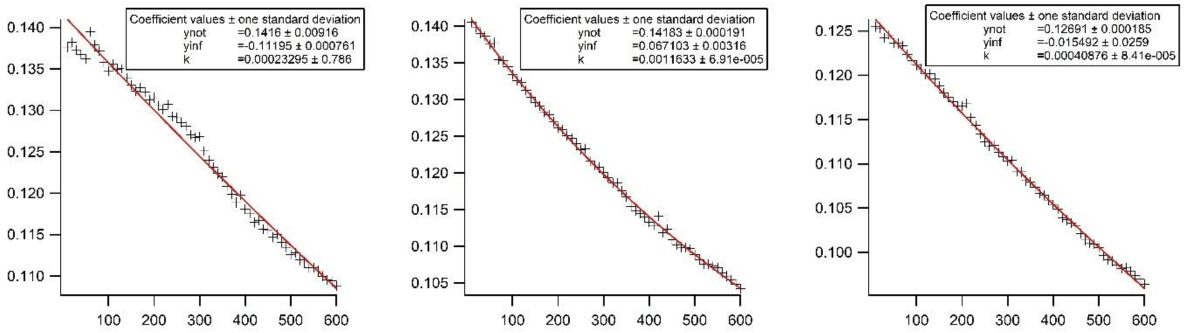

Figure S29. Kinetic traces of the conversion of 1 to 2 with 0.2 mL of 0.39 mM 1 solution in DCM with 0.20 mL of 3.91 mM O_2 solution in DCM with 1.8 mL of DCM

The resultant plot of k_{obs} vs $[O_2]$ is linear with intercept of zero. This is consistent with a first-order dependence on O_2.

Photolysis Procedure

Safety Note: LED lamps should never be directly looked at without adequate eye protection.

All photolysis was performed with a Luzchem LED Illuminator and a RGB LEDi-Head. The RGB LEDi-Head was set to the blue setting. The LEDi-Head was positioned 35 mm away from the J. Young tube or quartz UV-Vis Cuvette and set to 0.1 intensity. The J. Young tube was rotated by hand every 15 seconds for the duration of the photolysis to ensure even distribution. Photoreactions were monitored by either UV-Vis (for cuvettes) or by ^{1}H-NMR for J. Young tubes.

For the photoreaction of DCM or MeCN solutions of **2** -> **4**, all other tested wavelengths resulted in either no reaction or degradation of the sample into a mixture of uncharacterizable species. Continued photolysis of **4** with blue light slowly degrades the sample as well. For ^{1}H NMR concentration experiments, 10 minutes of photolysis at 35 mm with 0.1 intensity was sufficient to fully convert **2** -> **4**.

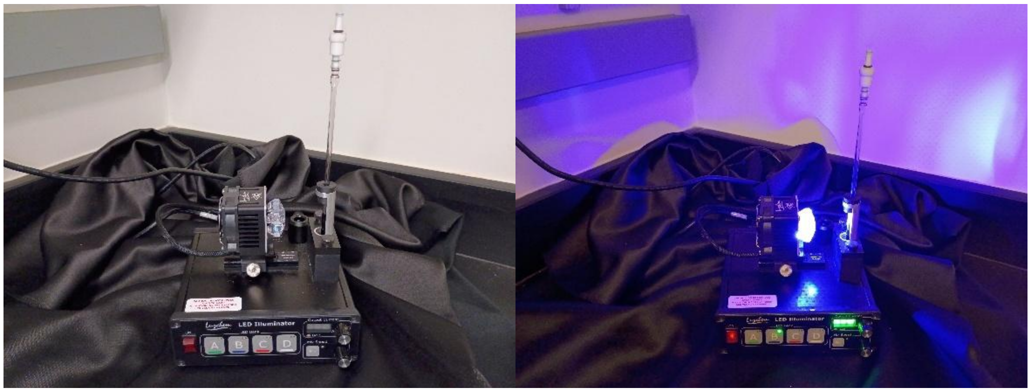

Figure S30. Photograph of LuzChem photoilluminator with NMR tube adapter.

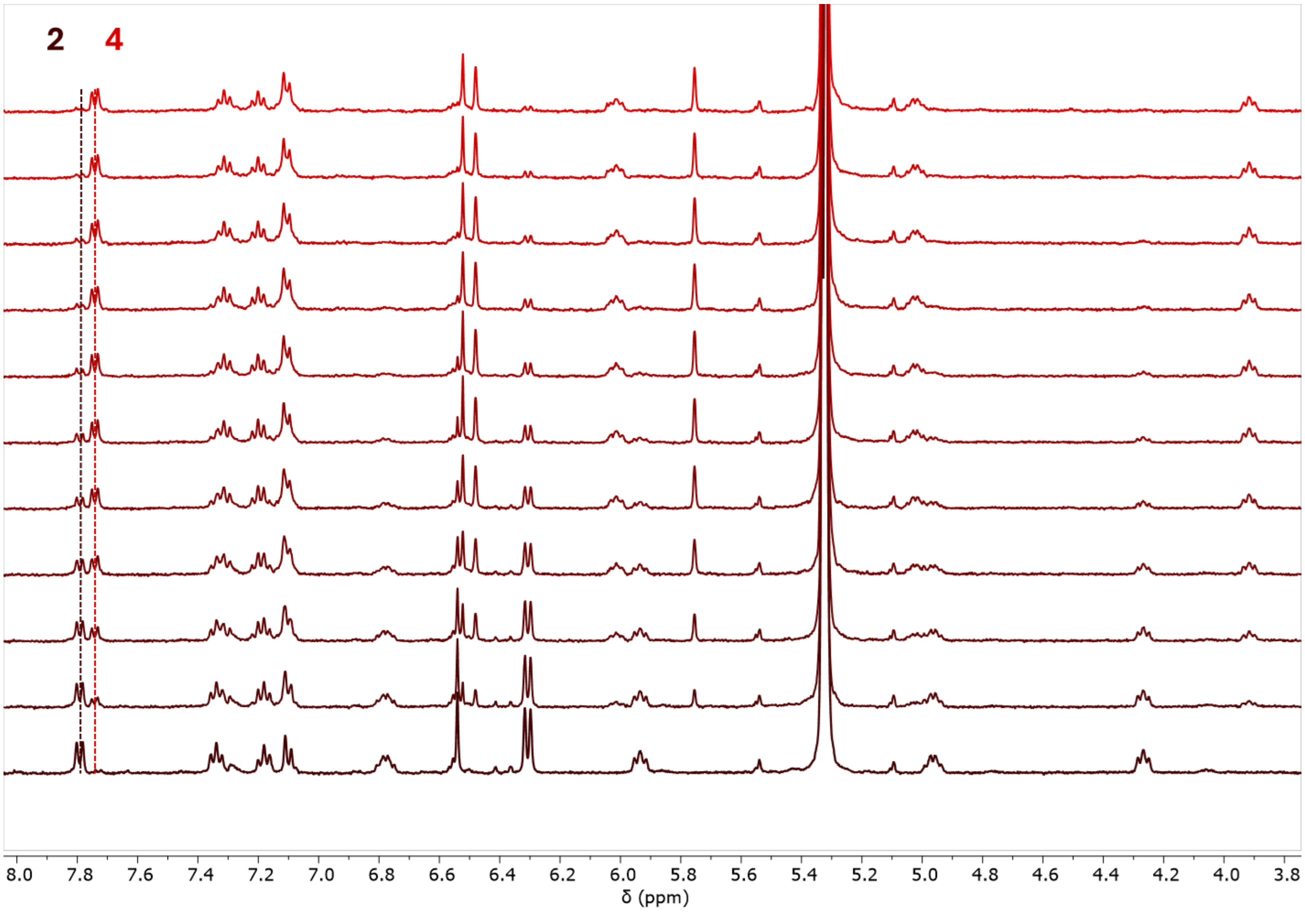

Figure S31. ^{1}H-NMR of the conversion of **2** to **4.** Bottom spectrum is **2**, each ascending slice is taken after 60 seconds of photolysis at 0.1 intensity blue light at 35 mm. Final spectrum is **4**.

Solution State IR Behavior of 1 and 2

IR spectra were collected using a Bruker ALPHA II spectrometer via transmission in a Perkin-Elmer IR liquid cell (NaCl semidemountable cell, 1 mm fixed pathlength) or attenuated total reflectance (ATR) with a diamond ATR crystal. Solutions of **1** and **2** were prepared in dry DCM. A blank spectrum (DCM in the NaCL cell) was collected and subtracted from the spectra of **1** and **2.**

The acidic protons (O–H phenol and N–H pyrazole) appear in the ^{1}H-NMR spectrum of **1** but not **2**. We wanted to ensure the protons were not appearing due to reasons of chemical exchange or H/D exchange and were still physically present on **2**, whether as protons or deuterons. Both explanations for the lack of proton signals in ^{1}H-NMR should still display O–H and N–H signals via FTIR if the samples are prepared in proteo-DCM.

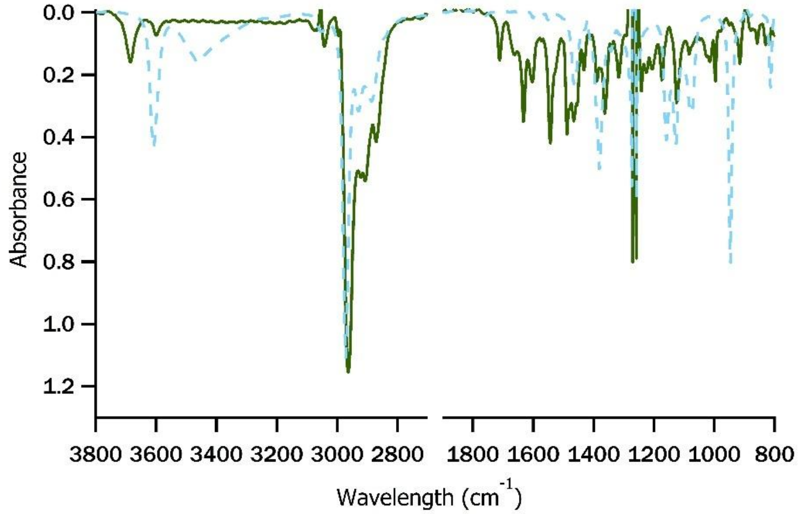

S32. FTIR spectrum of **1** (blue dashed line) and **2** (green solid line) in DCM.

The FTIR spectrum for 17 mM solutions of **1** (dashed blue) and **2** (solid green) are presented below. Both **1** and **2** have two broad features beyond 3000 cm^{-1}, at 3609 cm^{-1} & 3465 cm^{-1} for **1**, and 3686 cm^{-1} & 3601 cm^{-1} for **2**. The shifts are consistent with both the O–H and N–H proton remaining on **1** after oxygenation. Relative to **1**, the bands for **2** are decreased in intensity and blue-shifted. These changes prompted an investigation into the hydrogen bonding of these acidic protons.

A way to probe for hydrogen bonding is to collect the IR spectrum of a compound over a range of concentrations spanning ideally an order of magnitude. Once collected, the spectra can be normalized to a peak which does not display concentration dependent

behavior, such as the δ_{CH} bands. After normalization, if the shape, intensity, or wavenumber of the band changes, then concentration dependent behavior can be inferred.[7]

Stock solutions of **1** and **2** were prepared at 3 different concentrations (1.7, 5.4, and 17.0 mM) in dry DCM.

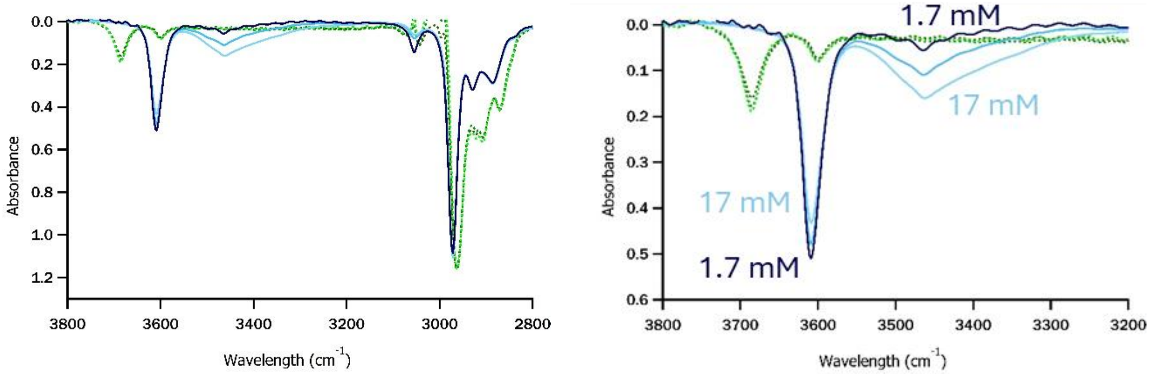

Figure S33. Left – FTIR spectrum of the >2800 cm^{-1} region of **1** (solid blue) and **2** (dashed green) from 1.7 to 17 mM solutions in DCM. Right - FTIR spectrum of the >3200 cm^{-1} region of **1** (solid blue) and **2** dashed green from 1.7 to 17 mM solutions in DCM.

The IR spectrum of **1** (blue solid lines) when normalized to the ν_{CH} band (2971 cm^{-1} and 2963 cm^{-1} for **1** and **2** respectively) changes intensity with concentration. The broad feature centered at 3465 cm^{-1} grows in relative intensity with increasing concentration while the sharper feature centered at 3606 cm^{-1} shrinks in relative intensity with increasing concentration (the darkest trace is the 1.7 mM solution of **1**). These changes in peak intensity are consistent with intermolecular hydrogen bonding.

The IR spectrum of **2** (green dashed lines) when normalized to the ν_{CH} band displays minimal concentration dependence. The features centered at 3601 and 3676 cm^{-1} – which correspond to the ν_{XH} bands – do not change meaningfully. These observations can be interpretted either of two ways: (1) the acidic protons of **2** participate in intramolecular hydrogen bonding, that is minimally in this concentration regime; or (2) the acidic protons of **2** are not hydrogen bonding in solution. It is not important to differentiate which of the two circumstances are operative in the present work.

^{18}O-**2** and ^{16}O-**2** IR

IR spectra were collected using a Bruker ALPHA II spectrometer via transmission in a Perkin-Elmer IR liquid cell (NaCl semidemountable cell, 1 mm fixed pathlength) or attenuated total reflectance (ATR) with a diamond ATR crystal. Solutions of **1** were prepared

in benzene and either oxygenated with ^{16}O gas or ^{18}O gas (gifted with much thanks from the Bollinger-Krebs group) to generate a solution of **^{16}O-2** or **^{18}O-2**. For FTIR experiments, a blank spectrum of benzene was collected and subtracted from the solutions of **^{16}O-2** and **^{18}O-2**.

The two absolute spectra were compared, and a difference spectra was generated by performing a normalization procedure (normalized to the δ_{CH} bands at 2959 cm^{-1}; Figure S34). Two perturbations are the most prevalent, one band at 984 cm^{-1} (**2-^{16}O**) that moves to 971 cm^{-1} (**2-^{18}O**); and 760 cm^{-1} (**2-^{16}O**) and 752 cm^{-1} (**2-^{18}O**). The reduced mass equation suggests that the two bands should differ by a ratio of 1.06. Side-bound peroxo stretches are reported to appear between 800-930 cm^{-1}.[8]

In the absence of agreement between theory and experiment, we are reluctant to assign the O–O stretch unambiguously. However, the data is included for the interested reader, as it is possible that alternative treatments of the data would yield less ambiguous conclusions.

The challenges in assigning the O–O stretch in this case can be attributed to low signal-to-noise. These challenges could reflect a small perturbation to the dipole moment for the proposed O–O stretch.

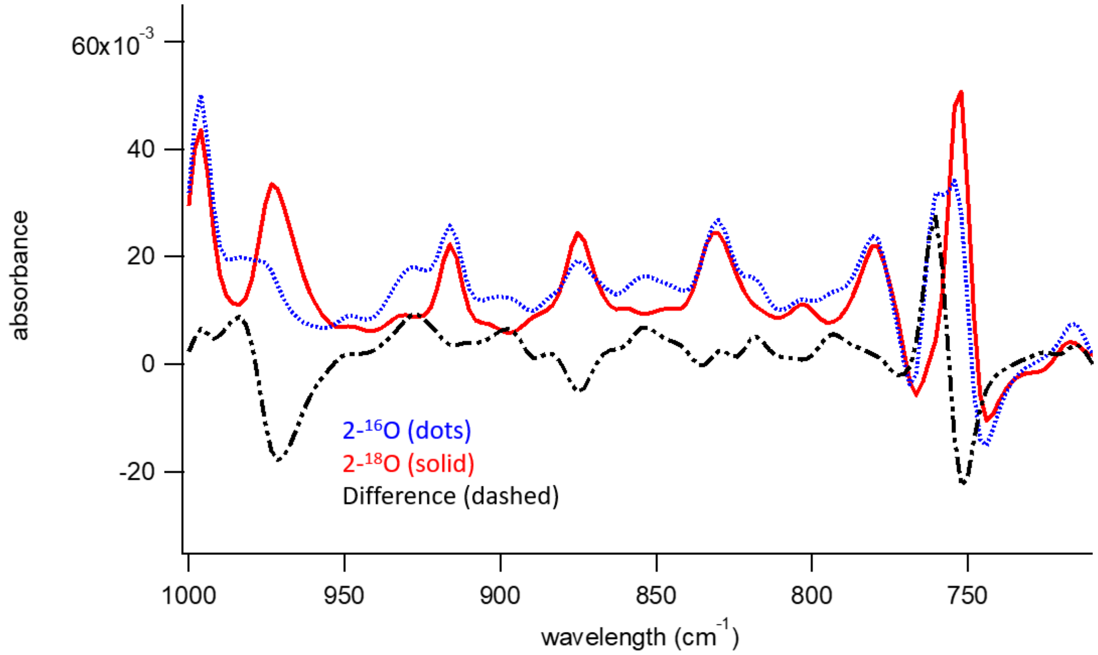

Figure S34. FTIR spectrum of the O–O region of **2**-^{16}O (blue dots) and **2**-^{18}O (red dots) solutions in benzene The difference spectrum (black dashes) is overlaid.

EPR of **3** and **5**

1 mM solutions of **3** and **5** were prepared by dissolving **3** and **5** in a 50/50 solvent mixture of MeCN/THF followed by immediate flash freezing in liquid nitrogen to create a MeCN/THF glass.

We analyzed the EPR spectra using a Magnettech MS-5000X spectrometer equipped with an Oxford ESR900 variable-temperature helium-flow cryostat. A LakeShore 335 Cryogenic Controller controlled the temperature. The following experimental conditions were used: Temperature = 70K, Microwave frequency = 9.436 GHz, Microwave power = 1 mW, Modulation amplitude = 1 mT, Scan time = 60s with 4096 points. We accumulated 10 scans to obtain the data shown. We performed data processing and spectral simulation using MATLAB. For data processing, we utilized the Kazan Viewer software package (within Matlab).[9] Processing included subtraction of an EPR spectrum of a solvent-only sample acquired under identical conditions and a 3rd -order polynomial baseline subtraction. For spectral simulation and data visualisation, we used purpose-built scripts that employed the "pepper" utility from the EasySpin software package.[10]

The EPR of **5** contains a species which is similar to the EPR of **3**. To rule out contamination of **3** as the source of the signal, crystals of **5** were dissolved in CD_3CN. As noted in [relationship of **2** and **3**] and [relationship of **4** and **5**], dissolving crystals of **5** in MeCN results in a clean spectrum of **4**. No trace of **2** was detectable by proton NMR, which is consistent with no contaimination by **3.**

DFT Calculations for **3** and **5**

DFT calculations were performed using ORCA 6.0 using TPSSh functional, def2-TZVP basis set[10] on all atoms with def2/J auxiliary basis set for RIJ-COSX RI approximaton .[11] Conductor-like Polarizable Continuum Model (CPCM)[12] was used to account for solvent with default settings for acetonitrile (dielectric constant = 36.7630, refractive index=1.3442). For Ir, default Effective Core Potentials (ECPs) (Def2-ECP) were used to treat relativistic effects.[13] Geometry optimization was performed using crystallograpgic data as a starting structure without any constraints. Then, we used the optimized structure in a single-point calculation to obtain Mulliken spin population values and spin density distribution.

To support the assignment of **3** and **5** as ligand based radicals on Ir(III) centers, structural optimizations of **3** and **5** were performed (**3opt** and **5opt**) so the bond metrics could be compared to the X-Ray structures. Mulliken spin populations were also calculated to locate the unpaired electron density. To serve as a comparison, the one electron oxidized structures of **3** and **5** were calculated. Theses structures (**3ox** and **5ox**) can be thought of as bona fide

quinones. Neutral paramagnetic cofactors were used as a starting point to ensure the closest comparison.

3opt

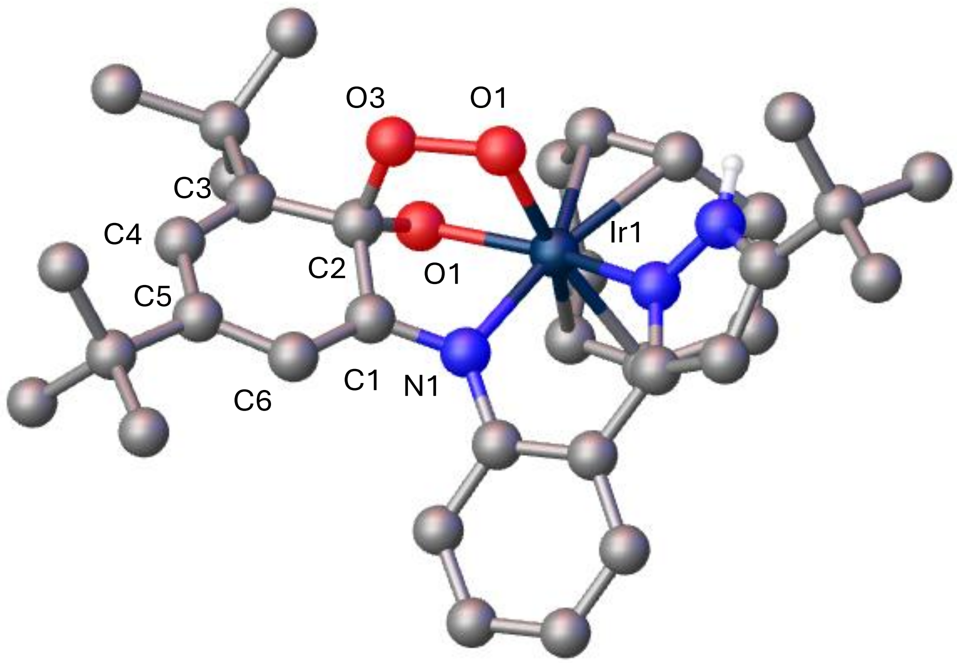

Figure S35. Structure of **3opt**

Table S7. Selected Bond Lengths (Å) of **3opt**

Ir1-O1	2.035
Ir1-O2	2.034
Ir1-N1	2.056
O2-O3	1.492
O1-C2	1.395
O3-C2	1.474
N1-C1	1.366
C1-C2	1.512
C2-C3	1.513
C3-C4	1.370

C4-C5	1.428
C5-C6	1.399
C6-C1	1.389

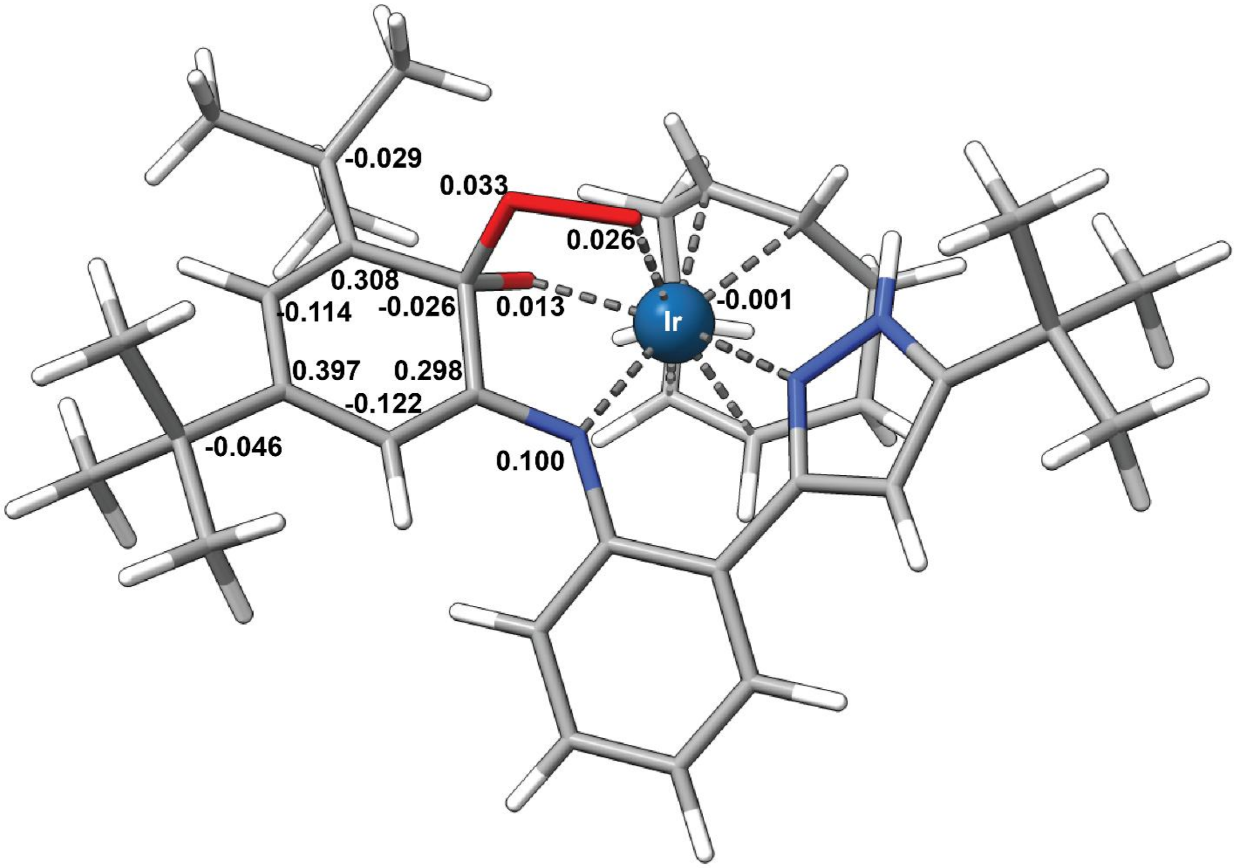

Figure S36. Mulliken Spin Population of **3opt**

Table S8. XYZ coordinates of **3Opt**

Ir1	7.051845	6.527333	3.267682
O1	8.774226	7.769385	5.226685
O2	6.636994	6.882088	5.227723
O3	8.64351	7.701743	3.742142
N1	8.086253	5.01651	4.202013
N2	8.210857	5.970948	1.605509
N3	8.70683	6.78611	0.650186
H1	8.442479	7.758656	0.640305
C1	4.420885	8.030584	3.793453
H2	3.68391	8.765306	3.445653
H3	4.540796	8.179973	4.865791
C2	3.918297	6.602103	3.528612
H4	3.316512	6.567697	2.618427
H5	3.262705	6.297308	4.346892
C3	5.045581	5.588758	3.416747
H6	5.165399	4.922576	4.264363
C4	5.608053	5.17875	2.209853
H7	6.071274	4.198335	2.183361
C5	5.261631	5.761083	0.858271
H8	4.312212	5.331821	0.514969
H9	6.022163	5.434155	0.146979
C6	5.1784	7.301255	0.842022
H10	5.42989	7.655019	-0.15977
H11	4.155479	7.62974	1.036856
C7	6.105938	7.982822	1.838976
H12	6.903013	8.586039	1.415616
C8	5.747885	8.320205	3.140857
H13	6.344126	9.087926	3.62339
C9	8.269588	3.75473	3.658492
C10	8.142235	2.586465	4.43576
H14	7.894528	2.684873	5.484886
C11	8.295556	1.329383	3.875579
H15	8.171421	0.450634	4.49906
C12	8.5862	1.190331	2.515181
H16	8.701701	0.208377	2.072254
C13	8.713441	2.328209	1.733912
H17	8.928556	2.232347	0.675555
C14	8.563023	3.612584	2.276748

C15	8.78813	4.76889	1.415426
C16	9.643415	4.848333	0.29797
H18	10.25777	4.055141	-0.09208
C17	9.577623	6.149578	-0.16549
C18	10.28108	6.847929	-1.30625
C19	9.240358	7.443495	-2.27641
H20	8.613579	8.189723	-1.78195
H21	8.594396	6.661318	-2.6825
H22	9.757098	7.932013	-3.10628
C20	11.17492	7.976264	-0.75108
H23	11.92247	7.577009	-0.06132
H24	10.5856	8.731273	-0.22478
H25	11.69392	8.466456	-1.57864
C21	11.15343	5.833041	-2.05822
H26	11.66013	6.33685	-2.8845
H27	10.54921	5.021142	-2.47044
H28	11.91479	5.402483	-1.4031
C22	7.893369	6.746406	5.817883
C23	7.932744	7.045178	7.300836
C24	8.816345	6.323199	8.058702
H29	8.921931	6.586225	9.104399
C25	9.592896	5.218893	7.594472
C26	9.35936	4.742903	6.300274
H30	9.857068	3.849414	5.947382
C27	8.468597	5.394963	5.457653
C28	10.58938	4.559905	8.548785
C29	9.83879	3.967709	9.761988
H31	9.288749	4.738467	10.30743
H32	10.54903	3.504521	10.45447
H33	9.126335	3.203402	9.438972
C30	11.60107	5.610212	9.054742
H34	12.16693	6.03527	8.220858
H35	12.3089	5.14426	9.747633
H36	11.10203	6.427735	9.580349
C31	11.37827	3.427756	7.875664
H37	10.72292	2.617222	7.545227
H38	12.094	3.009185	8.588831
H39	11.93767	3.79146	7.009244
C31	7.030206	8.122893	7.910599
C32	6.993297	9.410003	7.062161
H40	7.98456	9.865865	6.999339
H41	6.313839	10.12885	7.531979
H42	6.644074	9.204773	6.051363

C33	7.496548	8.522001	9.32177
H43	7.433475	7.690637	10.02862
H44	6.848494	9.320812	9.693382
H45	8.525108	8.893592	9.315391
C34	5.587922	7.576712	8.04169
H46	5.181795	7.313007	7.065225
H47	4.944853	8.336969	8.4981
H48	5.570549	6.687299	8.678332

3ox

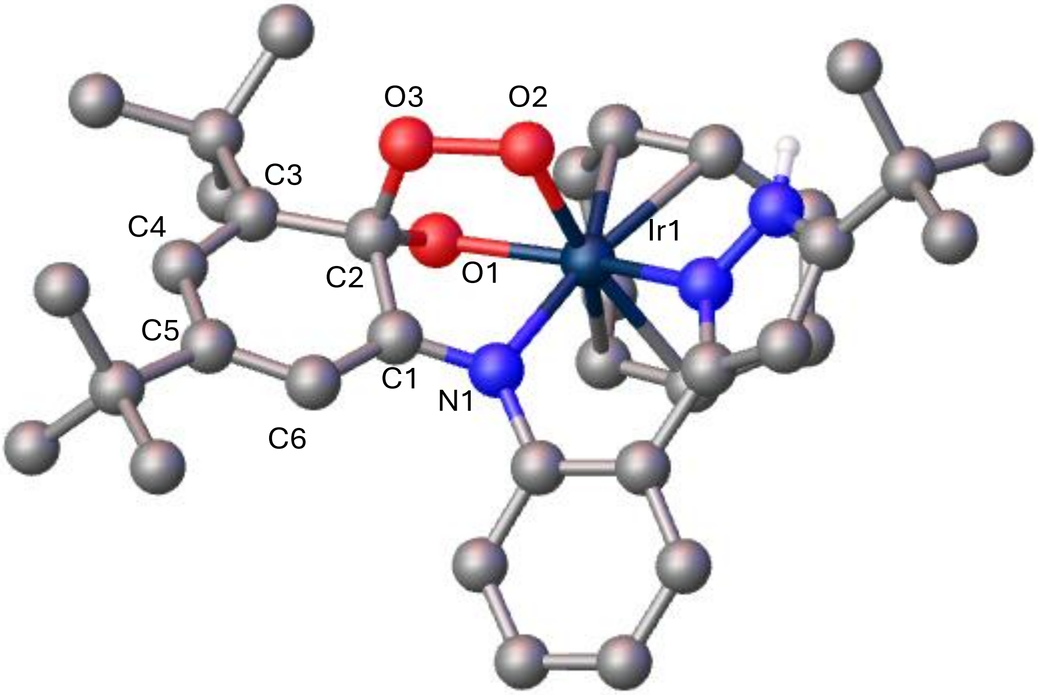

Figure S37. Structure of **3ox**

Table S9. Selected Bond Lengths (Å) of **3ox**

Ir1-O1	2.037
Ir1-O2	2.044
Ir1-N1	2.059
O2-O3	1.477
O1-C2	1.381
O3-C2	1.448
N1-C1	1.310
C1-C2	1.530

C2-C3	1.525
C3-C4	1.347
C4-C5	1.459
C5-C6	1.363
C6-C1	1.420

Table S10. XYZ coordinates of **3ox**

Ir1	7.03555	6.49048	3.273214
O1	8.805883	7.708236	5.209706
O2	6.6415	6.87149	5.235457
O3	8.671902	7.623105	3.741481
N1	8.067454	4.989805	4.233926
N2	8.189942	5.94909	1.605138
N3	8.654621	6.771648	0.642086
H1	8.307403	7.71668	0.584073
C1	4.426215	8.024412	3.817678
H2	3.696792	8.766243	3.470024
H3	4.553165	8.178763	4.888129
C2	3.90505	6.60094	3.557664
H4	3.292882	6.572757	2.654713
H5	3.256814	6.305391	4.384477
C3	5.015877	5.571282	3.436244
H6	5.134386	4.904568	4.283971
C4	5.569009	5.154493	2.226796
H7	6.024894	4.169823	2.200758
C5	5.225624	5.738476	0.876393
H8	4.272383	5.314177	0.539151
H9	5.981256	5.405899	0.163093
C6	5.149903	7.278782	0.8606
H10	5.398624	7.63119	-0.14191
H11	4.131506	7.614527	1.063591
C7	6.088106	7.954837	1.849563
H12	6.89481	8.546291	1.428033
C8	5.745912	8.306827	3.150263
H13	6.356319	9.069423	3.623059
C9	8.308249	3.719521	3.66854
C10	8.123006	2.564021	4.43429
H14	7.79824	2.657837	5.462713
C11	8.325006	1.310302	3.875021
H15	8.171618	0.424266	4.479497
C12	8.7076	1.197094	2.540094

H16	8.863187	0.221647	2.0954
C13	8.865111	2.34021	1.769148
H17	9.135513	2.250944	0.724006
C14	8.658474	3.616078	2.308729
C15	8.849429	4.783861	1.449748
C16	9.720478	4.89196	0.353009
H18	10.38882	4.129394	-0.00796
C17	9.579154	6.176344	-0.14276
C18	10.25558	6.887735	-1.29155
C19	9.197505	7.351335	-2.31369
H20	8.491363	8.05663	-1.86874
H21	8.636837	6.499565	-2.70617
H22	9.696536	7.851413	-3.14726
C20	11.03495	8.107652	-0.75774
H23	11.78912	7.798548	-0.03009
H24	10.36989	8.832243	-0.28136
H25	11.53913	8.605097	-1.58987
C21	11.23223	5.921039	-1.97601
H26	11.72151	6.434724	-2.80646
H27	10.71052	5.04714	-2.37387
H28	12.0053	5.580116	-1.28288
C22	7.897123	6.746388	5.796885
C23	7.945054	7.047496	7.291518
C24	8.760386	6.281743	8.04266
H29	8.855581	6.506203	9.096063
C25	9.54246	5.150595	7.554741
C26	9.358263	4.690042	6.285642
H30	9.883211	3.821482	5.915028
C27	8.458226	5.37282	5.424587
C28	10.51844	4.501876	8.523847
C29	9.729296	3.926868	9.723097
H31	9.180545	4.701079	10.26303
H32	10.43183	3.46241	10.41998
H33	9.018795	3.166214	9.390078
C30	11.50963	5.573417	9.031586
H34	12.08825	5.989557	8.203071
H35	12.20252	5.109269	9.738074
H36	11.00104	6.392036	9.544515
C31	11.31918	3.365059	7.876647
H37	10.66992	2.55242	7.540496
H38	12.01495	2.955268	8.612497
H39	11.90136	3.721464	7.022773
C31	7.10672	8.178069	7.885981

C32	7.119066	9.443877	7.006543
H40	8.129685	9.846724	6.90981
H41	6.490831	10.20274	7.481653
H42	6.728438	9.241077	6.010742
C33	7.609855	8.576125	9.284059
H43	7.507396	7.763653	10.00756
H44	7.007459	9.414203	9.64296
H45	8.655901	8.892749	9.258553
C34	5.646821	7.682979	8.035692
H46	5.217559	7.419835	7.069642
H47	5.045902	8.480053	8.483606
H48	5.602586	6.808652	8.690326

5opt

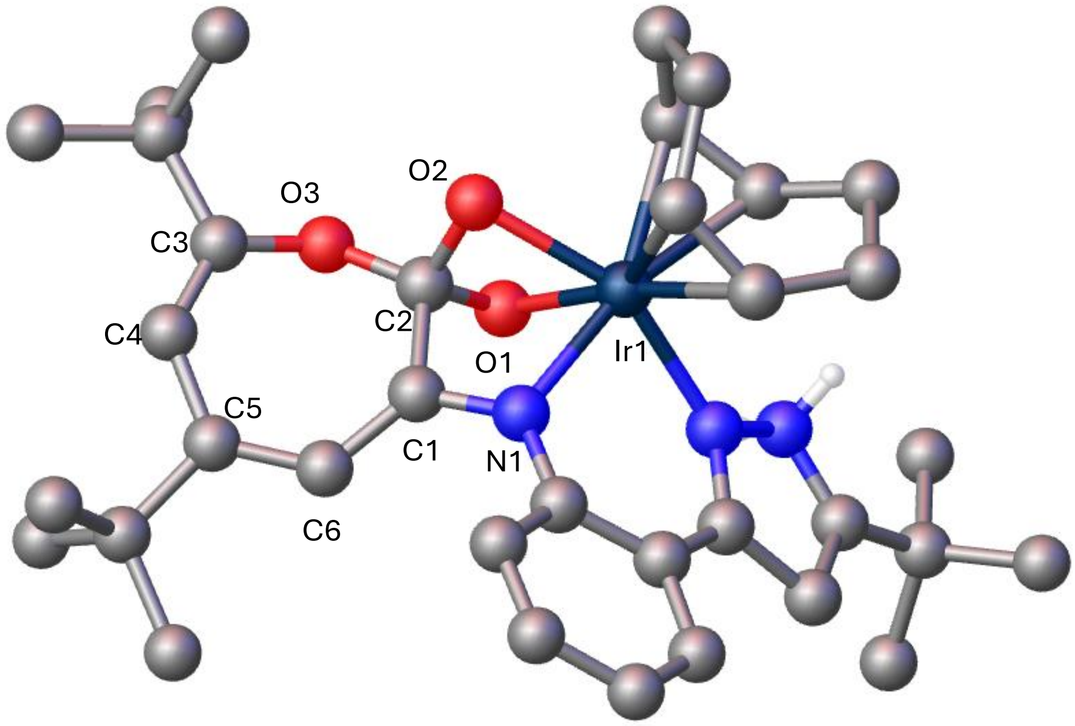

Figure S38. Structure of **5opt**

Table S11. Selected Bond Lengths (Å) of **5opt**

Ir1-O1	2.065
Ir1-O2	2.083

Ir1-N1	2.076
O1-C2	1.395
O2-C2	1.390
N1-C1	1.334
C1-C2	1.536
C2-O3	1.415
O3-C3	1.370
C3-C4	1.353
C4-C5	1.442
C5-C6	1.375
C6-C1	1.410

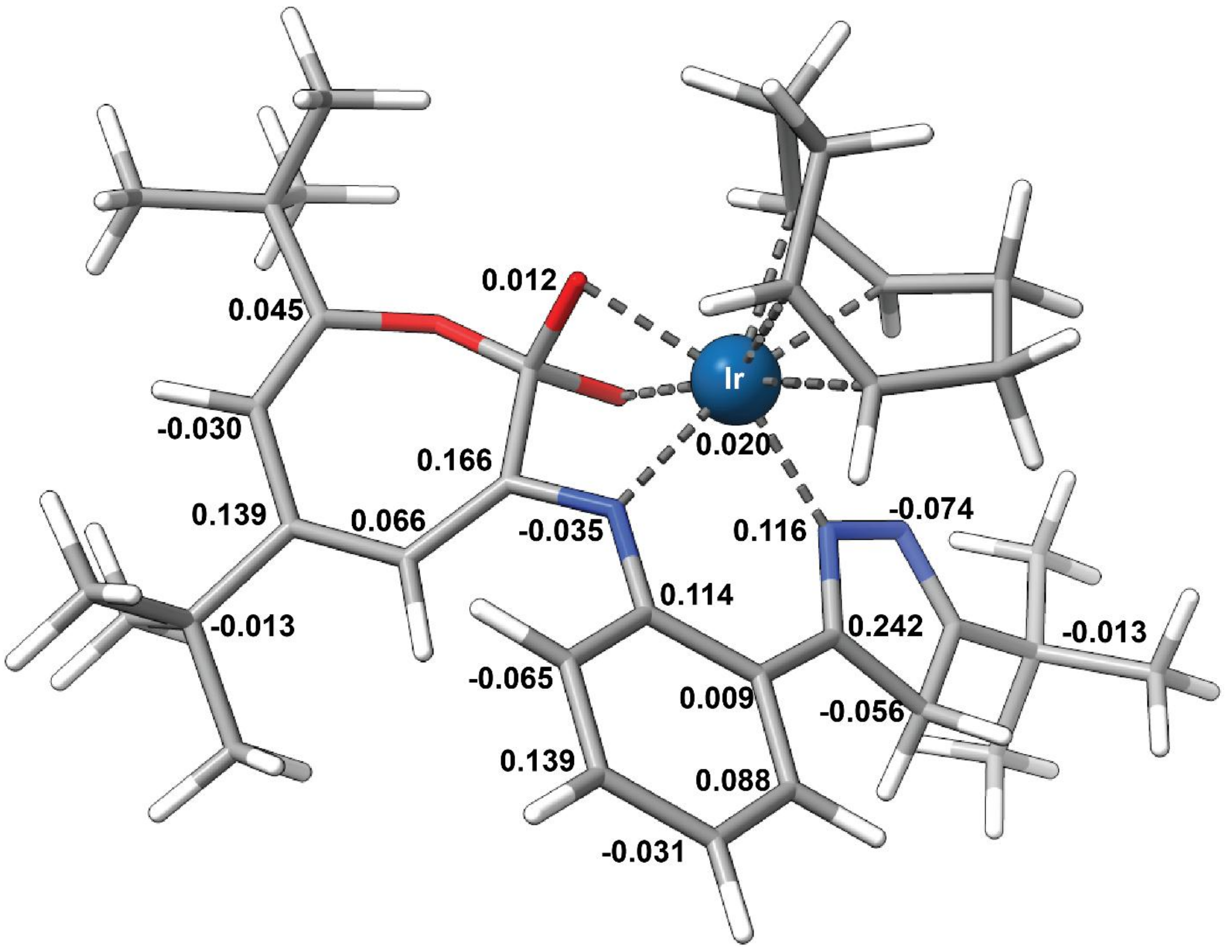

Figure S39. Mulliken Spin Population of **5opt**

Table S12. XYZ coordinates of **5opt**

Ir1	7.301584	8.070345	12.55438
O1	8.456576	7.219702	9.096481
O2	7.564326	8.749282	10.60274
O3	8.368884	6.805848	11.31917
N1	5.798666	7.077997	11.52111
N2	6.94154	6.560745	13.94413
N3	7.897729	6.100439	14.85548
C1	8.808715	8.773662	14.05081
H1	9.355739	7.869989	14.28671
C2	8.179701	9.489744	15.236
H2	8.452506	10.54607	15.19868
H3	8.619052	9.08733	16.15115
C3	6.649457	9.328036	15.30038
H4	6.405356	8.431861	15.87362
H5	6.199734	10.17185	15.8382
C4	6.00805	9.198529	13.93782
H6	5.039079	8.70966	13.93296
C5	6.320576	10.00417	12.83339
H7	5.593885	10.02662	12.02784
C6	7.284332	11.18072	12.87595
H8	7.280678	11.60889	13.8801
H9	6.907952	11.9558	12.20532
C7	8.711589	10.79507	12.45111
H10	8.804444	10.88631	11.36879
H11	9.442008	11.48202	12.89594
C8	9.069649	9.375894	12.82288
H12	9.812587	8.903663	12.18773
C9	7.495417	5.110327	18.17042
H13	8.02445	4.61505	18.98888
H14	6.432885	4.871206	18.25851
H15	7.618788	6.190439	18.28342
C10	9.570714	4.959813	16.76622
H16	9.742131	6.034085	16.8614
H17	10.01435	4.624542	15.82619
H18	10.0821	4.454336	17.58954
C11	7.884014	3.100387	16.71236

H19	8.426227	2.608203	17.52405
H20	8.272093	2.725036	15.76191
H21	6.830403	2.822217	16.79078
C12	8.074435	4.628474	16.82252
C13	7.327582	5.304749	15.6997
C14	5.858462	5.164718	15.42149
H22	5.55919	4.128435	15.23007
H23	5.246606	5.509906	16.26547
C15	5.733718	6.026164	14.20095
C16	4.562795	6.186844	13.41378
C17	3.30636	5.771779	13.93171
H24	3.264811	5.385443	14.94351
C18	4.588631	6.688202	12.07002
C19	2.147179	5.867007	13.19389
H25	1.204453	5.553781	13.62756
C20	3.393071	6.781685	11.33953
H26	3.429459	7.197838	10.34
C21	2.184936	6.387902	11.8876
H27	1.272284	6.494206	11.31299
C22	6.274058	6.788919	10.30914
C23	5.659033	5.929888	9.374934
H28	4.840481	5.342411	9.772145
C24	5.928371	5.836717	8.030293
C25	6.907104	6.637933	7.337052
H29	6.776352	6.703391	6.26584
C26	8.009037	7.270619	7.803044
C27	7.68006	7.405521	10.26516
C28	5.108987	4.885096	7.143195
C29	6.064934	3.983667	6.333032
H30	6.728852	4.565901	5.690884
H31	5.482222	3.310958	5.697121
H32	6.681872	3.375849	7.000646
C30	4.240672	5.709901	6.16673
H33	3.543177	6.348104	6.715578
H34	3.661266	5.031451	5.533875
H35	4.844909	6.342852	5.513262
C31	4.173795	3.970615	7.947184
H36	4.72586	3.366061	8.671924
H37	3.667292	3.290411	7.257568
H38	3.404628	4.535311	8.48021
C32	9.007719	8.012006	6.923482
C33	8.624673	7.972195	5.438964
H39	8.592231	6.948224	5.058002

H40	9.37673	8.520154	4.86583
H41	7.654947	8.442665	5.256637
C34	10.40224	7.368342	7.086919
H42	10.73504	7.404767	8.124873
H43	11.12505	7.911483	6.472234
H44	10.38874	6.324787	6.760793
C35	9.079576	9.485414	7.37606
H45	8.113764	9.981145	7.245776
H46	9.822189	10.01491	6.772908
H47	9.368212	9.560306	8.425358

5ox

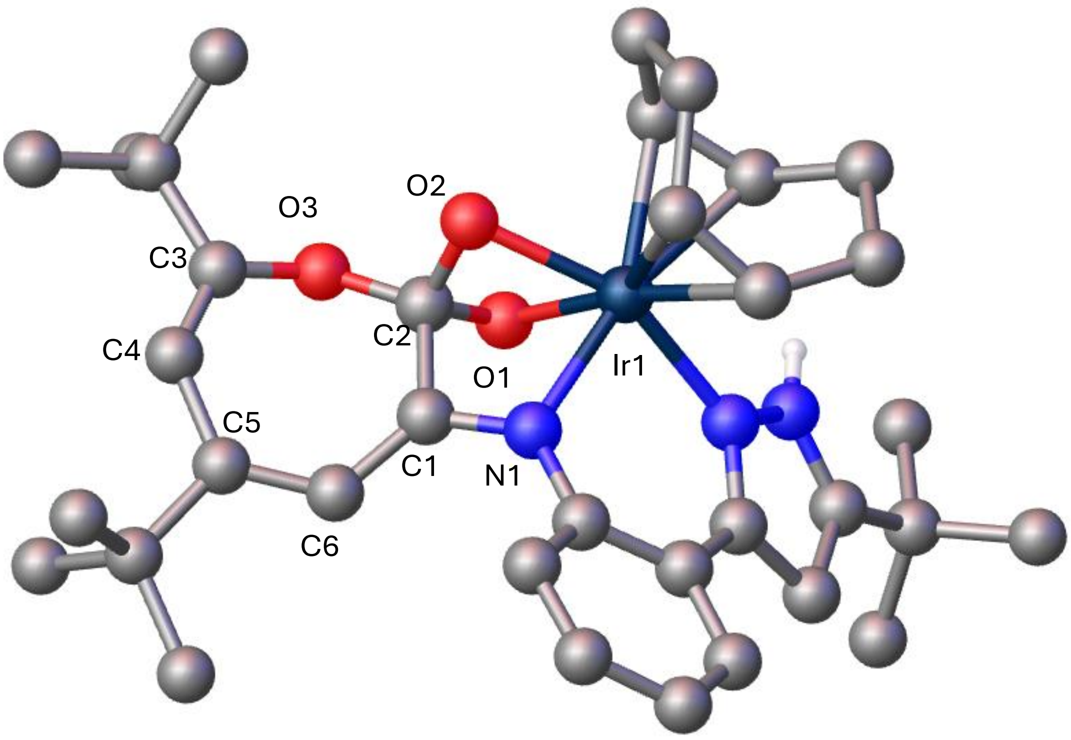

Figure S40. Structure of **5ox**

Table S13. Selected Bond Lengths (Å) of **5ox**

Ir1-O1	2.049
Ir1-O2	2.059
Ir1-N1	2.066
O1-C2	1.388
O2-C2	1.393

N1-C1	1.317
C1-C2	1.539
C2-O3	1.411
O3-C3	1.363
C3-C4	1.353
C4-C5	1.442
C5-C6	1.370
C6-C1	1.415

Table S14. XYZ coordinates of **5ox**

Ir1	7.202015	8.081691	12.55962
O1	8.479228	7.040568	9.219157
O2	7.492678	8.649279	10.60177
O3	8.323955	6.771585	11.4529
N1	5.773844	6.973853	11.55982
N2	6.869529	6.685001	14.07165
N3	7.895747	6.259768	14.95385
C1	8.665138	8.960887	14.04058
H1	9.243226	8.095801	14.33887
C2	7.982609	9.720049	15.16643
H2	8.213316	10.78203	15.06797
H3	8.421045	9.390677	16.11021
C3	6.458556	9.500116	15.22374
H4	6.239868	8.634417	15.85095
H5	5.970381	10.35831	15.7007
C4	5.833042	9.2683	13.86859
H6	4.889931	8.730915	13.88288
C5	6.125153	10.00751	12.71981
H7	5.415546	9.93961	11.90165
C6	7.053465	11.21066	12.6926
H8	7.026428	11.6992	13.66767
H9	6.658067	11.92724	11.97061
C7	8.498421	10.85266	12.30144
H10	8.597263	10.87072	11.21654
H11	9.194556	11.60007	12.70042
C8	8.921904	9.486562	12.78089
H12	9.692037	9.002585	12.18853
C9	7.651234	5.084938	18.13033
H13	8.220062	4.601238	18.92802
H14	6.601801	4.80168	18.23824
H15	7.737307	6.16744	18.24887

C10	9.704605	5.02895	16.67878
H16	9.835049	6.106884	16.79064
H17	10.13633	4.726268	15.7222
H18	10.25382	4.529306	17.4802
C11	8.08346	3.103179	16.63926
H19	8.673099	2.625808	17.42516
H20	8.451399	2.75345	15.6715
H21	7.044579	2.785438	16.75417
C12	8.224716	4.633235	16.76688
C13	7.42509	5.301334	15.67935
C14	6.01128	4.986802	15.31333
H22	5.887024	3.95979	14.95451
H23	5.31743	5.110705	16.15125
C15	5.781563	5.976474	14.22367
C16	4.554518	6.105066	13.45316
C17	3.339962	5.694274	14.02255
H24	3.329773	5.3129	15.03583
C18	4.551766	6.586012	12.12305
C19	2.150737	5.794205	13.31618
H25	1.221249	5.487941	13.7798
C20	3.350354	6.689817	11.42128
H26	3.354694	7.09582	10.41803
C21	2.15722	6.299876	12.01764
H27	1.229958	6.396685	11.46544
C22	6.260187	6.668745	10.37487
C23	5.629961	5.865153	9.395518
H28	4.785918	5.284942	9.743491
C24	5.907045	5.870386	8.053449
C25	6.936194	6.651886	7.413933
H29	6.827479	6.772089	6.346291
C26	8.081887	7.164022	7.920755
C27	7.667519	7.29141	10.34543
C28	5.034096	5.044953	7.096696
C29	5.940012	4.144878	6.227772
H30	6.642369	4.725449	5.627346
H31	5.314206	3.560326	5.548465
H32	6.510227	3.451997	6.852067
C30	4.24124	6.007767	6.183384
H33	3.594557	6.660045	6.775691
H34	3.613172	5.420581	5.508247
H35	4.897778	6.631471	5.573867
C31	4.029534	4.140849	7.824411
H36	4.531419	3.443351	8.500103

H37	3.484284	3.554937	7.080635
H38	3.296439	4.716135	8.395459
C32	9.170932	7.816664	7.083856
C33	8.785178	7.927451	5.604007
H39	8.624747	6.945956	5.151296
H40	9.601076	8.415366	5.065615
H41	7.883575	8.529888	5.466953
C34	10.45576	6.965604	7.201221
H42	10.78813	6.889631	8.237415
H43	11.25031	7.436064	6.616488
H44	10.29064	5.958127	6.810577
C35	9.449746	9.231321	7.633841
H45	8.564958	9.867044	7.544586
H46	10.25858	9.684881	7.055488
H47	9.749827	9.196051	8.681611

Crystallographic Data

1

A solution of **1** was prepared in Et_2O. Single crystals were obtained by slow reverse vapor diffusion of MeCN into the Et_2O solution at room temperature, followed by washing with room temperature MeCN and 0° C pentane.

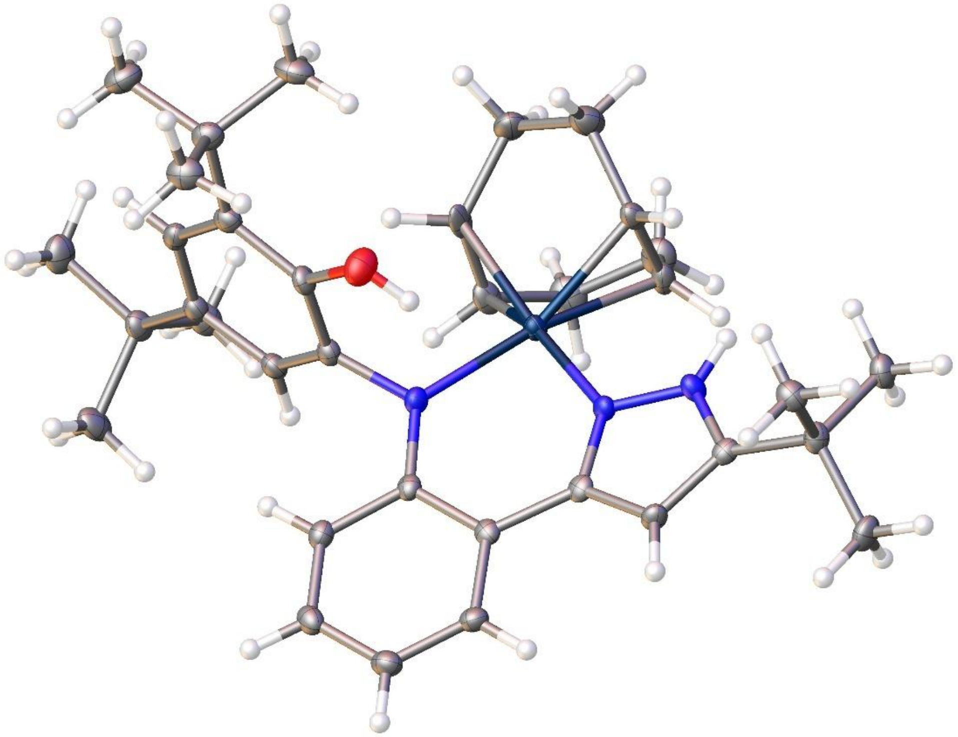

Figure S41. X-Ray crystal structure of **1**

Table S15. Crystallographic information of **1**

Identification code	Ir(I) sq. pl.
Empirical formula	$C_{35}H_{48}IrN_3O$
Formula weight	718.96
Temperature/K	100
Crystal system	monoclinic
Space group	$P2_1/c$
a/Å	15.8835(9)
b/Å	17.6398(10)
c/Å	11.5570(7)
α/°	90
β/°	106.896(2)
γ/°	90
Volume/Å^3	3098.3(3)
Z	4
ρ_{calc}g/cm^3	1.541
μ/mm^{-1}	4.341

F(000)	1456.0
Crystal size/mm^3	0.12 × 0.08 × 0.06
Radiation	MoKα (λ = 0.71073)
2Θ range for data collection/°	2.68 to 55.066
Index ranges	-20 ≤ h ≤ 20, -22 ≤ k ≤ 22, -14 ≤ l ≤ 8
Reflections collected	50835
Independent reflections	7122 [R_{int} = 0.0527, R_{sigma} = 0.0339]
Data/restraints/parameters	7122/0/371
Goodness-of-fit on F^2	1.042
Final R indexes [I>=2σ (I)]	R_1 = 0.0269, wR_2 = 0.0538
Final R indexes [all data]	R_1 = 0.0411, wR_2 = 0.0577
Largest diff. peak/hole / e $Å^{-3}$	1.49/-0.67

Table S16. Bond length metrics for **1**

Ir1	7268.5(2)	5401.2(2)	5058.0(2)	13.48(4)
O1	7195.3(18)	6309.8(15)	2336(3)	27.1(6)
N1	6140.7(18)	5919.1(16)	5128(2)	14.3(6)
N2	6106.9(19)	6423.9(16)	6019(3)	16.6(6)
N3	6581.5(18)	5118.1(16)	3309(3)	15.8(6)
C1	5288(2)	6689.3(19)	5889(3)	14.1(7)
C2	4760(2)	6338.3(19)	4874(3)	15.6(7)
C3	5304(2)	5865.4(19)	4424(3)	14.1(7)
C4	5054(2)	5415.5(19)	3311(3)	14.0(6)
C5	4152(2)	5356(2)	2711(3)	17.3(7)
C6	3833(2)	5011(2)	1592(3)	19.9(8)
C7	4429(2)	4703(2)	1057(3)	18.6(7)
C8	5320(2)	4739.8(18)	1629(3)	16.4(7)
C9	5671(2)	5089.4(19)	2772(3)	14.1(7)
C10	5110(2)	7315(2)	6675(3)	17.3(7)
C11	4236(2)	7159(2)	6945(3)	22.3(8)
C12	5059(2)	8067(2)	5974(3)	20.6(8)
C13	5845(2)	7358(2)	7879(3)	20.3(8)
C14	7115(2)	4973.1(19)	2523(3)	15.1(7)
C15	7454(2)	5606.1(19)	2071(3)	15.8(7)
C16	8078(2)	5536(2)	1439(3)	17.1(7)

C17	8327(2)	4799(2)	1241(3)	16.8(7)
C18	7992(2)	4152(2)	1645(3)	16.9(7)
C19	7378(2)	4257(2)	2293(3)	16.6(7)
C20	8501(2)	6244(2)	1056(3)	19.8(7)
C21	7785(2)	6720(2)	176(3)	25.1(9)
C22	8977(3)	6702(2)	2193(4)	26.9(9)
C23	9179(2)	6040(2)	405(4)	27.6(9)
C24	8281(2)	3341(2)	1442(3)	19.8(8)
C25	8653(2)	2948(2)	2675(4)	23.9(8)
C26	8981(3)	3333(2)	775(4)	27.0(9)
C27	7477(2)	2904(2)	673(4)	27.2(9)
C28	8396(3)	4200(2)	6626(4)	28.1(9)
C29	8478(3)	4842(2)	7525(4)	26.3(9)
C30	7842(2)	5483(2)	6958(3)	19.6(7)
C31	8077(2)	6118(2)	6381(3)	17.9(7)
C32	8971(2)	6242(2)	6180(4)	26.6(9)
C33	9301(2)	5534(2)	5691(4)	25.6(9)
C34	8544(2)	5074(2)	4892(3)	21.4(8)
C35	8144(2)	4469(2)	5322(3)	21.1(8)

3

A solution of **2** was prepared in MeCN and to it was added 10 equivalents of TEMPO • to drive the formation of **3**. Single crystals were obtained by slow reverse vapor diffusion of MeCN into DMF at room temperature. Crystals were washed with 0° C MeCN and left on high vacuum overnight to sublime away excess TEMPO•.

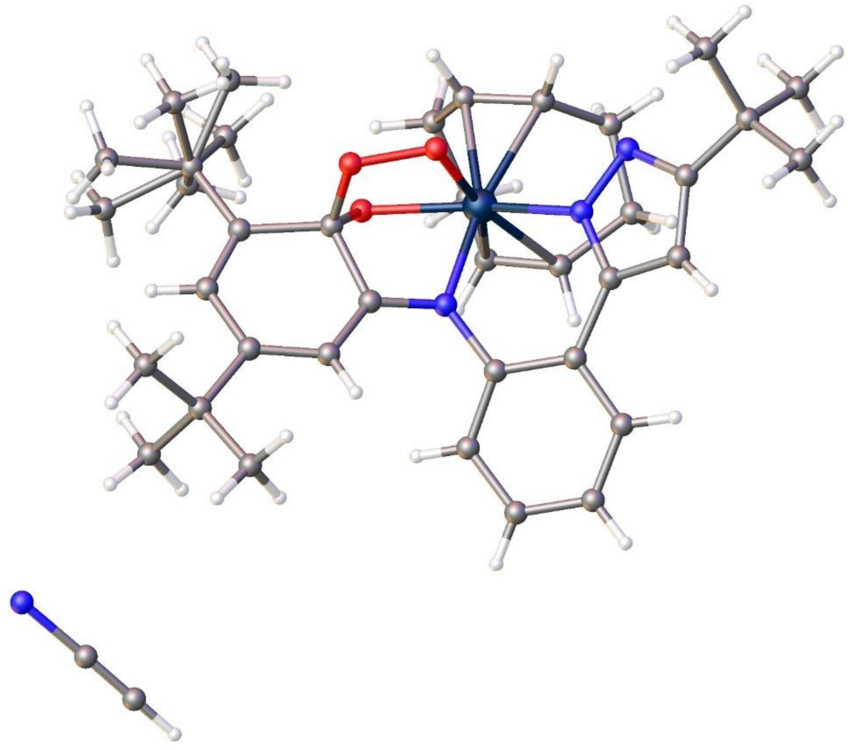

Figure S43. X-Ray crystal structure of **3**

Table S17. Crystallographic information of **3**

Identification code	aga1_auto (1)
Empirical formula	$C_{37}H_{47}IrN_4O_3$
Formula weight	787.98
Temperature/K	293(2)
Crystal system	monoclinic
Space group	$P2_1$
a/Å	11.7532(2)
b/Å	9.4819(2)
c/Å	15.6273(3)
α/°	90
β/°	95.850(2)
γ/°	90
Volume/Å^3	1732.48(6)
Z	2
ρ_{calc}g/cm^3	1.511

μ/mm^{-1}	7.776
F(000)	796.0
Crystal size/mm^3	0.1 × 0.08 × 0.02
Radiation	Cu Kα (λ = 1.54184)
2Θ range for data collection/°	5.684 to 148.016
Index ranges	-13 ≤ h ≤ 14, -11 ≤ k ≤ 10, -18 ≤ l ≤ 19
Reflections collected	12490
Independent reflections	5869 [R_{int} = 0.0263, R_{sigma} = 0.0292]
Data/restraints/parameters	5869/1/448
Goodness-of-fit on F^2	1.051
Final R indexes [I>=2σ (I)]	R_1 = 0.0306, wR_2 = 0.0805
Final R indexes [all data]	R_1 = 0.0308, wR_2 = 0.0807
Largest diff. peak/hole / e Å^{-3}	1.09/-1.02
Flack parameter	0.252(13)

Table S18. Bond length metrics of **3**

**Atom**	***x***	***y***	***z***	**U(eq)**
Ir1	6103.8(2)	6836.1(7)	2097.6(2)	19.68(10)
O28	7641(6)	8145(7)	3428(4)	29.6(14)
O30	5785(4)	7234(5)	3340(3)	24.7(10)
O46	7488(4)	8050(5)	2483(3)	26.9(10)
N1	7012(5)	5293(7)	2752(4)	23.9(12)
N2	7025(4)	6349(6)	1085(3)	21.5(11)
N3	7236(5)	7249(6)	455(3)	26.4(13)
C1	3869(7)	8422(9)	2274(5)	40.9(18)
C2	3453(5)	6948(16)	2085(5)	43.1(18)
C3	4407(6)	5835(9)	2088(5)	33.0(16)
C4	4881(6)	5430(9)	1346(5)	27.1(16)
C5	4573(9)	6049(12)	448(7)	29(2)
C6	4522(11)	7668(14)	448(7)	38(3)
C7	5301(6)	8379(8)	1133(5)	26.0(15)
C8	4995(6)	8749(8)	1942(5)	30.8(15)
C9	7259(5)	3948(7)	2401(4)	22.4(12)
C10	7164(6)	2725(8)	2878(5)	30.1(15)
C11	7408(7)	1413(8)	2546(5)	36.9(18)

**Atom**	***x***	***y***	***z***	**U(eq)**
C12	7696(7)	1322(8)	1718(5)	34.8(16)
C13	7724(6)	2512(8)	1223(5)	29.0(14)
C14	7507(5)	3854(7)	1542(4)	22.2(12)
C15	7542(5)	5090(7)	973(4)	24.2(13)
C16	8101(5)	5199(8)	228(4)	26.8(13)
C17	7895(5)	6564(7)	-67(4)	26.3(19)
C18	8311(7)	7310(9)	-835(5)	38.1(18)
C19	7296(9)	7613(13)	-1485(6)	60(3)
C20	8835(16)	8716(17)	-548(8)	120(7)
C21	9153(9)	6413(13)	-1253(7)	74(4)
C22	6882(5)	7133(6)	3747(4)	25.1(18)
C23	6943(6)	7438(8)	4728(4)	29.2(14)
C24	7606(6)	6572(8)	5251(4)	28.6(19)
C25	8242(5)	5377(7)	4984(4)	26.6(13)
C26	8128(6)	4952(7)	4157(4)	25.6(13)
C27	7365(8)	5673(9)	3553(6)	21.1(18)
C28	8988(6)	4571(8)	5686(4)	32.7(15)
C29	8198(9)	3961(16)	6300(7)	80(4)
C30	9832(9)	5566(12)	6191(7)	65(3)
C31	9643(10)	3395(12)	5330(7)	69(3)
C32	6335(7)	8718(9)	5050(4)	37.0(17)
C33A	6437(13)	10059(13)	4442(8)	43(4)
C33B	7120(30)	9920(30)	5110(20)	69(10)
C34A	6857(14)	9204(17)	5946(8)	48(4)
C34B	6040(40)	8330(40)	6060(20)	78(13)
C35A	5091(12)	8390(20)	5072(10)	50(4)
C35B	5150(20)	9060(30)	4560(20)	56(10)
N4	10114(8)	1970(20)	8099(7)	95(4)
C42	9626(12)	-180(20)	7137(9)	83(4)
C43	9828(9)	760(20)	7549(9)	74(4)

5

A solution of **4** was prepared in MeCN and to it was added 10 equivalents of TEMPO • to drive the formation of **5**. Single crystals were obtained by slow reverse vapor diffusion of MeCN into DMF at room temperature. Crystals were washed with 0° C MeCN and left on high vacuum overnight to sublime away excess TEMPO•.

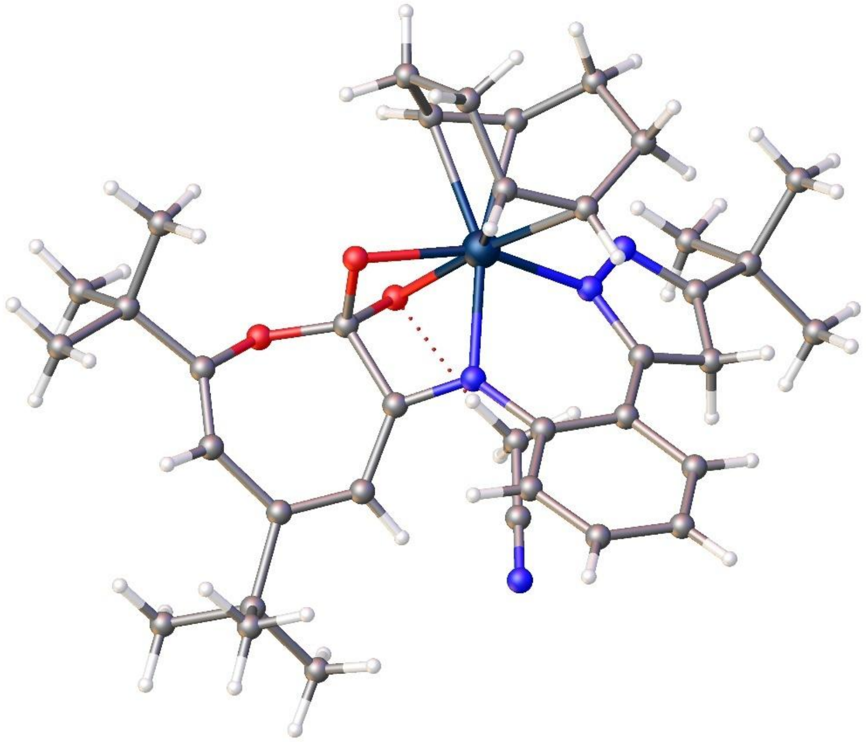

Figure S44. X-Ray crystal structure of **5**

Table S19. Crystallographic information of **5**

Identification code	AGA2_auto (6)
Empirical formula	$C_{37}H_{50}IrN_4O_3$
Formula weight	791.01
Temperature/K	173.00(10)
Crystal system	orthorhombic
Space group	$P2_12_12_1$
a/Å	9.3556(3)
b/Å	11.5960(4)
c/Å	31.7162(10)
α/°	90

β/°	90
γ/°	90
Volume/Å^3	3440.8(2)
Z	4
ρ_{calc}g/cm^3	1.527
μ/mm^{-1}	7.831
F(000)	1604.0
Crystal size/mm^3	0.05 × 0.04 × 0.03
Radiation	Cu Kα (λ = 1.54184)
2Θ range for data collection/°	8.118 to 151.88
Index ranges	-11 ≤ h ≤ 8, -12 ≤ k ≤ 14, -38 ≤ l ≤ 35
Reflections collected	13717
Independent reflections	6196 [R_{int} = 0.0405, R_{sigma} = 0.0509]
Data/restraints/parameters	6196/6/417
Goodness-of-fit on F^2	1.067
Final R indexes [I>=2σ (I)]	R_1 = 0.0376, wR_2 = 0.0971
Final R indexes [all data]	R_1 = 0.0408, wR_2 = 0.0986
Largest diff. peak/hole / e Å^{-3}	1.59/-1.70
Flack parameter	-0.037(8)

Table S20. Bond length metrics of **5**

Atom	*x*	*y*	*z*	U(eq)
Ir1	7870.9(4)	6790.4(3)	3932.5(2)	19.96(12)
O1	9115(7)	5912(6)	2864(2)	32.2(16)
O2	8188(7)	7302(5)	3318(2)	22.2(14)
O3	9037(7)	5648(6)	3582(2)	28.6(16)
N1	6283(9)	5896(7)	3614(2)	25.9(17)
N2	7454(7)	5641(6)	4411(2)	19.7(16)
N3	8382(9)	5329(7)	4716(2)	27.4(18)
C1	9510(11)	7434(8)	4390(3)	25(2)
C2	8826(10)	8076(10)	4753(3)	28(2)
C3	7204(12)	7971(8)	4777(3)	28(2)
C4	6501(12)	7831(8)	4350(3)	27(2)
C5	6847(10)	8471(7)	3990(3)	28(2)
C6	7865(13)	9469(7)	3985(3)	33(2)

Atom	*x*	*y*	*z*	U(eq)
C7	9384(12)	9115(9)	3857(3)	37(3)
C8	9796(10)	7912(8)	3993(3)	31(2)
C9	8604(16)	5037(11)	5689(4)	52(3)
C10	9959(18)	3709(16)	5226(5)	86(6)
C11	7579(19)	3113(12)	5543(4)	83(6)
C12	8443(13)	4093(9)	5351(3)	36(2)
C13	7700(12)	4598(8)	4976(3)	29(2)
C14	6277(11)	4445(8)	4843(3)	26(2)
C15	6162(10)	5122(7)	4477(3)	21.9(19)
C16	4880(10)	5247(8)	4211(3)	23.0(19)
C17	3502(11)	4974(8)	4368(3)	27(2)
C18	4947(9)	5580(8)	3782(3)	22(2)
C19	2293(12)	5037(8)	4124(3)	33(2)
C20	3727(11)	5653(8)	3535(3)	28(2)
C21	2393(10)	5390(9)	3698(3)	31(2)
C22	6806(9)	5598(7)	3251(3)	19.2(18)
C23	6200(10)	4843(7)	2946(3)	23.1(19)
C24	6419(11)	4920(8)	2528(3)	24.1(19)
C25	7353(10)	5794(8)	2336(3)	26(2)
C26	8534(12)	6261(9)	2485(3)	31(2)
C27	8314(10)	6131(8)	3229(3)	26(2)
C28	5560(12)	4155(8)	2222(3)	29(2)
C29	6525(13)	3768(10)	1854(4)	45(3)
C30	4307(13)	4875(11)	2054(4)	49(3)
C31	4984(15)	3076(12)	2437(4)	50(3)
C32	9501(12)	7096(9)	2254(4)	41(3)
C33	8890(16)	7406(13)	1813(4)	62(4)
C34	10953(14)	6539(13)	2193(5)	61(4)
C35	9626(14)	8200(11)	2507(4)	52(3)
N4	5780(20)	2474(12)	3634(4)	78(4)
C36	6840(20)	2713(12)	3792(5)	61(5)
C37	8150(16)	2987(10)	3986(4)	61(4)

Works Cited

1. Sipps, K. D.; Gibbs, W. A.; Sayfutyarova, E. R.; Kuo, J. L., Shapeshifting Ligands Mask Lewis Acidity of Dicationic Palladium(II). *ACS Catalysis* **2024,** *14* (23), 18045-18054.
2. Jain, A. K.; Gau, M. R.; Carroll, P. J.; Goldberg, K. I., Comparing Square-Planar RhI and IrI: Metal–Ligand Proton Tautomerism, Fluxionality, and Reactivity. *Organometallics* **2022,** *41* (22), 3341-3348.
3. Kuo, J. L.; Goldberg, K. I., Metal/Ligand Proton Tautomerism Facilitates Dinuclear H2 Reductive Elimination. *Journal of the American Chemical Society* **2020,** *142* (51), 21439-21449.
4. Bhatti, T. M.; Kumar, A.; Parihar, A.; Moncy, H. K.; Emge, T. J.; Waldie, K. M.; Hasanayn, F.; Goldman, A. S., Metal–Ligand Proton Tautomerism, Electron Transfer, and C(sp3)–H Activation by a 4-Pyridinyl-Pincer Iridium Hydride Complex. *Journal of the American Chemical Society* **2023,** *145* (33), 18296-18306.
5. Rao, N.; Kuo, J. L., Net Oxidative Addition of H2 to {MII}2+ (M = Pd, Pt) by Heterolysis and Protic Rebound. *Journal of the American Chemical Society* **2025,** *147* (26), 22351-22357.
6. Espenson, *Chemical kinetics and reaction mechanisms / by James H. Espenson*. 2nd ed. ed.; McGraw-Hill: 1995.
7. Hansen, P. E.; Spanget-Larsen, J. NMR and IR Investigations of Strong Intramolecular Hydrogen Bonds *Molecules* [Online], 2017, p. 552.
8. Cramer, C. J.; Tolman, W. B.; Theopold, K. H.; Rheingold, A. L., Variable character of O—O and M—O bonding in side-on (η2) 1:1 metal complexes of O2. *Proceedings of the National Academy of Sciences* **2003,** *100* (7), 3635-3640.
9. https://github.com/AlexeySilakov/KazanViewer created by Boris Epel and Alexey Silakov.
10. Stoll, S.; Schweiger, A., EasySpin, a comprehensive software package for spectral simulation and analysis in EPR. *Journal of Magnetic Resonance* **2006,** *178* (1), 42-55.
11. Weigend, F., Accurate Coulomb-fitting basis sets for H to Rn. *Physical Chemistry Chemical Physics* **2006,** *8* (9), 1057-1065.
12. Barone, V.; Cossi, M., Quantum Calculation of Molecular Energies and Energy Gradients in Solution by a Conductor Solvent Model. *The Journal of Physical Chemistry A* **1998,** *102* (11), 1995-2001.
13. Andrae, D.; Häußermann, U.; Dolg, M.; Stoll, H.; Preuß, H., Energy-adjusted ab initiopseudopotentials for the second and third row transition elements. *Theoretica chimica acta* **1990,** *77* (2), 123-141.
